# Prescribing systemic steroids for acute respiratory tract infections in United States outpatient settings: A nationwide population-based cohort study

**DOI:** 10.1371/journal.pmed.1003058

**Published:** 2020-03-31

**Authors:** Kueiyu Joshua Lin, Evan Dvorin, Aaron S. Kesselheim

**Affiliations:** 1 Division of Pharmacoepidemiology and Pharmacoeconomics, Department of Medicine, Brigham and Women’s Hospital, Harvard Medical School, Boston, Massachusetts, United States of America; 2 Department of Medicine, Massachusetts General Hospital, Harvard Medical School, Boston, Massachusetts, United States of America; 3 Ochsner Health System, Jefferson Parish, Louisiana, United States of America; Stanford University, UNITED STATES

## Abstract

**Background:**

Evidence and guidelines do not support use of systemic steroids for acute respiratory tract infections (ARTIs), but such practice appears common. We aim to quantify such use and determine its predictors.

**Methods and findings:**

We conducted a cohort study based on a large United States national commercial claims database, the IBM MarketScan, to identify patients aged 18–64 years with an ARTI diagnosis (acute bronchitis, sinusitis, pharyngitis, otitis media, allergic rhinitis, influenza, pneumonia, and unspecified upper respiratory infections) recorded in ambulatory visits from 2007 to 2016. We excluded those with systemic steroid use in the prior year and an extensive list of steroid-indicated conditions, including asthma, chronic obstructive pulmonary disease, and various autoimmune diseases. We calculated the proportion receiving systemic steroids within 7 days of the ARTI diagnosis and determined its significant predictors. We identified 9,763,710 patients with an eligible ARTI encounter (mean age 39.6, female 56.0%) and found 11.8% were prescribed systemic steroids (46.1% parenteral, 47.3% oral, 6.6% both). All ARTI diagnoses but influenza predicted receiving systemic steroids. There was high geographical variability: the adjusted odds ratio (aOR) of receiving parenteral steroids was 14.48 (95% confidence interval [CI] 14.23–14.72, *p* < 0.001) comparing southern versus northeastern US. The corresponding aOR was 1.68 (95% CI 1.66–1.69, *p* < 0.001) for oral steroids. Other positive predictors for prescribing included emergency department (ED) or urgent care settings (versus regular office), otolaryngologist/ED doctors (versus primary care), fewer comorbidities, and older patient age. There was an increasing trend from 2007 to 2016 (aOR 1.93 [95% CI 1.91–1.95] comparing 2016 to 2007, *p* < 0.001). Our findings are based on patients between 18 and 64 years old with commercial medical insurance and may not be generalizable to older or uninsured populations.

**Conclusions:**

In this study, we found that systemic steroid use in ARTI is common with a great geographical variability. These findings call for an effective education program about this practice, which does not have a clear clinical net benefit.

## Abstract 2

Joshua Lin and colleagues reveal systemic steroid prescriptions for acute respiratory tract infections are increasing in the United States.

## Author summary

**Why was this study done?:**

- Prescribing systemic (oral or injection) steroids for acute respiratory tract infections (ARTIs), a practice lacking clear medical justification, has been identified as common in the US.
- However, prior studies of this issue have not addressed time trend or details of steroid use.

**What did the researchers do and find?:**

- In this cohort study including 9,763,710 patients with an eligible ARTI encounter, 11.8% were prescribed systemic steroids.
- There was remarkable geographical variability: patients in the southern US were 14-fold more likely to receive steroid injections for ARTI than those in the Northeast.
- The prescribing rate of systemic steroids for ARTI almost doubled from 2007 to 2016.

**What do these findings mean?:**

- Systemic steroid use in ARTI is common with increasing trend over time and great geographical variability.
- These findings call for an effective medical education program to reduce this practice.

## Introduction

Using systemic corticosteroids in the treatment of acute respiratory tract infections (ARTIs) in the outpatient settings is not recommended by clinical guidelines [1–3]. Data from randomized control trials (RCTs) show that systemic steroids are ineffective in the treatment of lower respiratory tract infections [4]. Similar—albeit more limited—data also show the lack of effectiveness of steroid use in the common cold [5] and otitis media [6]. Studies have shown mixed results on whether systemic steroids lead to faster symptom relief in pharyngitis [7], and possibly also in sinusitis [8].

By contrast, one meta-analysis of RCTs showed even a short course of systemic steroids in sinusitis with polyposis could result in a 3-fold increase in the risk of gastrointestinal disturbances and insomnia [9]. An observational study found that acute adverse events associated with short-term use of systemic steroids, including sepsis, venous thromboembolism, and fracture, can occur as early as the first 30 days of drug exposure [10]. Taken together, available evidence and professional society recommendations do not support prescribing systemic steroids for ARTI in ambulatory settings [1–3].

Despite the lack of clear evidence for this clinical practice, one recent review estimated that 11% of adult outpatients with ARTIs across the US were treated with oral steroids and 23% in the state of Louisiana were treated with injectable steroids [11]. If true, such prescribing trends could be putting tens of thousands of patients at increased risk of adverse events without clear clinical benefits [1, 9, 10]. However, these prescribing rates were based on survey data, which may be subject to recall inaccuracy, particularly regarding details of medication use, such as dose and duration of the prescription [12]. Since these results were drawn from a limited number of years (2012–2013 for oral steroids and 2014 steroid injections), there was no ability to assess time trends. Finally, the data on injectable steroids were limited to one state, leaving open the question of whether there was any regional variation in clinical practice patterns.

To provide a more comprehensive assessment of US prescribing of systemic steroids for ARTI, we used a large claims database across 10 years with nationwide coverage and stratified patients by whether they received steroids associated with ARTI diagnosis via oral, intravenous, and intramuscular routes. We also sought to determine the regional differences and other predictors associated with this practice, since prior analyses have revealed that treating ARTI with steroid injections might be a common practice in the southern US [11].

## Methods

### Study design and data sources

This is a cohort study based on retrospective analysis of a large commercial health insurance database, IBM MarketScan, from January 1, 2007, to December 31, 2016. It contains deidentified records of more than 250 million patients, capturing longitudinal, individual-level administrative claims data from the US, including three components: the Commercial Claims and Encounters Database, the Medicare Supplemental and Coordination of Benefits Database, and the Medicaid Database. Data were drawn from large employers, health plans, and public organizations in the US, providing information on plan enrollment, healthcare utilization and expenditures, demographics, integrated records for inpatient and outpatient events (including diagnosis and procedure codes), and pharmacy dispensings. This database includes individuals with private health insurance coverage but not those uninsured or with public insurance as the primary payor. Electronic outpatient pharmacy dispensing records are considered accurate because pharmacists fill prescriptions with little room for interpretation and are reimbursed by insurers on the basis of detailed, complete, and accurate claims submitted electronically [13]. Pharmacy dispensing information is usually seen as the gold standard of drug exposure information compared to self-reported information [12] or prescribing records in outpatient medical records [14]. The Institutional Review Board of the Brigham and Women’s Hospital approved the study protocol and patient privacy precautions. This study is reported as per the REporting of studies Conducted using Observational Routinely-collected health Data (RECORD) guideline (S1 Checklist). All analysis plans and definitions were specified prior to study implementation. The definitions of the study variables were based on literature and validation studies [11, 15–18]. The study protocol is available as S1 Text in the supporting information online.

### Study population

The study population was derived from patients aged 18 or older with an ARTI diagnosis recorded in an ambulatory visit between January 1, 2007, and December 31, 2016, without the same diagnosis recorded in the preceding 180 days. Eligible ARTI diagnoses included acute bronchitis, sinusitis, pharyngitis, otitis media, allergic rhinitis, influenza, pneumonia, and unspecified acute upper respiratory infections. To avoid including injectable or oral steroids prescribed in the context of patients with severe arthritis, we excluded encounters associated with rheumatology or orthopedic services as well as those with diagnoses of noninfectious arthritis or spondylosis on the cohort entry date and the preceding 180 days. We excluded patients if they were in nursing home in the 180 days prior to the cohort entry date (drug exposure data not available for these institutionalized patients). To ensure we have sufficient data to assess baseline comorbidities, patients were required to have continuous insurance enrollment and drug benefit coverage during the 365 days prior to cohort entry date. Patients were excluded if aged 65 or older, owing to their eligibility for the federal Medicare program (IBM MarketScan only has Medicare Supplemental but not fee-for-service Medicare claims). In addition, we excluded patients who were prescribed systemic steroids or with the medical conditions in the 365 days prior to the cohort entry date for which systemic steroids may be appropriate. These conditions include asthma, chronic obstructive pulmonary disease, inflammatory bowel disease, malignant neoplasm, organ transplant, interstitial lung disease, urticaria, rheumatoid arthritis, systemic lupus erythematosus, and systemic vasculitis (see S1 Text for definitions of these conditions).

### Patient characteristics

We extracted and adjusted for the following covariates: age, sex, ARTI indications, geographical region, provider type (nurse practitioner, physician assistants, general medicine physicians [internists or family medicine doctors], medical specialists, or otolaryngology [ENT] doctors versus emergency department [ED] physicians), care location (regular office, urgent care, or walk-in retail clinic versus ED), employment status, insurance plan, related prescription drug use (nonsteroidal anti-inflammatory drugs, proton-pump inhibitors, histamine-2-receptor antagonists, antibiotics, antiplatelets, anticoagulants), and multiple comorbidities, including diabetes mellitus, hypertension, stroke, kidney dysfunction, dementia, obesity, heart failure, ischemic heart disease, atrial fibrillation, venous thromboembolism, urinary tract infections, human immunodeficiency virus (HIV) infection/acquired immune deficiency syndrome (AIDS), fractures, prior falls, gastroesophageal reflux disease (GERD), peptic ulcer disease, major bleeding events, bronchiectasis, connective tissue diseases, and remote history of nonseptic arthritis or spondyloarthropathy and a combined comorbidity score [19] (see S1 Text for definitions of these conditions). The baseline assessment period was the 365 days prior to the cohort entry date.

### Study outcomes

The primary outcome was defined as having a dispensing record or a procedure code indicating use of systemic steroids orally and parenterally (including intravenous, intramuscular, and nonspecific injectable forms) within 7 days of cohort entry date. See S1 Text for details of the study outcome definitions. Follow-up began on the cohort entry date and continued until first of occurrence of outcome event, disenrollment from the insurance or drug coverage plan, death, hospitalization, or nursing home admission (IBM MarketScan only has outpatient dispending records that do not capture medication use in the hospital or skilled nursing facility) or 7 days after cohort entry date.

### Primary and secondary analysis

The study outcome was measured as proportion per 100 patients. The association between patient characteristics and the outcome was assessed by univariate and multivariate logistic regression. Secondary analyses were conducted to test robustness of our findings. First, we repeated all our analyses after restricting to use of systemic steroids within 3 days of cohort entry. Second, we excluded patients with nasal polyps, since in the field of ENT, systemic steroids are commonly prescribed to treat sinusitis [20], particularly sinusitis with polyposis [9]. All analyses were conducted using the Aetion platform and R, version 3.1.2.5 (R Foundation for Statistical Computing), which has been previously validated for use in observational studies [21, 22] and for predicting clinical trial findings [23].

## Results

**Fig 1:**
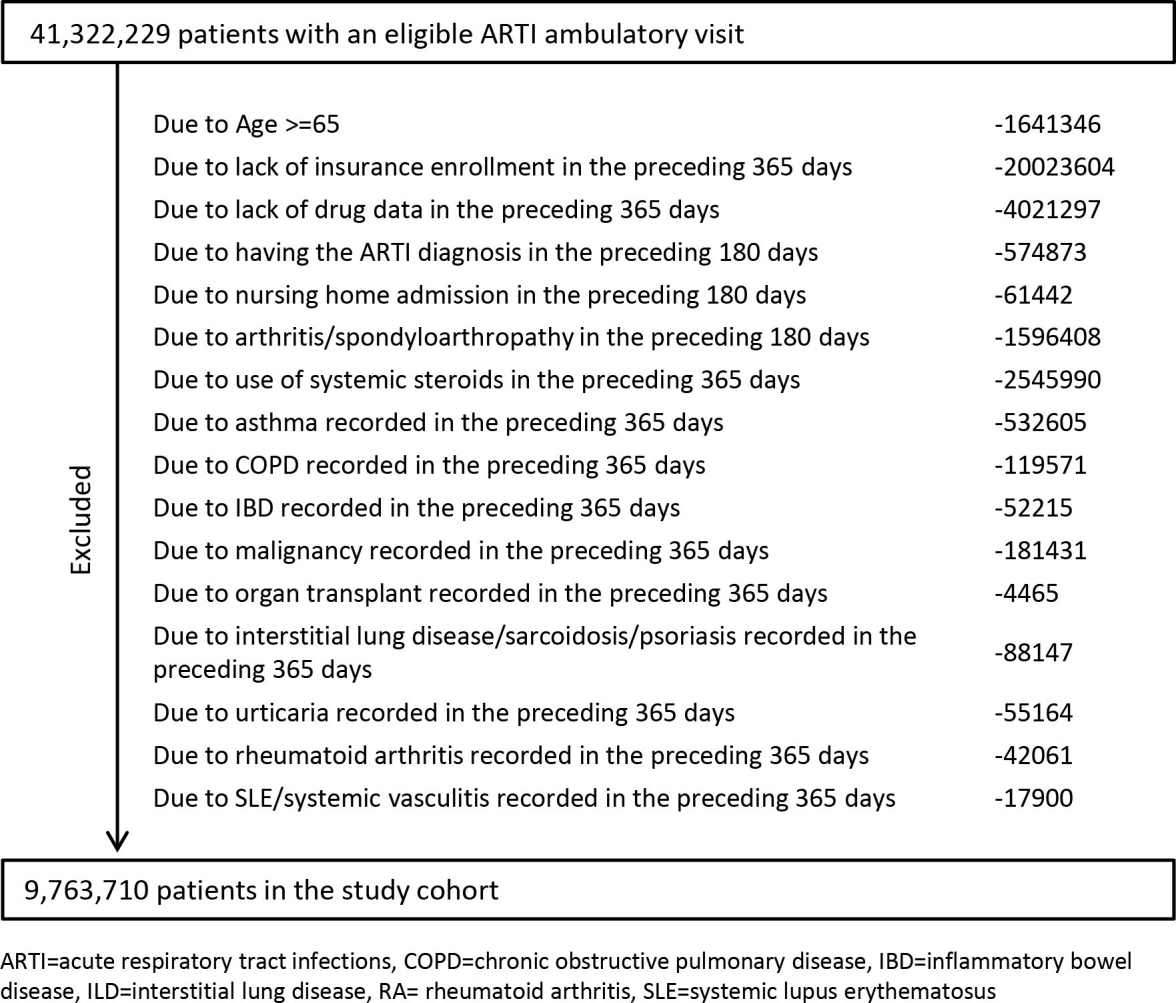
Study attrition chart.

A total of 41,322,229 patients with ambulatory encounters had one of the eligible ARTI diagnoses, and our study population consisted of 9,763,710 patients (mean age 39.6 years, female 56.0%, Fig 1). With a mean follow-up of 6.35 days (standard deviation [SD] = 1.7 days), 11.8% of patients with an ARTI-related ambulatory visit were prescribed systemic steroids (46.1% parenteral only, 47.3% oral only, 6.6% using both routes; Table 1). Among systemic steroid users with detailed medication information available, 45.5% were prescribed prednisone equivalents of <20 mg, 23.5% 20–39 mg, and 30.9% ≥40 mg. Most (84.7%) were prescribed a steroid prescription of 7 days or fewer, 14.8% 8–29 days, and 0.5% ≥30 days (Table 1).

**Table 1:** Steroid use for ARTIs.

	Steroid use within 7 days	Steroid use within 3 days
**Total number of patients**	9,763,710	9,763,710
**Systemic steroids use, *N* patients**	1,154,378	1,092,626
**Prescribing rate, % (95% confidence interval)**	11.82 (11.80–11.84)	11.19 (11.17–11.21)
**Route of administration**		
Parenteral routes alone, *N* (%)	532,142 (46.1)	524,987 (48.0)
Oral route alone, *N* (%)	545,487 (47.3)	501,601 (45.9)
Oral + parenteral, *N* (%)	76,749 (6.6)	66,038 (6.0)
Daily dose in prednisone equivalents, *N* (%)*		
<20 mg	280,704 (45.5)	255,971 (45.6)
20–39 mg	144,875 (23.5)	131,962 (23.5)
≥40 mg	190,739 (30.9)	173,995 (31.0)
Length of supply dispensed, *N* (%)*		
≤7 days	522,192 (84.7)	479,467 (85.3)
8–29 days	91,137 (14.8)	79,964 (14.2)
≥30 days	2,989 (0.5)	2,497 (0.4)

*Percent among patients with “dose” and “length of supply dispensed” information.

Abbreviation: ARTI, acute respiratory tract infection

### Systemic steroid use by ARTI indication

Among patients with an ARTI diagnosis in an outpatient setting, those diagnosed with acute bronchitis was associated with the highest odds of receiving systemic steroid (adjusted odds ratio [aOR] 2.70, 95% confidence interval [CI] 2.68–2.72, *p* < 0.001), followed by acute sinusitis (aOR 2.03, 95% CI 2.02–2.04, *p* < 0.001), pneumonia (aOR 1.80, 95% CI 1.77–1.82, *p* < 0.001), allergic rhinitis (aOR 1.74, 95% CI 1.72–1.75, *p* < 0.001), otitis media (aOR 1.43, 95% CI 1.42–1.45, *p* < 0.001), pharyngitis (aOR 1.42, 95% CI 1.41–1.43, *p* < 0.001), and acute upper respiratory infections (aOR 1.23, 95% CI 1.22–1.24, *p* < 0.001). Among those with an ARTI diagnosis, having a diagnosis of influenza was associated with lower odds of receiving systemic steroids (aOR 0.65, 95% CI 0.64–0.66, *p* < 0.001; Table 2).

**Table 2:** Patient characteristics and association with use of systemic corticosteroids.

Characteristic	Total population, *N* = 9,763,710, *N*	Receiving steroids*, *N* = 1,154,378, *N* (%)	Univariate OR	Multivariate aOR**
Age categories				
18 to <25	1,752,290	184,968 (10.6%)	Ref	Ref
25 to <35	1,975,722	222,489 (11.3%)	1.08 (1.07–1.08)	1.07 (1.06–1.08)
35 to <45	2,231,358	267,660 (12.0%)	1.16 (1.15–1.16)	1.17 (1.16–1.18)
45 to <55	2,167,879	275,439 (12.7%)	1.23 (1.23–1.24)	1.25 (1.24–1.26)
≥55	1,636,461	203,822 (12.5%)	1.21 (1.20–1.21)	1.22 (1.21–1.23)
Sex				
Male	4,297,795	540,236 (12.6%)	Ref	Ref
Female	5,465,915	614,142 (11.2%)	0.88 (0.88–0.88)	0.87 (0.87–0.88)
ARTI indication				
Unspecified upper respiratory infections	2,354,975	223,990 (9.5%)	0.73 (0.73–0.74)	1.23 (1.22–1.24)
Otitis media	248,978	30,179 (12.1%)	1.03 (1.02–1.04)	1.43 (1.42–1.45)
Sinusitis	2,398,502	359,649 (15.0%)	1.46 (1.45–1.46)	2.03 (2.02–2.04)
Pharyngitis	2,272,142	243,711 (10.7%)	0.87 (0.86–0.87)	1.42 (1.41–1.43)
Allergic rhinitis	1,498,960	196,792 (13.1%)	1.15 (1.15–1.16)	1.74 (1.73–1.75)
Acute bronchitis	1,442,379	260,681 (18.1%)	1.83 (1.82–1.84)	2.70 (2.68–2.72)
Pneumonia	235,440	28,307 (12.0%)	1.02 (1.01–1.03)	1.80 (1.77–1.82)
Influenza	332,623	20,548 (6.2%)	0.48 (0.47–0.49)	0.65 (0.64–0.66)
Region				
Northeast	1,519,033	90,238 (5.9%)	Ref	Ref
North Central	2,231,576	177,785 (8.0%)	1.37 (1.36–1.38)	1.41 (1.40–1.42)
South	4,120,036	768,350 (18.6%)	3.63 (3.60–3.66)	3.78 (3.75–3.81)
West	1,740,782	103,579 (6.0%)	1.00 (0.99–1.01)	1.08 (1.07–1.09)
Unknown	152,283	14,426 (9.5%)	1.66 (1.63–1.69)	1.69 (1.66–1.73)
Provider type				
General medicine	8,232,102	945,074 (11.5%)	Ref	Ref
Medical specialist	214,225	16,995 (7.9%)	0.66 (0.65–0.67)	0.68 (0.67–0.69)
ED physician	559,897	82,476 (14.7%)	1.33 (1.32–1.34)	1.16 (1.15–1.17)
ENT physician	218,938	37,103 (16.9%)	1.57 (1.56–1.59)	1.48 (1.46–1.50)
Nurse practitioner	372,791	53,333 (14.3%)	1.29 (1.28–1.30)	1.10 (1.08–1.11)
Physician assistant	164,030	19,308 (11.8%)	1.03 (1.01–1.04)	1.10 (1.08–1.12)
Care location				
Regular office visit	9,034,253	1,048,476 (11.6%)	Ref	Ref
Urgent care	328,244	48,388 (14.7%)	1.32 (1.30–1.33)	1.27 (1.26–1.28)
Walk-in retail clinic	7,467	694 (9.3%)	0.78 (0.72–0.84)	0.65 (0.60–0.70)
Emergency room	393,746	56,820 (14.4%)	1.28 (1.27–1.30)	1.19 (1.18–1.21)
DM	560,839	54,566 (9.7%)	0.79 (0.79–0.80)	0.68 (0.68–0.69)
HTN	1,440,025	188,226 (13.1%)	1.15 (1.14–1.15)	1.12 (1.11–1.13)
Stroke	48,340	5,651 (11.7%)	0.99 (0.96–1.02)	0.97 (0.94–1.00)
Kidney dysfunction	40,425	4,064 (10.1%)	0.83 (0.81–0.86)	0.93 (0.89–0.96)
Liver disease	129,804	14,256 (11.0%)	0.92 (0.90–0.94)	0.96 (0.94–0.98)
Dementia	11,307	1,155 (10.2%)	0.85 (0.80–0.90)	0.85 (0.80–0.91)
Obesity	312,085	39,220 (12.6%)	1.07 (1.06–1.09)	1.00 (0.99–1.01)
Heart failure	30,396	3,259 (10.7%)	0.90 (0.86–0.93)	1.00 (0.95–1.04)
Ischemic heart disease	179,551	23,113 (12.9%)	1.10 (1.09–1.12)	1.02 (1.00–1.04)
Atrial fibrillation	46,100	4,987 (10.8%)	0.90 (0.88–0.93)	0.98 (0.95–1.01)
VTE	16,022	1,587 (9.9%)	0.82 (0.78–0.86)	0.93 (0.88–0.99)
Urinary tract infections	571,447	63,957 (11.2%)	0.94 (0.93–0.94)	0.94 (0.93–0.95)
HIV/AIDS	16,785	1,360 (8.1%)	0.66 (0.62–0.70)	0.62 (0.59–0.66)
Fractures	118,412	13,852 (11.7%)	0.99 (0.97–1.01)	1.02 (1.01–1.04)
Falls	29,154	3,661 (12.6%)	1.07 (1.03–1.11)	1.03 (1.00–1.07)
GERD	353,109	44,529 (12.6%)	1.08 (1.08–1.09)	0.93 (0.92–0.94)
Peptic ulcer disease	19,689	2,446 (12.4%)	1.06 (1.01–1.10)	0.99 (0.95–1.04)
Major bleeding events	12,886	1,238 (9.6%)	0.79 (0.75–0.84)	0.86 (0.81–0.91)
Bronchiectasis	1,382	109 (7.9%)	0.64 (0.53–0.78)	0.70 (0.57–0.85)
Connective tissue diseases	6,245	744 (11.9%)	1.01 (0.93–1.09)	0.93 (0.86–1.01)
Use of NSAIDs	1,177,851	151,542 (12.9%)	1.12 (1.11–1.12)	1.09 (1.08–1.10)
Use of PPIs	672,651	89,762 (13.3%)	1.16 (1.15–1.17)	1.14 (1.13–1.15)
Use of H2RAs	94,435	10,486 (11.1%)	0.93 (0.91–0.95)	0.98 (0.96–1.01)
Use of antibiotics	2,930,232	360,876 (12.3%)	1.07 (1.06–1.07)	1.10 (1.09–1.10)
Use of antiplatelets	90,369	12,160 (13.5%)	1.16 (1.14–1.18)	1.08 (1.06–1.10)
Use of anticoagulants	52,308	5,354 (10.2%)	0.85 (0.83–0.87)	0.88 (0.85–0.91)
Combined comorbidity score category				
<1	7,625,943	896,204 (11.8%)	Ref	Ref
1–2	1,707,347	209,643 (12.3%)	1.05 (1.05–1.06)	0.91 (0.90–0.92)
2–4	380,088	43,694 (11.5%)	0.98 (0.97–0.99)	0.85 (0.84–0.87)
≥4	50,332	4,837 (9.6%)	0.80 (0.78–0.82)	0.71 (0.69–0.74)
Employment status				
Active full time	5,508,470	651,569 (11.8%)	Ref	Ref
Retiree	503,273	58,215 (11.6%)	0.97 (0.97–0.98)	0.98 (0.97–0.99)
Active part-time	104,025	10,259 (9.9%)	0.82 (0.80–0.83)	0.91 (0.89–0.93)
Unknown/other	3,647,942	434,335 (11.9%)	1.01 (1.00–1.01)	0.99 (0.99–1.00)
Insurance plan type				
PPO	5,960,060	748,413 (12.6%)	Ref	Ref
Comprehensive	179,535	20,230 (11.3%)	0.88 (0.87–0.90)	1.07 (1.05–1.09)
EPO	129,760	11,460 (8.8%)	0.68 (0.66–0.69)	0.85 (0.83–0.87)
HMO	1,431,532	116,018 (8.1%)	0.61 (0.61–0.62)	0.72 (0.71–0.72)
POS	803,251	110,638 (13.8%)	1.11 (1.10–1.12)	1.14 (1.13–1.14)
CDHP	550,856	73,079 (13.3%)	1.07 (1.06–1.07)	0.94 (0.93–0.94)
HDHP	341,572	37,251 (10.9%)	0.85 (0.84–0.86)	0.82 (0.81–0.83)
Others/missing	367,144	37,289 (10.2%)	0.79 (0.78, 0.80)	1.00 (0.99–1.01)
Year of cohort entry date				
2007	1,368,068	143,446 (10.5%)	Ref	Ref
2008	1,194,950	125,948 (10.5%)	1.01 (1.00–1.01)	1.09 (1.08–1.10)
2009	1,292,822	124,794 (9.7%)	0.91 (0.90–0.92)	1.04 (1.03–1.05)
2010	991,226	111,076 (11.2%)	1.08 (1.07–1.09)	1.25 (1.24–1.26)
2011	1,040,685	110,962 (10.7%)	1.02 (1.01–1.03)	1.20 (1.19–1.21)
2012	1,104,266	144,628 (13.1%)	1.29 (1.28–1.30)	1.44 (1.43–1.45)
2013	837,644	103,709 (12.4%)	1.21 (1.20–1.22)	1.48 (1.47–1.50)
2014	758,101	100,744 (13.3%)	1.31 (1.30–1.32)	1.57 (1.56–1.58)
2015	576,617	89,806 (15.6%)	1.57 (1.56–1.59)	1.76 (1.75–1.78)
2016	599,331	99,265 (16.6%)	1.69 (1.68–1.71)	1.93 (1.91–1.94)

*Within 7 days of an ARTI.

**Adjusted for all the variables listed in Table 2.

Abbreviations: aOR, adjusted odds ratio; ARTI, acute respiratory tract infections; CDHP, consumer-driven health plan; COBRA, Consolidated Omnibus Budget Reconciliation Act; DM, diabetes mellitus; ED, emergency department; ENT, otolaryngology; EPO, exclusive provider organization; GERD, gastroesophageal reflux disease; H2RA, histamine-2-receptor antagonist; HDHP, high-deductible health plan; HIV/AIDS, human immunodeficiency virus/acquired immune deficiency syndrome; HMO, health maintenance organization; HTN, hypertension; NSAID, nonsteroidal anti-inflammatory drug; POS, point of service; PPI, proton-pump inhibitor; PPO, preferred provider organization; VTE, venous thromboembolism

### Geographical variability

**Fig 2:**
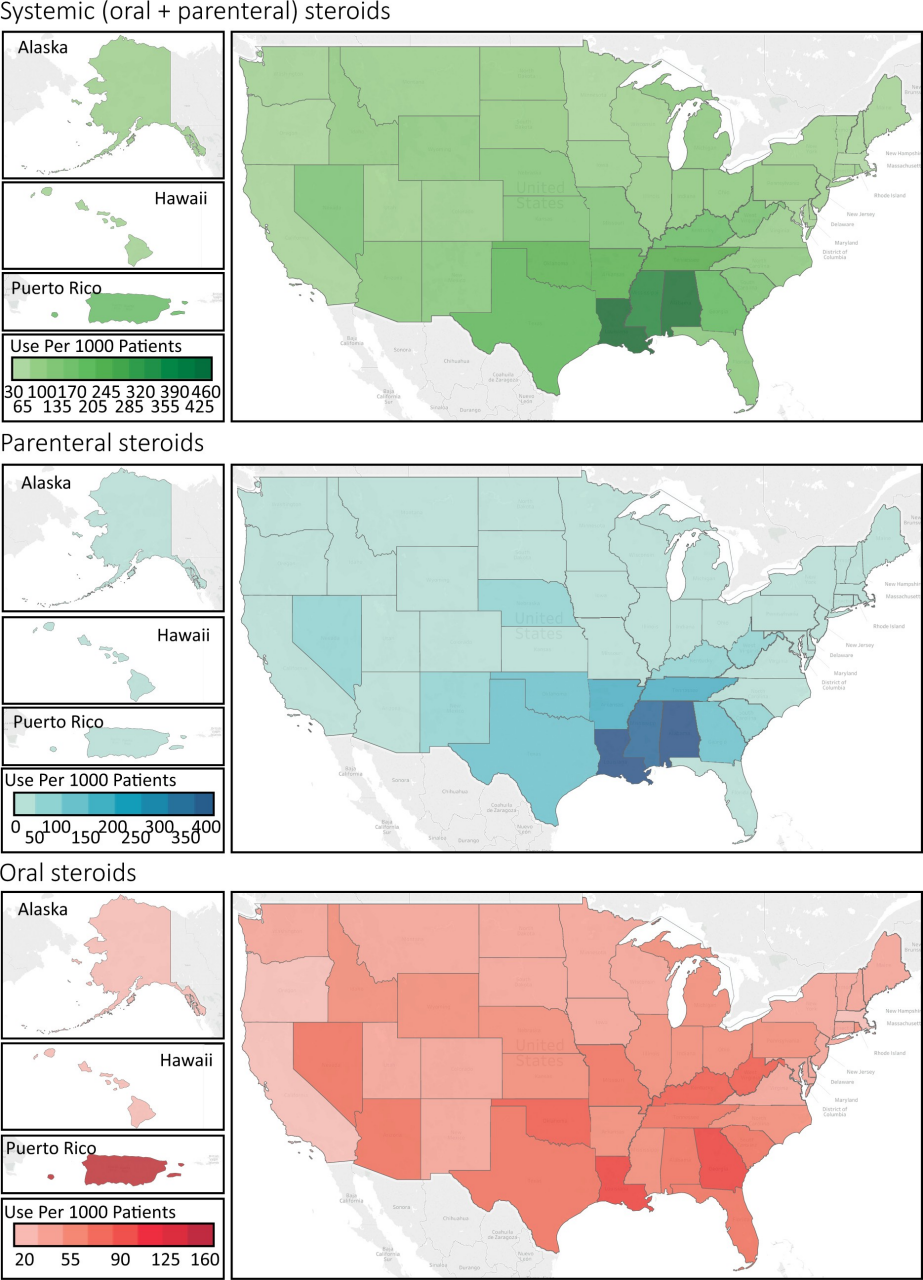
Geographical variability in prescribing systemic steroids for acute respiratory tract infections. Source of the base map: https://www.census.gov/geographies/mapping-files/2017/geo/kml-cartographic-boundary-files.html.

We found remarkable regional differences. Patients seeking care for ARTI in the South were 3.78 times (95% CI 3.75–3.81, *p* < 0.001) more likely to be prescribed a systemic steroid than those cared in the Northeast. Such prescribing differences were more pronounced for parenteral steroids than for oral steroids. The aOR of receiving parenteral steroids was 14.48 (95% CI 14.23–14.72, *p* < 0.001) comparing the South versus Northeast, and the corresponding aOR was 1.68 (95% CI 1.66–1.69, *p* < 0.001) for oral steroids (Fig 2 and Table 3).

**Table 3:** Associations between geographical region and use of systemic steroids.

Variable	aOR (95% CI)
**Any steroids**	** **
North Central versus Northeast	1.41 (1.40–1.42)
South versus Northeast	3.78 (3.75–3.81)
West versus Northeast	1.08 (1.07–1.09)
Unknown versus Northeast	1.69 (1.66–1.73)
**Parenteral steroids (IV or IM)**	** **
North Central versus Northeast	2.61 (2.57–2.66)
South versus Northeast	14.48 (14.23–14.72)
West versus Northeast	2.38 (2.33–2.43)
Unknown versus Northeast	3.74 (3.62–3.87)
**Oral steroids**	** **
North Central versus Northeast	1.22 (1.21–1.23)
South versus Northeast	1.68 (1.66–1.69)
West versus Northeast	0.84 (0.83–0.85)
Unknown versus Northeast	1.36 (1.33–1.39)

Adjusted for all the variables listed in Table 2.

Abbreviations: aOR, adjusted odds ratio; CI, confidence interval; IM, intramuscular; IV, intravenous.

### Provider type and care location

Compared to general medicine physicians, ENT specialists were associated with the highest prescribing rate of systemic steroids for ARTI (aOR 1.48, 95% CI 1.46–1.50, *p* < 0.001), followed by ED physicians (aOR 1.16, 95% CI 1.15–1.17, *p* < 0.001), physician assistants (aOR 1.10, 95% CI 1.08–1.12, *p* < 0.001), nurse practitioners (aOR 1.10, 95% CI 1.08–1.11, *p* < 0.001), and medical specialists, who had a lower rate (aOR 0.68, 95% CI 0.67–0.69, *p* < 0.001; Table 2). Compared to ambulatory care office visits, systemic steroids were more likely to be prescribed to treat ARTIs in urgent care (aOR 1.27, 95% CI 1.26–1.28, *p* < 0.001), followed by ED (aOR 1.19, 95% CI 1.18–1.21, *p* < 0.001), and they are less likely to be prescribed in walk-in retail clinics (aOR 0.65, 95% CI 0.60–0.70, *p* < 0.001; Table 2).

### Comorbidities

Individual comorbidities negatively associated with odds of systemic steroid prescribing were diabetes, kidney dysfunction, liver disease, dementia, venous thromboembolism, major bleeding events, GERD, bronchiectasis, urinary tract infections, HIV/AIDS, and bronchiectasis. Using a validated combined comorbidity score [19], patients with more comorbidities were associated with a lower odds of receiving systemic steroids. Use of antibiotics, proton-pump inhibitors, antiplatelets, and nonsteroidal anti-inflammatory drugs were positively associated with odds of systemic steroid prescribing (Table 2).

### Time trend

**Fig 3:**
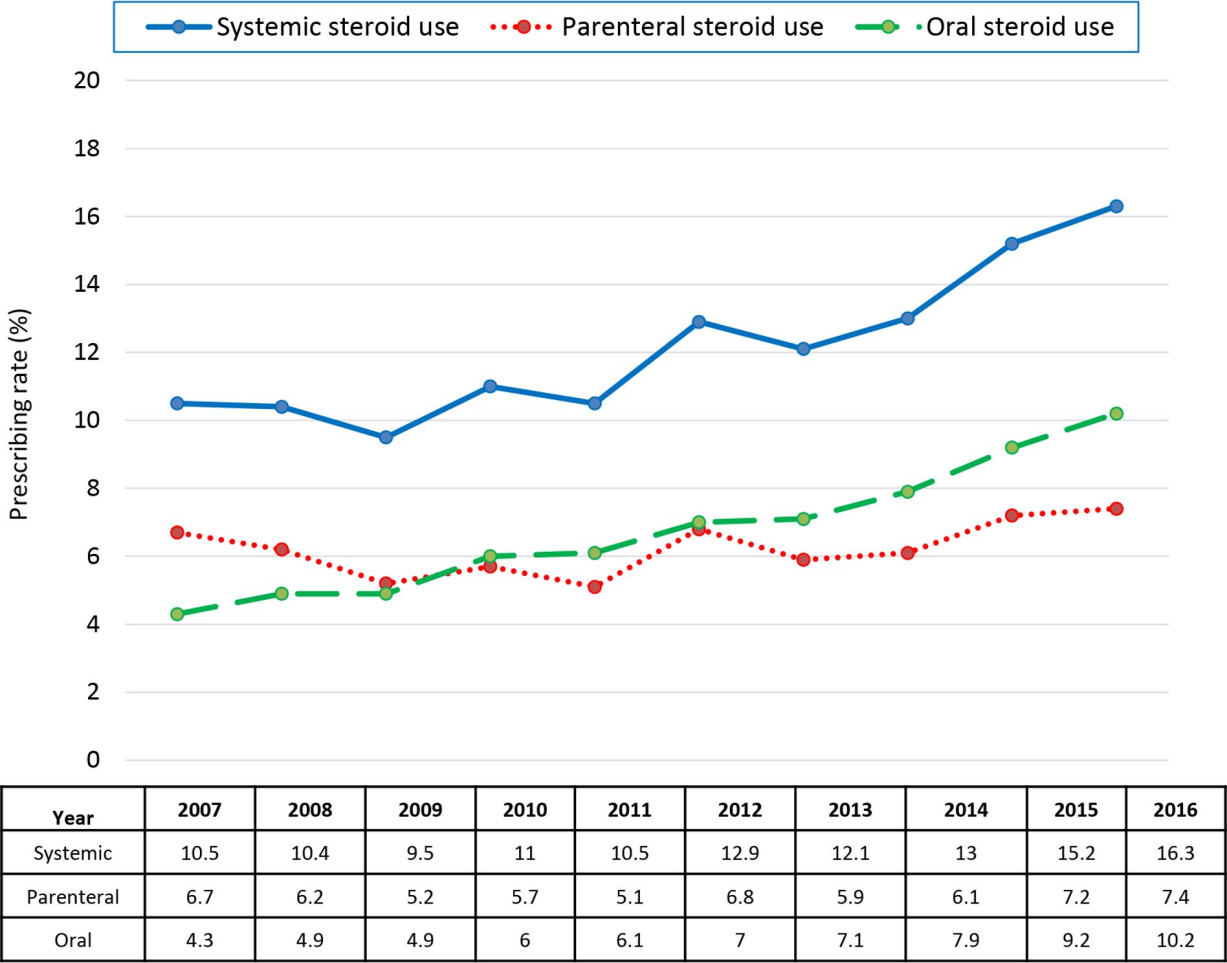
Time trend of prescribing rates of systemic steroids for acute respiratory tract infections.

Prescribing of systemic, parenteral, and oral steroids for ARTI all increased from 2007 to 2016 (*p* < 0.001 for all three, Fig 3). The prescribing rate for systemic steroids in 2016 was almost double that of 2007 (aOR 1.93, 95% CI 1.91–1.94). The corresponding aOR was 1.33 (95% CI 1.31–1.35) for parenteral steroids and 2.74 (95% CI 2.71–2.78) for oral steroids (Fig 3).

### Sensitivity analysis

When restricting to systemic steroid use within the first 3 days of ARTI diagnosis, the prescribing patterns were similar to that for steroid use in 7 days of ARTI diagnosis (Table 1), and the results of all analyses were similar (see S1 and S2 Tables). After excluding patients with nasal polyps, our estimates for all analyses were not materially changed, and ENT physicians were still associated with an aOR of prescribing systemic steroids of 1.47 (95% CI 1.45–1.48) when compared to the general practitioners.

## Discussion

Using a national sample of privately insured US patients over the last decade, we found 11.8% of patient encounters with ARTI resulted in receiving systemic steroid treatments. Such prescribing has almost doubled from 2007 to 2016, with patients far more likely to receive this care—particularly injectable steroids—in the southern US, even though use of systemic steroid treatments for ARTIs lacks clear scientific justification. Providers in the ED and urgent care, as well as ENT specialists, were more likely to be prescribers. Use of steroids for ARTIs has been increasing over time, with as many as 16.3% of US patients with an ARTI diagnosis aged 18–64 years—or 10.6 million people—receiving such treatment in 2016.

Our estimates of systemic steroid use for ARTI were consistent with a prior study that used national survey data and local administrative data [11]. We further quantified such prescribing by route of administration and found a disproportionally high prescribing rates in the southern states than in other states, especially for the parenteral routes [11]. We did not find any meaningful differences in patient demographics, ARTI indications, care settings, provider types, and patient comorbidities that can explain the remarkable geographical variability in steroid use for ARTI (S3 Table). To put geographical variations of patient characteristics into perspective, the observed risk ratio of 3.78 associated with the southern region could only be fully explained by an unmeasured confounder that was associated with both the southern region and steroid prescribing by a risk ratio of 7.02-fold each [24], above and beyond the adjusted factors. By the same formula, only an unmeasured confounder associated with both the southern region and steroid prescribing by a risk ratio of 28.45-fold each could account for the 14-fold increased odds of receiving parenteral steroids in the South [24]. The aORs comparing the southern region to all other regions were far smaller than these required thresholds (S3 Table). Given lack of convincing evidence to guide such practice, it is not surprising we did not identify any objective factors associated with it. The regional difference therefore are most likely related to local culture, physician preferences, and patient expectations. There were some similarities of the regional differences in the use of systemic steroids versus antibiotics for ARTI, in which the highest prescribing rates of both systemic steroids and antibiotics for ARTI were observed in the southern states [25]. Since both practices are potentially inappropriate, future research is warranted to investigate the possible impact and interactions of the two practices on clinical outcomes by region.

There was very little evidence supporting prescribing systemic steroids for ARTI. As for oral steroids, data from RCTs have shown that treating pharyngitis with systemic steroids may shorten time to resolution of sore throat [7]. For acute sinusitis, meta-analysis of RCTs has deemed systemic steroids to be ineffective as monotherapy, and the small benefit in symptom relief when used as an adjuvant therapy with antibiotics could possibly be explained by attrition bias [8]. An RCT also revealed that systemic steroids are ineffective in the treatment of lower respiratory tract infections [4]. All the prior RCTs investigating systemic steroid use in community-acquired pneumonia recruited hospitalized patients; among them, steroids as adjuvant therapy to proper antibiotics were shown to reduce mortality and morbidity only in patients with severe pneumonia but not for those with nonsevere pneumonia, casting doubt on generalizing the effectiveness to the ambulatory settings [26]. There were very limited RCT data in steroid use in common cold (only intranasal steroids were studied, which was shown to be ineffective [5]) and otitis media (only pediatric population was studied, which was found to be ineffective [6]). With questionable benefits and substantial risks [9, 10], treating ARTI with systemic steroids has not been recommended by clinical guidelines [1].

Systemic steroid prescribing rates were the highest in urgent care or ED. Because the only demonstrated benefits associated with systemic steroid use in ARTI is symptomatic relief, it is possible people with more severe symptoms seek medical attention in urgent care or ED settings, leading to steroid prescriptions. We found that people with more complex comorbidities are less likely to receive systemic steroids for ARTI. It is plausible that providers are less inclined to prescribe steroids to these vulnerable populations who are more susceptible to developing serious side effects from systemic steroid use [27, 28]. However, adjusting for individual comorbidities, older age was predictive of more steroid prescribing in ARTI. As older age is a strong risk factor for steroid-related complications, including gastrointestinal bleeding [29], sepsis [30], venous thromboembolism [31], and osteoporotic fracture [32], these findings convey an urgent need to reduce this potentially harmful practice.

We also found a steadily increasing trend in prescribing systemic steroids from 2007 to 2016 that was more pronounced for oral than parenteral steroids. The studies showing systemic steroids can lead to faster symptom relief in some limited ARTI indications [7–9] may have encouraged use over time for patients with more severe symptoms. Other factors contributing to this trend could include low prices for steroid prescriptions—for example, numerous oral steroids are often available on $4 generic lists that pharmacies started promoting about a decade ago [33]—and payers’ increasingly choosing to integrate patient quality ratings into provider reimbursement. Surveys show that patients often feel better about their physician visits when that visit results in a prescription or other interventions [34], although in this case, the prescriptions do not have supporting evidence behind them.

Our results call for an effective medical education program to help disseminate the messages about the potential risks and limited benefits of steroid prescribing in the context of ARTIs, faithfully reflecting totality of the existing evidence. For example, both physicians and patients should be well informed that treating pharyngitis with systemic steroids may shorten time to resolution of sore throat by about 11 hours [7], at the cost of some potentially serious side effects, including gastrointestinal disturbances, insomnia, sepsis, venous thromboembolism, and fracture, which can occur as early as the first 30 days after a short-term use [9, 10]. Investing in medical education programs to help transform clinical practice in this area would improve patient outcomes and reduce health system spending on managing the side effects of such non-evidence-driven care.

Our study has some limitations. First, our primary outcome was systemic steroid prescription within 7 days of ARTI diagnosis, and the indication was not written directly on the prescription or dispensing record. It is possible that some of the prescriptions were not intended to treat ARTI. To minimize erroneous association with the steroid use, we excluded patients with an extensive list of medical conditions for which systemic steroid use may be appropriate as well as those exposed to systemic steroids in the prior year. Also, our sensitivity analysis assessing steroid use within 3 days of ARTI diagnosis showed very similar results (Table 1). Second, we could not stratify our analysis by severity of symptoms because such information was not available in the IBM MarketScan database. However, this limitation should not affect the implication of our findings, as evidence suggests prescribing systemic steroids for ARTI may not be associated with a favorable risk-to-benefit ratio regardless of symptom severity [9, 10]. Third, since we excluded a wide range of steroid-indicated conditions, including asthma, chronic obstructive pulmonary disease, malignancy, and many allergic and autoimmune diseases, our findings cannot be generalized to patients with these conditions as comorbidities. The definitions of the conditions used for inclusion/exclusion criteria were based on prior literature and validation studies [11, 15–18]. As none of these algorithms is perfect, misclassification of our study variables is possible. However, given that we excluded those who received systemic steroids in the year prior to the cohort entry, our estimated prescribing rates for ARTI is probably conservative. Lastly, our findings are based on patients aged between 18 and 64 years with commercial medical insurance and may not be generalizable to older populations or patients with public health insurance coverage.

Despite these limitations, we found 11.8% of ARTI encounters results in patients being treated with systemic steroids. Such prescribing has been steadily increasing from 2007 to 2016 and is far more common in the southern US. These findings call for an effective medical education program to reduce this practice, which lacks clear scientific justification.

## Supporting information

S1 Checklist RECORD Checklist. RECORD, REporting of studies Conducted using Observational Routinely-collected health Data.
(DOCX)

S1 Text Study protocol and definitions of the study variables. (DOCX)

S1 Table Patient characteristics and association with use of systemic corticosteroids within 3 days of an outpatient diagnosis of acute respiratory tract infections. (DOCX)

S2 Table Associations between geographical region and use of systemic steroids within 3 days of an acute respiratory tract infection diagnosis. (DOCX)

S3 Table Patient characteristics by geographical region. (DOCX)

## Abbreviations

AIDS: acquired immune deficiency syndrome
aOR: adjusted odds ratio
ARTI: acute respiratory tract infection
CDHP: consumer-driven health plan
CI: confidence interval
COBRA: Consolidated Omnibus Budget Reconciliation Act
ED: emergency department
ENT: otolaryngology
EPO: exclusive provider organization
GERD: gastroesophageal reflux disease
HDHP: high-deductible health plan
HIV: human immunodeficiency virus
HMO: health maintenance organization
NSAID: nonsteroidal anti-inflammatory drug
POS: point of service
PPO: preferred provider organization
RCT: randomized control trial
RECORD: REporting of studies Conducted using Observational Routinely-collected health Data
SD: standard deviation

## References

[1] Adult Treatment Recommendations. CDC. 2017 [cited 2019 Jan 4]. Available from: https://www.cdc.gov/antibiotic-use/community/for-hcp/outpatient-hcp/adult-treatment-rec.html.

[2] Albert RH. Diagnosis and treatment of acute bronchitis. Am Fam Physician. 2010;82(11):1345–50. Epub 2010/12/03. . 21121518

[3] Fashner J, Ericson K, Werner S. Treatment of the common cold in children and adults. Am Fam Physician. 2012;86(2):153–9. Epub 2012/09/12. . 22962927

[4] Hay AD, Little P, Harnden A, Thompson M, Wang K, Kendrick D, et al Effect of Oral Prednisolone on Symptom Duration and Severity in Nonasthmatic Adults With Acute Lower Respiratory Tract Infection: A Randomized Clinical Trial. JAMA. 2017;318(8):721–30. Epub 2017/08/23. 10.1001/jama.2017.10572 28829884 PMC5817483

[5] Hayward G, Thompson MJ, Perera R, Del Mar CB, Glasziou PP, Heneghan CJ. Corticosteroids for the common cold. Cochrane Database Syst Rev. 2015;(10):CD008116 Epub 2015/10/16. 10.1002/14651858.CD008116.pub3 . 26461493 PMC8734596

[6] Francis NA, Cannings-John R, Waldron CA, Thomas-Jones E, Winfield T, Shepherd V, et al Oral steroids for resolution of otitis media with effusion in children (OSTRICH): a double-blinded, placebo-controlled randomised trial. Lancet. 2018;392(10147):557–68. Epub 2018/08/29. 10.1016/S0140-6736(18)31490-9 30152390 PMC6099122

[7] Sadeghirad B, Siemieniuk RAC, Brignardello-Petersen R, Papola D, Lytvyn L, Vandvik PO, et al Corticosteroids for treatment of sore throat: systematic review and meta-analysis of randomised trials. BMJ. 2017;358:j3887 Epub 2017/09/22. 10.1136/bmj.j3887 28931508 PMC5605780

[8] Venekamp RP, Thompson MJ, Hayward G, Heneghan CJ, Del Mar CB, Perera R, et al Systemic corticosteroids for acute sinusitis. Cochrane Database Syst Rev. 2014;(3):CD008115 Epub 2014/03/26. 10.1002/14651858.CD008115.pub3 . 24664368 PMC11179165

[9] Head K, Chong LY, Hopkins C, Philpott C, Burton MJ, Schilder AG. Short-course oral steroids alone for chronic rhinosinusitis. Cochrane Database Syst Rev. 2016;4:CD011991. Epub 2016/04/27. 10.1002/14651858.CD011991.pub2 . 27113367 PMC8504433

[10] Waljee AK, Rogers MA, Lin P, Singal AG, Stein JD, Marks RM, et al Short term use of oral corticosteroids and related harms among adults in the United States: population based cohort study. BMJ. 2017;357:j1415 Epub 2017/04/14. 10.1136/bmj.j1415 . 28404617 PMC6284230

[11] Dvorin EL, Lamb MC, Monlezun DJ, Boese AC, Bazzano LA, Price-Haywood EG. High Frequency of Systemic Corticosteroid Use for Acute Respiratory Tract Illnesses in Ambulatory Settings. JAMA Intern Med. 2018;178(6):852–4. Epub 2018/02/27. 10.1001/jamainternmed.2018.0103 29482204 PMC5885155

[12] West SL, Savitz DA, Koch G, Strom BL, Guess HA, Hartzema A. Recall accuracy for prescription medications: self-report compared with database information. Am J Epidemiol. 1995;142(10):1103–12. Epub 1995/11/15. 10.1093/oxfordjournals.aje.a117563 . 7485055

[13] Levy AR, O'Brien BJ, Sellors C, Grootendorst P, Willison D. Coding accuracy of administrative drug claims in the Ontario Drug Benefit database. Can J Clin Pharmacol. 2003;10(2):67–71. Epub 2003/07/25. . 12879144

[14] West SL, Strom BL, Freundlich B, Normand E, Koch G, Savitz DA. Completeness of prescription recording in outpatient medical records from a health maintenance organization. J Clin Epidemiol. 1994;47(2):165–71. Epub 1994/02/01. 10.1016/0895-4356(94)90021-3 . 8113825

[15] Tamariz L, Harkins T, Nair V. A systematic review of validated methods for identifying venous thromboembolism using administrative and claims data. Pharmacoepidemiol Drug Saf. 2012;21 Suppl 1:154–62. Epub 2012/01/25. 10.1002/pds.2341 . 22262602

[16] Patorno E, Pawar A, Franklin JM, Najafzadeh M, Deruaz-Luyet A, Brodovicz KG, et al Empagliflozin and the Risk of Heart Failure Hospitalization in Routine Clinical Care. Circulation. 2019;139(25):2822–30. Epub 2019/04/09. 10.1161/CIRCULATIONAHA.118.039177 30955357 PMC6594384

[17] Selby J, Reichman ME, Graham D, Butler M, Hampp C, Levenson M, Southworth MR, Toh D, Fireman B. MINI-SENTINEL MEDICAL PRODUCT ASSESSMENT: A PROTOCOL FOR ACTIVE SURVEILLANCE OF ACUTE MYOCARDIAL INFARCTION IN ASSOCIATION WITH USE OF ANTI-DIABETIC AGENTS. 2016 [cited 2019 Nov 19]. Available from: https://www.sentinelinitiative.org/sites/default/files/Drugs/Assessments/Mini-Sentinel_AMI-and-Anti-Diabetic-Agents_Protocol.pdf.

[18] Jafri K, Taylor L, Nezamzadeh M, Baker JF, Mehta NN, Bartels C, et al Management of hyperlipidemia among patients with rheumatoid arthritis in the primary care setting. BMC Musculoskelet Disord. 2015;16:237 Epub 2015/09/05. 10.1186/s12891-015-0700-5 26336889 PMC4559905

[19] Gagne JJ, Glynn RJ, Avorn J, Levin R, Schneeweiss S. A combined comorbidity score predicted mortality in elderly patients better than existing scores. J Clin Epidemiol. 2011;64(7):749–59. Epub 2011/01/07. 10.1016/j.jclinepi.2010.10.004 21208778 PMC3100405

[20] Scott JR, Ernst HM, Rotenberg BW, Rudmik L, Sowerby LJ. Oral corticosteroid prescribing habits for rhinosinusitis: The American Rhinologic Society membership. Am J Rhinol Allergy. 2017;31(1):22–6. Epub 2017/02/25. 10.2500/ajra.2017.31.4396 . 28234148

[21] Wang SV, Verpillat P, Rassen JA, Patrick A, Garry EM, Bartels DB. Transparency and Reproducibility of Observational Cohort Studies Using Large Healthcare Databases. Clin Pharmacol Ther. 2016;99(3):325–32. Epub 2015/12/23. 10.1002/cpt.329 . 26690726 PMC7833029

[22] Fralick M, Schneeweiss S, Patorno E. Risk of Diabetic Ketoacidosis after Initiation of an SGLT2 Inhibitor. N Engl J Med. 2017;376(23):2300–2. Epub 2017/06/08. 10.1056/NEJMc1701990 . 28591538

[23] Kim SC, Solomon DH, Rogers JR, Gale S, Klearman M, Sarsour K, et al Cardiovascular Safety of Tocilizumab Versus Tumor Necrosis Factor Inhibitors in Patients With Rheumatoid Arthritis: A Multi-Database Cohort Study. Arthritis Rheumatol. 2017;69(6):1154–64. Epub 2017/03/01. 10.1002/art.40084 28245350 PMC5573926

[24] VanderWeele TJ, Ding P. Sensitivity Analysis in Observational Research: Introducing the E-Value. Ann Intern Med. 2017;167(4):268–74. Epub 2017/07/12. 10.7326/M16-2607 . 28693043

[25] Hersh AL, Shapiro DJ, Pavia AT, Fleming-Dutra KE, Hicks LA. Geographic Variability in Diagnosis and Antibiotic Prescribing for Acute Respiratory Tract Infections. Infect Dis Ther. 2018;7(1):171–4. Epub 2017/12/24. 10.1007/s40121-017-0181-y 29273976 PMC5840100

[26] Stern A, Skalsky K, Avni T, Carrara E, Leibovici L, Paul M. Corticosteroids for pneumonia. Cochrane Database Syst Rev. 2017;12:CD007720 Epub 2017/12/14. 10.1002/14651858.CD007720.pub3 . 29236286 PMC6486210

[27] Da Silva JA, Jacobs JW, Kirwan JR, Boers M, Saag KG, Ines LB, et al Safety of low dose glucocorticoid treatment in rheumatoid arthritis: published evidence and prospective trial data. Ann Rheum Dis. 2006;65(3):285–93. Epub 2005/08/19. 10.1136/ard.2005.038638 16107513 PMC1798053

[28] Hoes JN, Jacobs JW, Boers M, Boumpas D, Buttgereit F, Caeyers N, et al EULAR evidence-based recommendations on the management of systemic glucocorticoid therapy in rheumatic diseases. Ann Rheum Dis. 2007;66(12):1560–7. Epub 2007/07/31. 10.1136/ard.2007.072157 17660219 PMC2095301

[29] Uppalapati SS, Boylan JD, Stoltzfus J. Risk factors involved in patients with bleeding peptic ulcers: a case-control study. Dig Dis Sci. 2009;54(3):593–8. Epub 2008/07/24. 10.1007/s10620-008-0387-7 . 18648934

[30] Leibovici L. Bacteraemia in the very old. Features and treatment. Drugs Aging. 1995;6(6):456–64. Epub 1995/06/01. 10.2165/00002512-199506060-00005 . 7663065

[31] Montagnana M, Favaloro EJ, Franchini M, Guidi GC, Lippi G. The role of ethnicity, age and gender in venous thromboembolism. J Thromb Thrombolysis. 2010;29(4):489–96. Epub 2009/06/19. 10.1007/s11239-009-0365-8 . 19536458

[32] Kanis JA, Johnell O, Oden A, Dawson A, De Laet C, Jonsson B. Ten year probabilities of osteoporotic fractures according to BMD and diagnostic thresholds. Osteoporos Int. 2001;12(12):989–95. Epub 2002/02/16. 10.1007/s001980170006 . 11846333

[33] Choudhry NK, Shrank WH. Four-dollar generics—increased accessibility, impaired quality assurance. N Engl J Med. 2010;363(20):1885–7. Epub 2010/11/12. 10.1056/NEJMp1006189 21067379

[34] Zgierska A, Rabago D, Miller MM. Impact of patient satisfaction ratings on physicians and clinical care. Patient Prefer Adherence. 2014;8:437–46. Epub 2014/04/15. 10.2147/PPA.S59077 24729691 PMC3979780

