# Supplementary material for: Prescribing systemic steroids for acute respiratory tract infections in United States outpatient settings: A nationwide population-based cohort study
Source: PLoS Med. 2020 Mar 31;17(3):e1003058. doi: 10.1371/journal.pmed.1003058 (PMC7108689; doi:10.1371/journal.pmed.1003058)
Supplement: S1 Text — (DOCX) [file pmed.1003058.s002.docx]

**S1 Text: Study protocol and definitions of the study variables**

## Study aims

To provide a more comprehensive assessment of US prescribing of systemic steroids for ARTI, we used a large claims database across 10 years with nation-wide coverage and stratified patients by whether they received steroids associated with ARTI diagnosis via oral, intravenous, and intramuscular routes. We also sought to determine the regional differences and other predictors associated with this practice, since prior analyses have revealed that treating ARTI with steroid injections might be a common practice in the southern US.

## Methods

## Data Sources

### Truven MarketScan Databases

The Truven MarketScan databases capture longitudinal, individual-level administrative claims data from the United States. The data available for study included three components of MarketScan: the Commercial Claims and Encounters (CCAE) Database, the Medicare Supplemental and Coordination of Benefits Database (MDCR), and the Medicaid Database. Patients in the databases are active employees, dependents, retirees, COBRA recipients, and Medicare or Medicaid enrollees. Data were drawn from large employers, health plans, and public organizations in the United States. The following tables of the MarketScan databases were available for analysis: Enrollment Detail, Inpatient Admissions, Inpatient Services, Outpatient Services, Outpatient Pharmaceutical Claims, and Long Term Care. These tables provide information on plan enrollment, healthcare utilization and expenditures, demographics, and integrated records for inpatient events, outpatient events, and pharmacy dispensings. Unless otherwise noted, drug event duration was calculated from the “Days Supply” field, and in cases where this field was 0, the duration was assumed to be 1 day. Data were available from December 31, 2002 to January 1, 2017, and represent approximately 185.3 million patients.

### General Notes on Administrative Data

Electronic outpatient pharmacy dispensing records are considered accurate because pharmacists fill prescriptions with little room for interpretation, and are reimbursed by insurers on the basis of detailed, complete, and accurate claims submitted electronically.^1,2^ Pharmacy dispensing information is usually seen as the gold standard of drug exposure information compared to self-reported information^3^ or prescribing records in outpatient medical records.^4^ Drugs used during hospital stays are not recorded in this data source. Prescribing information based on physician notes may overestimate actual medication use because up to 50% of prescriptions are never filled at the pharmacy.^5^

## Subjects

The study population was derived from patients aged 18 or older with an ARTI diagnosis recorded in an ambulatory visit between Jan 1, 2007 and Dec 31, 2016 without the same diagnosis recorded in the preceding 180 days. Eligible ARTI diagnoses included acute bronchitis, sinusitis, pharyngitis, otitis media, allergic rhinitis, influenza, pneumonia, and unspecified acute upper respiratory infections. To avoid including injectable or oral steroids prescribed in the context of patients with severe arthritis, we excluded encounters associated with rheumatology or orthopedic services as well as those with diagnoses of non-infectious arthritis or spondylosis on the cohort entry date and the preceding 180 days. We excluded patients if they were in nursing home in the 180 days prior to the cohort entry date (drug exposure data not available for these institutionalized patients). To ensure we have sufficient data to assess baseline co-morbidities, patients were required to have continuous insurance enrollment and drug benefit coverage during the 365 days prior to cohort entry date. Patients were excluded if aged 65 or older, owing to their eligibility for the federal Medicare program (Truven only has Medicare Supplemental but not fee-for-service Medicare claims). In addition, we excluded patients who were prescribed systemic steroids or with the medical conditions in the 365 days prior to the cohort entry date for which systemic steroids may be appropriate. These conditions include asthma, chronic obstructive pulmonary disease, inflammatory bowel disease, malignant neoplasm, organ transplant, interstitial lung disease, urticaria, rheumatoid arthritis, systemic lupus erythematosus, and systemic vasculitis (see Appendix 1 for definitions of these conditions).Only a single cohort entry was allowed per patient. If a patient qualified for the cohort multiple times, the patient entered the cohort on the first qualifying event before exclusion criteria were applied.

See the below for detailed definitions of the inclusion criteria and exclusion Criteria:

**Eligible acute respiratory tract infections (ARTIs) conditions:**

**Influenza (ICD 9&10): At least 1 outpatient code for**: { "487", "487.0", "487.1", "487.8", "488", "488.0", "488.01", "488.02", "488.09", "488.1", "488.11", "488.12", "488.19", "488.8", "488.81", "488.82", "488.89", "J09", "J09.X", "J09.X1", "J09.X2", "J09.X3", "J09.X9", "J10", "J10.0", "J10.00", "J10.01", "J10.08", "J10.1", "J10.2", "J10.8", "J10.81", "J10.82", "J10.83", "J10.89", "J11", "J11.0", "J11.00", "J11.08", "J11.1", "J11.2", "J11.8", "J11.81", "J11.82", "J11.83", "J11.89" }

**Acute Otitis media (ICD 9&10):**  **At least 1 outpatient code for**: { “381.0”, “381.00”, “381.01”, “381.02”, “381.03”, “381.04”, “381.05”, “381.06”, “381.51”, “382.0”, “382.00”, “382.01”, “382.02”, “H65.0”, “H65.00”, “H65.01”, “H65.02”, “H65.03”, “H65.04”, “H65.05”, “H65.06”, “H65.07”, “H65.1”, “H65.11”, “H65.111”, “H65.112”, “H65.113”, “H65.114”, “H65.115”, “H65.116”, “H65.117”, “H65.119”, “H65.19”, “H65.191”, “H65.192”, “H65.193”, “H65.194”, “H65.195”, “H65.196”, “H65.197”, “H65.199”, “H66.0”, “H66.00”, “H66.001”, “H66.002”, “H66.003”, “H66.004”, “H66.005”, “H66.006”, “H66.007”, “H66.009”, “H66.01”, “H66.011”, “H66.012”, “H66.013”, “H66.014”, “H66.015”, “H66.016”, “H66.017”, “H66.019” }

**Pneumonia (ICD 9&10): At least 1 outpatient code for**: { "480", "480.0", "480.1", "480.2", "480.3", "480.8", "480.9", "481", "482", "482.0", "482.1", "482.2", "482.3", "482.30", "482.31", "482.32", "482.39", "482.4", "482.40", "482.41", "482.42", "482.49", "482.8", "482.81", "482.82", "482.83", "482.84", "482.89", "482.9", "483", "483.0", "483.1", "483.8", "484", "484.1", "484.3", "484.5", "484.6", "484.7", "484.8", "485", "486", "487.0", "507", "507.0", "507.1", "507.8", "J12", "J12.0", "J12.1", "J12.2", "J12.3", "J12.8", "J12.81", "J12.89", "J12.9", "J13", "J14", "J15", "J15.0", "J15.1", "J15.2", "J15.20", "J15.21", "J15.211", "J15.212", "J15.29", "J15.3", "J15.4", "J15.5", "J15.6", "J15.7", "J15.8", "J15.9", "J16", "J16.0", "J16.8", "J17", "J18", "J18.0", "J18.1", "J18.2", "J18.8", "J18.9" }

**Acute bronchitis (ICD 9& 10): At least 1 outpatient code for**: { "466", "466.0", "466.1", "466.11", "466.19", "J20", "J20.0", "J20.1", "J20.2", "J20.3", "J20.4", "J20.5", "J20.6", "J20.7", "J20.8", "J20.9", "J21", "J21.0", "J21.1", "J21.8", "J21.9" }

**Acute pharyngitis (ICD 9&10): At least 1 outpatient code for**: { "462", "463", "464", "464.0", "464.00", "464.01", "464.1", "464.10", "464.11", "464.2", "464.20", "464.21", "464.3", "464.30", "464.31", "464.4", "464.5", "464.50", "464.51", "J02", "J02.0", "J02.8", "J02.9", "J03", "J03.0", "J03.00", "J03.01", "J03.8", "J03.80", "J03.81", "J03.9", "J03.90", "J03.91" }

**Acute sinusitis (ICD 9&10): At least 1 outpatient code for**: { "461", "461.0", "461.1", "461.2", "461.3", "461.8", "461.9", "J01", "J01.0", "J01.00", "J01.01", "J01.1", "J01.10", "J01.11", "J01.2", "J01.20", "J01.21", "J01.3", "J01.30", "J01.31", "J01.4", "J01.40", "J01.41", "J01.8", "J01.80", "J01.81", "J01.9", "J01.90", "J01.91" }

**Acute upper respiratory infections (ICD 9 & 10): At least 1 outpatient code for**: { "460", "465", "465.0", "465.8", "465.9", "J00", "J06", "J06.0", "J06.9" }

**Allergic rhinitis (ICD 9& 10): At least 1 outpatient code for**: { "477", "477.0", "477.1", "477.2", "477.8", "477.9", "J30", "J30.0", "J30.1", "J30.2", "J30.5", "J30.8", "J30.81", "J30.89", "J30.9" }

**Exclusion criteria conditions :**

To avoid including injectable or oral steroids prescribed in the context of patients with severe arthritis, we excluded encounters associated with rheumatology or orthopedic services as well as those with diagnoses of non-infectious arthritis or spondylosis on the cohort entry date and the preceding 180 days. We also did not include encounters occurred in a surgical specialty clinic except for otolaryngology (ENT).

**Asthma (ICD9&10): At least 1 inpatient or outpatient code for**: { "493.0", "493.00", "493.01", "493.02", "493.1", "493.10", "493.11", "493.12", "493.2", "493.20", "493.21", "493.22", "493.8", "493.81", "493.82", "493.9", "493.90", "493.91", "493.92", "J45.20", "J45.21", "J45.22", "J45.30", "J45.31", "J45.32", "J45.40", "J45.41", "J45.42", "J45.50", "J45.51", "J45.52", "J45.901", "J45.902", "J45.909", "J45.990", "J45.991", "J45.998" }

**COPD (ICD9&10): At least 1 inpatient or outpatient code for**: { "491.0", "491.1", "491.2", "491.20", "491.21", "491.22", "491.8", "491.9", "492.0", "492.8", "496", "J41.0", "J41.1", "J41.8", "J42", "J43.0", "J43.1", "J43.2", "J43.8", "J43.9", "J44.0", "J44.1", "J44.9", "J43" }

**Urticaria (ICD9&10): At least 1 inpatient or outpatient code for:** : { "708", "708.0", "708.1", "708.2", "708.3", "708.4", "708.5", "708.8", "708.9", "L50.0", "L50.1", "L50.2", "L50.3", "L50.4", "L50.5", "L50.6", "L50.8", "L50.9" }

**Systemic vasculitis (ICD9&10): At least 1 inpatient or outpatient code for:** : { "446", "446.0", "446.1", "446.2", "446.20", "446.21", "446.29", "446.3", "446.4", "446.5", "446.6", "446.7", "447.6”, "I77.6", "M30.0", "M30.3", "M31.0", "M31.1", "M31.2", "M31.30", "M31.4", "M31.6" }

**Systemic lupus erythematosus (ICD9&10): At least 1 inpatient or outpatient code for:** : { "710.0", "M32.10" }

**Rheumatoid arthritis (ICD9&10): At least 1 inpatient or outpatient code for:** : { "714.0", "714.1", "714.2", "714.3", "714.30", "714.31", "714.32", "714.33", “M05.00”, “M05.30”, “M05.60”, “M06.1”, “M06.9”, “M08.00”, “M08.3”, “M08.40”  }

**Spondyloarthropathy: At least 1 inpatient or outpatient code for:** : { "721.0", "721", "721.1", "721.2", "721.3", "721.4", "721.41", "721.42", "721.9", "721.90", "721.91", "M47.10", "M47.12", "M47.14", "M47.16", "M47.812", "M47.814", "M47.817", "M47.819" }

**Non-septic arthritis (ICD9&10): At least 1 inpatient or outpatient code for:** : { "710", "710.0", "710.1", "710.2", "710.3", "710.4", "710.5", "710.8", "710.9", "711.1", "711.10", "711.11", "711.12", "711.13", "711.14", "711.15", "711.16", "711.17", "711.18", "711.19", "711.2", "711.20", "711.21", "711.22", "711.23", "711.24", "711.25", "711.26", "711.27", "711.28", "711.29", "711.3", "711.30", "711.31", "711.32", "711.33", "711.34", "711.35", "711.36", "711.37", "711.38", "711.39", "711.5", "711.50", "711.51", "711.52", "711.53", "711.54", "711.55", "711.56", "711.57", "711.58", "711.59", "712", "712.1", "712.10", "712.11", "712.12", "712.13", "712.14", "712.15", "712.16", "712.17", "712.18", "712.19", "712.2", "712.20", "712.21", "712.22", "712.23", "712.24", "712.25", "712.26", "712.27", "712.28", "712.29", "712.3", "712.30", "712.31", "712.32", "712.33", "712.34", "712.35", "712.36", "712.37", "712.38", "712.39", "712.8", "712.80", "712.81", "712.82", "712.83", "712.84", "712.85", "712.86", "712.87", "712.88", "712.89", "712.9", "712.90", "712.91", "712.92", "712.93", "712.94", "712.95", "712.96", "712.97", "712.98", "712.99", "713", "713.0", "713.1", "713.2", "713.3", "713.4", "713.5", "713.6", "713.7", "713.8", "714", "714.0", "714.1", "714.2", "714.3", "714.30", "714.31", "714.32", "714.33", "714.4", "714.8", "714.81", "714.89", "714.9", "715", "715.0", "715.00", "715.04", "715.09", "715.1", "715.10", "715.11", "715.12", "715.13", "715.14", "715.15", "715.16", "715.17", "715.18", "715.2", "715.20", "715.21", "715.22", "715.23", "715.24", "715.25", "715.26", "715.27", "715.28", "715.3", "715.30", "715.31", "715.32", "715.33", "715.34", "715.35", "715.36", "715.37", "715.38", "715.8", "715.80", "715.89", "715.9", "715.90", "715.91", "715.92", "715.93", "715.94", "715.95", "715.96", "715.97", "715.98", "716", "716.0", "716.00", "716.01", "716.02", "716.03", "716.04", "716.05", "716.06", "716.07", "716.08", "716.09", "716.1", "716.10", "716.11", "716.12", "716.13", "716.14", "716.15", "716.16", "716.17", "716.18", "716.19", "716.2", "716.20", "716.21", "716.22", "716.23", "716.24", "716.25", "716.26", "716.27", "716.28", "716.29", "716.3", "716.30", "716.31", "716.32", "716.33", "716.34", "716.35", "716.36", "716.37", "716.38", "716.39", "716.4", "716.40", "716.41", "716.42", "716.43", "716.44", "716.45", "716.46", "716.47", "716.48", "716.49", "716.5", "716.50", "716.51", "716.52", "716.53", "716.54", "716.55", "716.56", "716.57", "716.58", "716.59", "716.6", "716.60", "716.61", "716.62", "716.63", "716.64", "716.65", "716.66", "716.67", "716.68", "716.8", "716.80", "716.81", "716.82", "716.83", "716.84", "716.85", "716.86", "716.87", "716.88", "716.89", "716.9", "716.90", "716.91", "716.92", "716.93", "716.94", "716.95", "716.96", "716.97", "716.98", "716.99", "718", "718.0", "718.00", "718.01", "718.02", "718.03", "718.04", "718.05", "718.07", "718.08", "718.09", "718.1", "718.10", "718.11", "718.12", "718.13", "718.14", "718.15", "718.17", "718.18", "718.19", "718.2", "718.20", "718.21", "718.22", "718.23", "718.24", "718.25", "718.26", "718.27", "718.28", "718.29", "718.3", "718.30", "718.31", "718.32", "718.33", "718.34", "718.35", "718.36", "718.37", "718.38", "718.39", "718.4", "718.40", "718.41", "718.42", "718.43", "718.44", "718.45", "718.46", "718.47", "718.48", "718.49", "718.5", "718.50", "718.51", "718.52", "718.53", "718.54", "718.55", "718.56", "718.57", "718.58", "718.59", "718.6", "718.60", "718.65", "718.7", "718.70", "718.71", "718.72", "718.73", "718.74", "718.75", "718.76", "718.77", "718.78", "718.79", "718.8", "718.80", "718.81", "718.82", "718.83", "718.84", "718.85", "718.86", "718.87", "718.88", "718.89", "718.9", "718.90", "718.91", "718.92", "718.93", "718.94", "718.95", "718.97", "718.98", "718.99", "719", "719.0", "719.00", "719.01", "719.02", "719.03", "719.04", "719.05", "719.06", "719.07", "719.08", "719.09", "719.1", "719.10", "719.11", "719.12", "719.13", "719.14", "719.15", "719.16", "719.17", "719.18", "719.19", "719.2", "719.20", "719.21", "719.22", "719.23", "719.24", "719.25", "719.26", "719.27", "719.28", "719.29", "719.3", "719.30", "719.31", "719.32", "719.33", "719.34", "719.35", "719.36", "719.37", "719.38", "719.39", "719.4", "719.40", "719.41", "719.42", "719.43", "719.44", "719.45", "719.46", "719.47", "719.48", "719.49", "719.5", "719.50", "719.51", "719.52", "719.53", "719.54", "719.55", "719.56", "719.57", "719.58", "719.59", "719.6", "719.60", "719.61", "719.62", "719.63", "719.64", "719.65", "719.66", "719.67", "719.68", "719.69", "719.7", "719.8", "719.80", "719.81", "719.82", "719.83", "719.84", "719.85", "719.86", "719.87", "719.88", "719.89", "719.9", "719.90", "719.91", "719.92", "719.93", "719.94", "719.95", "719.96", "719.97", "719.98", "719.99", "V13.4", "274", "274.0", "274.00", "274.01", "274.02", "274.03", "274.1", "274.10", "274.19", "274.8", "274.81", "274.82", "274.89", "274.9" }

**History of Malignant Neoplasm (ICD9&10): At least 1 inpatient or outpatient code for**: { "140", "140.0", "140.1", "140.3", "140.4", "140.5", "140.6", "140.8", "140.9", "141", "141.0", "141.1", "141.2", "141.3", "141.4", "141.5", "141.6", "141.8", "141.9", "142", "142.0", "142.1", "142.2", "142.8", "142.9", "143", "143.0", "143.1", "143.8", "143.9", "144", "144.0", "144.1", "144.8", "144.9", "145", "145.0", "145.1", "145.2", "145.3", "145.4", "145.5", "145.6", "145.8", "145.9", "146", "146.0", "146.1", "146.2", "146.3", "146.4", "146.5", "146.6", "146.7", "146.8", "146.9", "147", "147.0", "147.1", "147.2", "147.3", "147.8", "147.9", "148", "148.0", "148.1", "148.2", "148.3", "148.8", "148.9", "149", "149.0", "149.1", "149.8", "149.9", "150", "150.0", "150.1", "150.2", "150.3", "150.4", "150.5", "150.8", "150.9", "151", "151.0", "151.1", "151.2", "151.3", "151.4", "151.5", "151.6", "151.8", "151.9", "152", "152.0", "152.1", "152.2", "152.3", "152.8", "152.9", "153", "153.0", "153.1", "153.2", "153.3", "153.4", "153.5", "153.6", "153.7", "153.8", "153.9", "154", "154.0", "154.1", "154.2", "154.3", "154.8", "155", "155.0", "155.1", "155.2", "156", "156.0", "156.1", "156.2", "156.8", "156.9", "157", "157.0", "157.1", "157.2", "157.3", "157.4", "157.8", "157.9", "158", "158.0", "158.8", "158.9", "159", "159.0", "159.1", "159.8", "159.9", "160", "160.0", "160.1", "160.2", "160.3", "160.4", "160.5", "160.8", "160.9", "161", "161.0", "161.1", "161.2", "161.3", "161.8", "161.9", "162", "162.0", "162.2", "162.3", "162.4", "162.5", "162.8", "162.9", "163", "163.0", "163.1", "163.8", "163.9", "164", "164.0", "164.1", "164.2", "164.3", "164.8", "164.9", "165", "165.0", "165.8", "165.9", "170", "170.0", "170.1", "170.2", "170.3", "170.4", "170.5", "170.6", "170.7", "170.8", "170.9", "171", "171.0", "171.2", "171.3", "171.4", "171.5", "171.6", "171.7", "171.8", "171.9", "172", "172.0", "172.1", "172.2", "172.3", "172.4", "172.5", "172.6", "172.7", "172.8", "172.9", "174", "174.0", "174.1", "174.2", "174.3", "174.4", "174.5", "174.6", "174.8", "174.9", "175", "175.0", "175.9", "176", "176.0", "176.1", "176.2", "176.3", "176.4", "176.5", "176.8", "176.9", "179", "180", "180.0", "180.1", "180.8", "180.9", "181", "182", "182.0", "182.1", "182.8", "183", "183.0", "183.2", "183.3", "183.4", "183.5", "183.8", "183.9", "184", "184.0", "184.1", "184.2", "184.3", "184.4", "184.8", "184.9", "185", "186", "186.0", "186.9", "187", "187.1", "187.2", "187.3", "187.4", "187.5", "187.6", "187.7", "187.8", "187.9", "188", "188.0", "188.1", "188.2", "188.3", "188.4", "188.5", "188.6", "188.7", "188.8", "188.9", "189", "189.0", "189.1", "189.2", "189.3", "189.4", "189.8", "189.9", "190", "190.0", "190.1", "190.2", "190.3", "190.4", "190.5", "190.6", "190.7", "190.8", "190.9", "191", "191.0", "191.1", "191.2", "191.3", "191.4", "191.5", "191.6", "191.7", "191.8", "191.9", "192", "192.0", "192.1", "192.2", "192.3", "192.8", "192.9", "193", "194", "194.0", "194.1", "194.3", "194.4", "194.5", "194.6", "194.8", "194.9", "195", "195.0", "195.1", "195.2", "195.3", "195.4", "195.5", "195.8", "196", "196.0", "196.1", "196.2", "196.3", "196.5", "196.6", "196.8", "196.9", "197", "197.0", "197.1", "197.2", "197.3", "197.4", "197.5", "197.6", "197.7", "197.8", "198", "198.0", "198.1", "198.2", "198.3", "198.4", "198.5", "198.6", "198.7", "198.8", "198.81", "198.82", "198.89", "199", "199.0", "199.1", "199.2", "200", "200.0", "200.00", "200.01", "200.02", "200.03", "200.04", "200.05", "200.06", "200.07", "200.08", "200.1", "200.10", "200.11", "200.12", "200.13", "200.14", "200.15", "200.16", "200.17", "200.18", "200.2", "200.20", "200.21", "200.22", "200.23", "200.24", "200.25", "200.26", "200.27", "200.28", "200.3", "200.30", "200.31", "200.32", "200.33", "200.34", "200.35", "200.36", "200.37", "200.38", "200.4", "200.40", "200.41", "200.42", "200.43", "200.44", "200.45", "200.46", "200.47", "200.48", "200.5", "200.50", "200.51", "200.52", "200.53", "200.54", "200.55", "200.56", "200.57", "200.58", "200.6", "200.60", "200.61", "200.62", "200.63", "200.64", "200.65", "200.66", "200.67", "200.68", "200.7", "200.70", "200.71", "200.72", "200.73", "200.74", "200.75", "200.76", "200.77", "200.78", "200.8", "200.80", "200.81", "200.82", "200.83", "200.84", "200.85", "200.86", "200.87", "200.88", "201", "201.0", "201.00", "201.01", "201.02", "201.03", "201.04", "201.05", "201.06", "201.07", "201.08", "201.1", "201.10", "201.11", "201.12", "201.13", "201.14", "201.15", "201.16", "201.17", "201.18", "201.2", "201.20", "201.21", "201.22", "201.23", "201.24", "201.25", "201.26", "201.27", "201.28", "201.4", "201.40", "201.41", "201.42", "201.43", "201.44", "201.45", "201.46", "201.47", "201.48", "201.5", "201.50", "201.51", "201.52", "201.53", "201.54", "201.55", "201.56", "201.57", "201.58", "201.6", "201.60", "201.61", "201.62", "201.63", "201.64", "201.65", "201.66", "201.67", "201.68", "201.7", "201.70", "201.71", "201.72", "201.73", "201.74", "201.75", "201.76", "201.77", "201.78", "201.9", "201.90", "201.91", "201.92", "201.93", "201.94", "201.95", "201.96", "201.97", "201.98", "202", "202.0", "202.00", "202.01", "202.02", "202.03", "202.04", "202.05", "202.06", "202.07", "202.08", "202.1", "202.10", "202.11", "202.12", "202.13", "202.14", "202.15", "202.16", "202.17", "202.18", "202.2", "202.20", "202.21", "202.22", "202.23", "202.24", "202.25", "202.26", "202.27", "202.28", "202.3", "202.30", "202.31", "202.32", "202.33", "202.34", "202.35", "202.36", "202.37", "202.38", "202.4", "202.40", "202.41", "202.42", "202.43", "202.44", "202.45", "202.46", "202.47", "202.48", "202.5", "202.50", "202.51", "202.52", "202.53", "202.54", "202.55", "202.56", "202.57", "202.58", "202.6", "202.60", "202.61", "202.62", "202.63", "202.64", "202.65", "202.66", "202.67", "202.68", "202.7", "202.70", "202.71", "202.72", "202.73", "202.74", "202.75", "202.76", "202.77", "202.78", "202.8", "202.80", "202.81", "202.82", "202.83", "202.84", "202.85", "202.86", "202.87", "202.88", "202.9", "202.90", "202.91", "202.92", "202.93", "202.94", "202.95", "202.96", "202.97", "202.98", "203", "203.0", "203.00", "203.01", "203.02", "203.1", "203.10", "203.11", "203.12", "203.8", "203.80", "203.81", "203.82", "204", "204.0", "204.00", "204.01", "204.02", "204.1", "204.10", "204.11", "204.12", "204.2", "204.20", "204.21", "204.22", "204.8", "204.80", "204.81", "204.82", "204.9", "204.90", "204.91", "204.92", "205", "205.0", "205.00", "205.01", "205.02", "205.1", "205.10", "205.11", "205.12", "205.2", "205.20", "205.21", "205.22", "205.3", "205.30", "205.31", "205.32", "205.8", "205.80", "205.81", "205.82", "205.9", "205.90", "205.91", "205.92", "206", "206.0", "206.00", "206.01", "206.02", "206.1", "206.10", "206.11", "206.12", "206.2", "206.20", "206.21", "206.22", "206.8", "206.80", "206.81", "206.82", "206.9", "206.90", "206.91", "206.92", "207", "207.0", "207.00", "207.01", "207.02", "207.1", "207.10", "207.11", "207.12", "207.2", "207.20", "207.21", "207.22", "207.8", "207.80", "207.81", "207.82", "208", "208.0", "208.00", "208.01", "208.02", "208.1", "208.10", "208.11", "208.12", "208.2", "208.20", "208.21", "208.22", "208.8", "208.80", "208.81", "208.82", "208.9", "208.90", "208.91", "208.92", "C00.0", "C00.1", "C00.2", "C00.3", "C00.4", "C00.5", "C00.6", "C00.8", "C00.9", "C01", "C02.0", "C02.1", "C02.2", "C02.3", "C02.4", "C02.8", "C02.9", "C03.0", "C03.1", "C03.9", "C04.0", "C04.1", "C04.8", "C04.9", "C05.0", "C05.1", "C05.2", "C05.8", "C05.9", "C06.0", "C06.1", "C06.2", "C06.80", "C06.89", "C06.9", "C07", "C08.0", "C08.1", "C08.9", "C09.0", "C09.1", "C09.8", "C09.9", "C10.0", "C10.1", "C10.2", "C10.3", "C10.4", "C10.8", "C10.9", "C11.0", "C11.1", "C11.2", "C11.3", "C11.8", "C11.9", "C12", "C13.0", "C13.1", "C13.2", "C13.8", "C13.9", "C14.0", "C14.2", "C14.8", "C15.3", "C15.4", "C15.5", "C15.8", "C15.9", "C16.0", "C16.1", "C16.2", "C16.3", "C16.4", "C16.5", "C16.6", "C16.8", "C16.9", "C17.0", "C17.1", "C17.2", "C17.3", "C17.8", "C17.9", "C18.0", "C18.1", "C18.2", "C18.3", "C18.4", "C18.5", "C18.6", "C18.7", "C18.8", "C18.9", "C19", "C20", "C21.0", "C21.1", "C21.2", "C21.8", "C22.0", "C22.1", "C22.2", "C22.3", "C22.4", "C22.7", "C22.8", "C22.9", "C23", "C24.0", "C24.1", "C24.8", "C24.9", "C25.0", "C25.1", "C25.2", "C25.3", "C25.4", "C25.7", "C25.8", "C25.9", "C26.0", "C26.1", "C26.9", "C30.0", "C30.1", "C31.0", "C31.1", "C31.2", "C31.3", "C31.8", "C31.9", "C32.0", "C32.1", "C32.2", "C32.3", "C32.8", "C32.9", "C33", "C34.00", "C34.01", "C34.02", "C34.10", "C34.11", "C34.12", "C34.2", "C34.30", "C34.31", "C34.32", "C34.80", "C34.81", "C34.82", "C34.90", "C34.91", "C34.92", "C37", "C38.0", "C38.1", "C38.2", "C38.3", "C38.4", "C38.8", "C39.0", "C39.9", "C40.00", "C40.01", "C40.02", "C40.10", "C40.11", "C40.12", "C40.20", "C40.21", "C40.22", "C40.30", "C40.31", "C40.32", "C40.80", "C40.81", "C40.82", "C40.90", "C40.91", "C40.92", "C41.0", "C41.1", "C41.2", "C41.3", "C41.4", "C41.9", "C43.0", "C43.10", "C43.11", "C43.12", "C43.20", "C43.21", "C43.22", "C43.30", "C43.31", "C43.39", "C43.4", "C43.51", "C43.52", "C43.59", "C43.60", "C43.61", "C43.62", "C43.70", "C43.71", "C43.72", "C43.8", "C43.9", "C45.0", "C45.1", "C45.2", "C45.7", "C45.9", "C46.0", "C46.1", "C46.2", "C46.3", "C46.4", "C46.50", "C46.51", "C46.52", "C46.7", "C46.9", "C47.0", "C47.10", "C47.11", "C47.12", "C47.20", "C47.21", "C47.22", "C47.3", "C47.4", "C47.5", "C47.6", "C47.8", "C47.9", "C48.0", "C48.1", "C48.2", "C48.8", "C49.0", "C49.10", "C49.11", "C49.12", "C49.20", "C49.21", "C49.22", "C49.3", "C49.4", "C49.5", "C49.6", "C49.8", "C49.9", "C50.011", "C50.012", "C50.019", "C50.021", "C50.022", "C50.029", "C50.111", "C50.112", "C50.119", "C50.121", "C50.122", "C50.129", "C50.211", "C50.212", "C50.219", "C50.221", "C50.222", "C50.229", "C50.311", "C50.312", "C50.319", "C50.321", "C50.322", "C50.329", "C50.411", "C50.412", "C50.419", "C50.421", "C50.422", "C50.429", "C50.511", "C50.512", "C50.519", "C50.521", "C50.522", "C50.529", "C50.611", "C50.612", "C50.619", "C50.621", "C50.622", "C50.629", "C50.811", "C50.812", "C50.819", "C50.821", "C50.822", "C50.829", "C50.911", "C50.912", "C50.919", "C50.921", "C50.922", "C50.929", "C51.0", "C51.1", "C51.2", "C51.8", "C51.9", "C52", "C53.0", "C53.1", "C53.8", "C53.9", "C54.0", "C54.1", "C54.2", "C54.3", "C54.8", "C54.9", "C55", "C56.1", "C56.2", "C56.9", "C57.00", "C57.01", "C57.02", "C57.10", "C57.11", "C57.12", "C57.20", "C57.21", "C57.22", "C57.3", "C57.4", "C57.7", "C57.8", "C57.9", "C58", "C60.0", "C60.1", "C60.2", "C60.8", "C60.9", "C61", "C62.00", "C62.01", "C62.02", "C62.10", "C62.11", "C62.12", "C62.90", "C62.91", "C62.92", "C63.00", "C63.01", "C63.02", "C63.10", "C63.11", "C63.12", "C63.2", "C63.7", "C63.8", "C63.9", "C64.1", "C64.2", "C64.9", "C65.1", "C65.2", "C65.9", "C66.1", "C66.2", "C66.9", "C67.0", "C67.1", "C67.2", "C67.3", "C67.4", "C67.5", "C67.6", "C67.7", "C67.8", "C67.9", "C68.0", "C68.1", "C68.8", "C68.9", "C69.00", "C69.01", "C69.02", "C69.10", "C69.11", "C69.12", "C69.20", "C69.21", "C69.22", "C69.30", "C69.31", "C69.32", "C69.40", "C69.41", "C69.42", "C69.50", "C69.51", "C69.52", "C69.60", "C69.61", "C69.62", "C69.80", "C69.81", "C69.82", "C69.90", "C69.91", "C69.92", "C70.0", "C70.1", "C70.9", "C71.0", "C71.1", "C71.2", "C71.3", "C71.4", "C71.5", "C71.6", "C71.7", "C71.8", "C71.9", "C72.0", "C72.1", "C72.20", "C72.21", "C72.22", "C72.30", "C72.31", "C72.32", "C72.40", "C72.41", "C72.42", "C72.50", "C72.59", "C72.9", "C73", "C74.00", "C74.01", "C74.02", "C74.10", "C74.11", "C74.12", "C74.90", "C74.91", "C74.92", "C75.0", "C75.1", "C75.2", "C75.3", "C75.4", "C75.5", "C75.8", "C75.9", "C76.0", "C76.1", "C76.2", "C76.3", "C76.40", "C76.41", "C76.42", "C76.50", "C76.51", "C76.52", "C76.8", "C77.0", "C77.1", "C77.2", "C77.3", "C77.4", "C77.5", "C77.8", "C77.9", "C78.00", "C78.01", "C78.02", "C78.1", "C78.2", "C78.30", "C78.39", "C78.4", "C78.5", "C78.6", "C78.7", "C78.80", "C78.89", "C79.00", "C79.01", "C79.02", "C79.10", "C79.11", "C79.19", "C79.2", "C79.31", "C79.32", "C79.40", "C79.49", "C79.51", "C79.52", "C79.60", "C79.61", "C79.62", "C79.70", "C79.71", "C79.72", "C79.81", "C79.82", "C79.89", "C79.9", "C80.0", "C80.1", "C80.2", "C81.00", "C81.01", "C81.02", "C81.03", "C81.04", "C81.05", "C81.06", "C81.07", "C81.08", "C81.09", "C81.10", "C81.11", "C81.12", "C81.13", "C81.14", "C81.15", "C81.16", "C81.17", "C81.18", "C81.19", "C81.20", "C81.21", "C81.22", "C81.23", "C81.24", "C81.25", "C81.26", "C81.27", "C81.28", "C81.29", "C81.30", "C81.31", "C81.32", "C81.33", "C81.34", "C81.35", "C81.36", "C81.37", "C81.38", "C81.39", "C81.40", "C81.41", "C81.42", "C81.43", "C81.44", "C81.45", "C81.46", "C81.47", "C81.48", "C81.49", "C81.70", "C81.71", "C81.72", "C81.73", "C81.74", "C81.75", "C81.76", "C81.77", "C81.78", "C81.79", "C81.90", "C81.91", "C81.92", "C81.93", "C81.94", "C81.95", "C81.96", "C81.97", "C81.98", "C81.99", "C82.00", "C82.01", "C82.02", "C82.03", "C82.04", "C82.05", "C82.06", "C82.07", "C82.08", "C82.09", "C82.10", "C82.11", "C82.12", "C82.13", "C82.14", "C82.15", "C82.16", "C82.17", "C82.18", "C82.19", "C82.20", "C82.21", "C82.22", "C82.23", "C82.24", "C82.25", "C82.26", "C82.27", "C82.28", "C82.29", "C82.30", "C82.31", "C82.32", "C82.33", "C82.34", "C82.35", "C82.36", "C82.37", "C82.38", "C82.39", "C82.40", "C82.41", "C82.42", "C82.43", "C82.44", "C82.45", "C82.46", "C82.47", "C82.48", "C82.49", "C82.50", "C82.51", "C82.52", "C82.53", "C82.54", "C82.55", "C82.56", "C82.57", "C82.58", "C82.59", "C82.60", "C82.61", "C82.62", "C82.63", "C82.64", "C82.65", "C82.66", "C82.67", "C82.68", "C82.69", "C82.80", "C82.81", "C82.82", "C82.83", "C82.84", "C82.85", "C82.86", "C82.87", "C82.88", "C82.89", "C82.90", "C82.91", "C82.92", "C82.93", "C82.94", "C82.95", "C82.96", "C82.97", "C82.98", "C82.99", "C83.00", "C83.01", "C83.02", "C83.03", "C83.04", "C83.05", "C83.06", "C83.07", "C83.08", "C83.09", "C83.10", "C83.11", "C83.12", "C83.13", "C83.14", "C83.15", "C83.16", "C83.17", "C83.18", "C83.19", "C83.30", "C83.31", "C83.32", "C83.33", "C83.34", "C83.35", "C83.36", "C83.37", "C83.38", "C83.39", "C83.50", "C83.51", "C83.52", "C83.53", "C83.54", "C83.55", "C83.56", "C83.57", "C83.58", "C83.59", "C83.70", "C83.71", "C83.72", "C83.73", "C83.74", "C83.75", "C83.76", "C83.77", "C83.78", "C83.79", "C83.80", "C83.81", "C83.82", "C83.83", "C83.84", "C83.85", "C83.86", "C83.87", "C83.88", "C83.89", "C83.90", "C83.91", "C83.92", "C83.93", "C83.94", "C83.95", "C83.96", "C83.97", "C83.98", "C83.99", "C84.00", "C84.01", "C84.02", "C84.03", "C84.04", "C84.05", "C84.06", "C84.07", "C84.08", "C84.09", "C84.10", "C84.11", "C84.12", "C84.13", "C84.14", "C84.15", "C84.16", "C84.17", "C84.18", "C84.19", "C84.40", "C84.41", "C84.42", "C84.43", "C84.44", "C84.45", "C84.46", "C84.47", "C84.48", "C84.49", "C84.60", "C84.61", "C84.62", "C84.63", "C84.64", "C84.65", "C84.66", "C84.67", "C84.68", "C84.69", "C84.70", "C84.71", "C84.72", "C84.73", "C84.74", "C84.75", "C84.76", "C84.77", "C84.78", "C84.79", "C84.90", "C84.91", "C84.92", "C84.93", "C84.94", "C84.95", "C84.96", "C84.97", "C84.98", "C84.99", "C84.A0", "C84.A1", "C84.A2", "C84.A3", "C84.A4", "C84.A5", "C84.A6", "C84.A7", "C84.A8", "C84.A9", "C84.Z0", "C84.Z1", "C84.Z2", "C84.Z3", "C84.Z4", "C84.Z5", "C84.Z6", "C84.Z7", "C84.Z8", "C84.Z9", "C85.10", "C85.11", "C85.12", "C85.13", "C85.14", "C85.15", "C85.16", "C85.17", "C85.18", "C85.19", "C85.20", "C85.21", "C85.22", "C85.23", "C85.24", "C85.25", "C85.26", "C85.27", "C85.28", "C85.29", "C85.80", "C85.81", "C85.82", "C85.83", "C85.84", "C85.85", "C85.86", "C85.87", "C85.88", "C85.89", "C85.90", "C85.91", "C85.92", "C85.93", "C85.94", "C85.95", "C85.96", "C85.97", "C85.98", "C85.99", "C86.0", "C86.1", "C86.2", "C86.3", "C86.4", "C86.5", "C86.6", "C88.2", "C88.3", "C88.4", "C88.8", "C88.9", "C90.00", "C90.01", "C90.02", "C90.10", "C90.11", "C90.12", "C90.20", "C90.21", "C90.22", "C90.30", "C90.31", "C90.32", "C91.00", "C91.01", "C91.02", "C91.10", "C91.11", "C91.12", "C91.30", "C91.31", "C91.32", "C91.40", "C91.41", "C91.42", "C91.50", "C91.51", "C91.52", "C91.60", "C91.61", "C91.62", "C91.90", "C91.91", "C91.92", "C91.A0", "C91.A1", "C91.A2", "C91.Z0", "C91.Z1", "C91.Z2", "C92.00", "C92.01", "C92.02", "C92.10", "C92.11", "C92.12", "C92.20", "C92.21", "C92.22", "C92.30", "C92.31", "C92.32", "C92.40", "C92.41", "C92.42", "C92.50", "C92.51", "C92.52", "C92.60", "C92.61", "C92.62", "C92.90", "C92.91", "C92.92", "C92.A0", "C92.A1", "C92.A2", "C92.Z0", "C92.Z1", "C92.Z2", "C93.00", "C93.01", "C93.02", "C93.10", "C93.11", "C93.12", "C93.30", "C93.31", "C93.32", "C93.90", "C93.91", "C93.92", "C93.Z0", "C93.Z1", "C93.Z2", "C94.00", "C94.01", "C94.02", "C94.20", "C94.21", "C94.22", "C94.30", "C94.31", "C94.32", "C94.80", "C94.81", "C94.82", "C95.00", "C95.01", "C95.02", "C95.10", "C95.11", "C95.12", "C95.90", "C95.91", "C95.92", "C96.0", "C96.2", "C96.4", "C96.9", "C96.A", "C96.Z", "D03.0", "D03.10", "D03.11", "D03.12", "D03.20", "D03.21", "D03.22", "D03.30", "D03.39", "D03.4", "D03.51", "D03.52", "D03.59", "D03.60", "D03.61", "D03.62", "D03.70", "D03.71", "D03.72", "D03.8", "D03.9", "D45" }

**Organ Transplant (ICD9&10): Legacy Attribute - Procedure Code (Any Position)** is any of: { "33.5", "33.50", "33.51", "33.52", "33.6", "37.51", "41.0", "41.00", "41.01", "41.02", "41.03", "41.04", "41.05", "41.06", "41.07", "41.08", "41.09", "46.97", "50.5", "50.51", "50.59", "52.8", "52.80", "52.81", "52.82", "52.83", "52.84", "52.85", "52.86", "55.6", "55.61", "55.69", "02YA0Z0", "02YA0Z1", "02YA0Z2", "0BYC0Z0", "0BYC0Z1", "0BYC0Z2", "0BYD0Z0", "0BYD0Z1", "0BYD0Z2", "0BYF0Z0", "0BYF0Z1", "0BYF0Z2", "0BYG0Z0", "0BYG0Z1", "0BYG0Z2", "0BYH0Z0", "0BYH0Z1", "0BYH0Z2", "0BYJ0Z0", "0BYJ0Z1", "0BYJ0Z2", "0BYK0Z0", "0BYK0Z1", "0BYK0Z2", "0BYL0Z0", "0BYL0Z1", "0BYL0Z2", "0BYM0Z0", "0BYM0Z1", "0BYM0Z2", "0DY80Z0", "0DY80Z1", "0DY80Z2", "0DYE0Z0", "0DYE0Z1", "0DYE0Z2", "0FSG0ZZ", "0FSG4ZZ", "0FY00Z0", "0FY00Z1", "0FY00Z2", "0FYG0Z0", "0FYG0Z1", "0FYG0Z2", "0TS00ZZ", "0TS10ZZ", "0TY00Z0", "0TY00Z1", "0TY00Z2", "0TY10Z0", "0TY10Z1", "0TY10Z2", "30230AZ", "30230G0", "30230G2", "30230G3", "30230G4", "30230X0", "30230X2", "30230X3", "30230X4", "30230Y0", "30230Y2", "30230Y3", "30230Y4", "30233AZ", "30233G0", "30233G2", "30233G3", "30233G4", "30233X0", "30233X2", "30233X3", "30233X4", "30233Y0", "30233Y2", "30233Y3", "30233Y4", "30240AZ", "30240G0", "30240G2", "30240G3", "30240G4", "30240X0", "30240X2", "30240X3", "30240X4", "30240Y0", "30240Y2", "30240Y3", "30240Y4", "30243AZ", "30243G0", "30243G2", "30243G3", "30243G4", "30243X0", "30243X2", "30243X3", "30243X4", "30243Y0", "30243Y2", "30243Y3", "30243Y4", "30250G0", "30250G1", "30250X0", "30250X1", "30250Y0", "30250Y1", "30253G0", "30253G1", "30253X0", "30253X1", "30253Y0", "30253Y1", "30260G0", "30260G1", "30260X0", "30260X1", "30260Y0", "30260Y1", "30263G0", "30263G1", "30263X0", "30263X1", "30263Y0", "30263Y1", "3E03005", "3E030U0", "3E030U1", "3E03305", "3E033U0", "3E033U1", "3E04005", "3E04305", "3E05005", "3E05305", "3E06005", "3E06305", "3E0J3U0", "3E0J3U1", "3E0J7U0", "3E0J7U1", "3E0J8U0", "3E0J8U1" }

**Study outcome:**

**Use of corticosteroid (distinguished by routes: ORAL vs. PARENTERAL ["INJECTION", "INTRAMUSCULAR", "INTRAVENOUS"])**

The primary outcome was defined as having a dispensing record or a procedure code indicating use of systemic steroids orally, intravenously, or intramuscularly within 7 days of cohort entry date. See Appendix for details of the study outcome definitions. Follow-up began on the cohort entry date and continued until first of: occurrence of outcome event, disenrollment from the insurance or drug coverage plan, death, hospitalization or nursing home admission (Truven only has outpatient dispending records that do not capture medication use in the hospital or skilled nursing facility), or 7 days after cohort entry date.

- - **NDC Code** is any of: { "00009001201", "00009002001", "00009002201", "00009003101", "00009004401", "00009004902", "00009005602", "00009005604", "00009007301", "00009017601", "00054001720", "00054001725", "00054001729", "00054001820", "00054001825", "00054001829", "00054001920", "00054001925", "00054317644", "00054317757", "00054317763", "00054372144", "00054372250", "00054372263", "00054417925", "00054418025", "00054418125", "00054418225", "00054418325", "00054418425", "00054418625", "00054472825", "00054472831", "00054474125", "00054474131", "00054474225", "00054817425", "00054817525", "00054817625", "00054817925", "00054818025", "00054818125", "00054818325", "00054872216", "00054872425", "00054873925", "00054874025", "00085094205", "00093611816", "00093611887", "00095008735", "00095008851", "00095008921", "00121075908", "00143120201", "00143125401", "00143142501", "00143147301", "00143147310", "00143147501", "00143147510", "00143147701", "00143147705", "00143147710", "00143973801", "00143973805", "00143973810", "00143973901", "00143973910", "00143974001", "00143974010", "00178058208", "00186070210", "00378715501", "00378715505", "00440216512", "00440216715", "00440216720", "00555030102", "00591505201", "00591505210", "00591544201", "00591544205", "00591544210", "00591544301", "00591544305", "00591544310", "00603114756", "00603156756", "00603156758", "00603389919", "00603390021", "00603390121", "00603459315", "00603459321", "00603533521", "00603533532", "00603533621", "00603533715", "00603533721", "00603533731", "00603533732", "00603533815", "00603533821", "00603533828", "00603533831", "00603533832", "00603533921", "00603533928", "00603533932", "00603938856", "00781502201", "00781502207", "00904267460", "10544032221", "13925050104", "16477050501", "16477050521", "16477050548", "16477050748", "16477051008", "16590014921", "16590026910", "16590032610", "16590032615", "16590032620", "16590032621", "16590032630", "16590032645", "16590032660", "16590036521", "16590036548", "16590037321", "16590037328", "16590037330", "16590040420", "16590040421", "16590040430", "16590040440", "16590040445", "16590040448", "16590062421", "16590062448", "17856075905", "21695008021", "21695029030", "21695030521", "21695030530", "21695030590", "21695030620", "21695030621", "21695030628", "21695030630", "21695030636", "21695030639", "21695030640", "21695030642", "21695030645", "21695030648", "21695030650", "21695030690", "21695030705", "21695030706", "21695030707", "21695030709", "21695030710", "21695030712", "21695030713", "21695030714", "21695030715", "21695030718", "21695030720", "21695030721", "21695030730", "21695030790", "21695036508", "21695036516", "21695038204", "21695038206", "21695038208", "21695038220", "21695038260", "21695040508", "21695058005", "21695058007", "21695058014", "21695072812", "21695074510", "21695074512", "21695076421", "21695076521", "21695076548", "23490540401", "23490540701", "23490540702", "23490590201", "23490614401", "23490614402", "23490614403", "23490614501", "23490614502", "23490614503", "23490615701", "23490615702", "23490615703", "23490615704", "23490615705", "23490615706", "23490615707", "23490615708", "23490615800", "23490615801", "23490615802", "23490615803", "23490615804", "23490615805", "23490615807", "23490615808", "23490615809", "23490615901", "23490615902", "23490615903", "23490615904", "23490615905", "23490615906", "23490785400", "23589006793", "23589007093", "33358024121", "33358029108", "33358029212", "33358029215", "33358029221", "33358029230", "33358029278", "33358029320", "33358029330", "33358029340", "33358029415", "33358029420", "33358029430", "33358029440", "33358029460", "35356019421", "35356035930", "35356048804", "35356058130", "35356067320", "35356067321", "35356067330", "35356067415", "35356067418", "35356067420", "35356067430", "35356067560", "35356067648", "35356067715", "35356067720", "35356067721", "35356067730", "35356067740", "35356076321", "35356081810", "35356081814", "35356081815", "35356081818", "35356081820", "35356081821", "35356081830", "35356081915", "35356081920", "35356081921", "35356081930", "35356081940", "35356081942", "38739015008", "42254010208", "42254024021", "42254027621", "42254036108", "42254036114", "42254036115", "42254036120", "42254036121", "42254036128", "42254036130", "42254036136", "42254036140", "42254036142", "42254036145", "42254036148", "42549052221", "42549064714", "43063009703", "43063009706", "43063010910", "43063020801", "43063020830", "43063020860", "43063026607", "43063038630", "43063041501", "43063041530", "43063042610", "43063042620", "43063042621", "43063042630", "43063042640", "43063042642", "43063042650", "43063042660", "43063043210", "43063043212", "43063043215", "43063043220", "43063043221", "43063043230", "43353008060", "43353065760", "43353081960", "44183050735", "44183050851", "44183050921", "45802030321", "45802030367", "45802073321", "45802073367", "49884008401", "49884008501", "49884008601", "49884008701", "49884012901", "49884050101", "49999000800", "49999000805", "49999000820", "49999000821", "49999000830", "49999000840", "49999000842", "49999000855", "49999002805", "49999002812", "49999002814", "49999002815", "49999002820", "49999002821", "49999002828", "49999002830", "49999002840", "49999002848", "49999002850", "49999002860", "49999002890", "49999005906", "49999005930", "49999011000", "49999011006", "49999011007", "49999011010", "49999011012", "49999011014", "49999011015", "49999011018", "49999011020", "49999011021", "49999011030", "49999015321", "49999015330", "49999033508", "49999033524", "49999043703", "49999092901", "50383004004", "50383004224", "50383004248", "50436403701", "51079002001", "51079002003", "51138014430", "51138014515", "51138014520", "51138014530", "51138014615", "51138014620", "51138014621", "51138014628", "51138014636", "51138014639", "51138014642", "51138014650", "51138014710", "51138014712", "51138014714", "51138014715", "51138014720", "51138014721", "51138014730", "51138015415", "51138015420", "51138015430", "51138015515", "51138015520", "51138015521", "51138015528", "51138015536", "51138015539", "51138015542", "51138015550", "51138015610", "51138015612", "51138015614", "51138015615", "51138015620", "51138015621", "51138015630", "51672133803", "51991018831", "51991045801", "52959010000", "52959012600", "52959012605", "52959012607", "52959012610", "52959012612", "52959012615", "52959012618", "52959012620", "52959012621", "52959012625", "52959012630", "52959012640", "52959012642", "52959012644", "52959012650", "52959012660", "52959012670", "52959012700", "52959012707", "52959012710", "52959012712", "52959012715", "52959012718", "52959012720", "52959012721", "52959012725", "52959012730", "52959012737", "52959012742", "52959022000", "52959022010", "52959022020", "52959022021", "52959022030", "52959022036", "52959022040", "52959022060", "52959022075", "52959039212", "52959039228", "52959039230", "52959054710", "52959054711", "52959054712", "52959054716", "52959054720", "52959054730", "52959054750", "52959062260", "52959095405", "53014025001", "53489013905", "53489013910", "54505033105", "54505033210", "54505033310", "54569032200", "54569032203", "54569032404", "54569032700", "54569033000", "54569033001", "54569033003", "54569033004", "54569033007", "54569033100", "54569033101", "54569033102", "54569033104", "54569033105", "54569033107", "54569033108", "54569033201", "54569033202", "54569033203", "54569033205", "54569033209", "54569033300", "54569033601", "54569103600", "54569133500", "54569304300", "54569304301", "54569304302", "54569304305", "54569304306", "54569330200", "54569330201", "54569341300", "54569402604", "54569482700", "54569482701", "54569572900", "54569574900", "54569574901", "54868021800", "54868021801", "54868021802", "54868021803", "54868021804", "54868021805", "54868021806", "54868021807", "54868021808", "54868021809", "54868025801", "54868025802", "54868025804", "54868025805", "54868025806", "54868025808", "54868025809", "54868077601", "54868082100", "54868083600", "54868083602", "54868083603", "54868083604", "54868083605", "54868083607", "54868083608", "54868090800", "54868090801", "54868090802", "54868090803", "54868090804", "54868090805", "54868091600", "54868092700", "54868095400", "54868111901", "54868111902", "54868111903", "54868111904", "54868111905", "54868118300", "54868118301", "54868118302", "54868118303", "54868118304", "54868118307", "54868118308", "54868118309", "54868172000", "54868174300", "54868174301", "54868174302", "54868174303", "54868174304", "54868174400", "54868291300", "54868291301", "54868291302", "54868291303", "54868315701", "54868409500", "54868409600", "54868474800", "54868474900", "54868474901", "54868491000", "54868491001", "54868491002", "54868491003", "54868495200", "54868495201", "54868521300", "54868523000", "54868524200", "54868533400", "54868590300", "54868662401", "54879000308", "55045125909", "55045126009", "55045296301", "55289033005", "55289033007", "55289033010", "55289035205", "55289035207", "55289035209", "55289035210", "55289035212", "55289035214", "55289035215", "55289035220", "55289035221", "55289035230", "55289037301", "55289037321", "55289037330", "55289037336", "55289037342", "55289037346", "55289037355", "55289037360", "55289037372", "55289043815", "55289043820", "55289043821", "55289043830", "55289043836", "55289043838", "55289043840", "55289043842", "55289043850", "55289043860", "55289058204", "55289058206", "55289058210", "55289058228", "55289064930", "55289064998", "55289090310", "55289090312", "55289090320", "58016021600", "58016021612", "58016021614", "58016021615", "58016021620", "58016021621", "58016021624", "58016021628", "58016021630", "58016021632", "58016021640", "58016021650", "58016021660", "58016021690", "58016021700", "58016021710", "58016021715", "58016021716", "58016021718", "58016021720", "58016021721", "58016021722", "58016021724", "58016021728", "58016021730", "58016021740", "58016021760", "58016021800", "58016021820", "58016021821", "58016021824", "58016021830", "58016021833", "58016021836", "58016021840", "58016021850", "58016021855", "58016021860", "58016021890", "58016029000", "58016029002", "58016029003", "58016029012", "58016029015", "58016029020", "58016029030", "58016029073", "58016029089", "58016029300", "58016029312", "58016029315", "58016029320", "58016029330", "58016078100", "58016078110", "58016078112", "58016078114", "58016078115", "58016078120", "58016078121", "58016078124", "58016078128", "58016078130", "58016078140", "58016078150", "58016200101", "58016200401", "58016414401", "58016471901", "58016483201", "58016484301", "58864036220", "58864036256", "58864042315", "58864042320", "58864042330", "58864042340", "58864042414", "58864042420", "58864042430", "59630070048", "59630070148", "59630070248", "59630071008", "59630071010", "59746000103", "59746000106", "59746000204", "59746000314", "59746001504", "59746017106", "59746017110", "59746017206", "59746017210", "59746017306", "59746017309", "59746017310", "59746017506", "59746017509", "59746017510", "59762004901", "59762005001", "59762005101", "59762007301", "59762007401", "59762007501", "59762444002", "59762444003", "60429001501", "60429013001", "60429013010", "60429013101", "60429013110", "60429013201", "60429013210", "60429026201", "60432021208", "60432046608", "60760000221", "60760062921", "63629157900", "63629157901", "63629157902", "63629157903", "63629157904", "63629157905", "63629157906", "63629157907", "63629157908", "63629157909", "63629158701", "63629158702", "63629158703", "63629158704", "63629158705", "63629158706", "63629158707", "63629158708", "63629160501", "63629160502", "63629160503", "63629160504", "63629160505", "63629160506", "63629160507", "63629160508", "63629186201", "63629374201", "63629374202", "63629374203", "63629391001", "63629412701", "63629412901", "63629462401", "63717091508", "63739016110", "63739020710", "63739020810", "63739020910", "63739051810", "63739051910", "63739052010", "64679081008", "64720033105", "64720033210", "64720033310", "64980050924", "65162066788", "65162066790", "65483070210", "65580025101", "66267006612", "66267006630", "66267006704", "66267006708", "66267006710", "66267006712", "66267006720", "66267006721", "66267017115", "66267017120", "66267017121", "66267017130", "66267017140", "66267017142", "66267017210", "66267017212", "66267017215", "66267017220", "66267017230", "66267017242", "66267017320", "66267017330", "66267017340", "66267017342", "66267017360", "66267086004", "66267094821", "66267096121", "66336005812", "66336005821", "66336005830", "66336009410", "66336009412", "66336009418", "66336009420", "66336009421", "66336009430", "66336021930", "66336047906", "66336047915", "66336047944", "66336055021", "67263028201", "67263034301", "67263040086", "67544039960", "68001000501", "68012030930", "68071153208", "68084014901", "68084014911", "68084022411", "68084022421", "68084046901", "68084046911", "68258898702", "68387017001", "68387017221", "68387024010", "68387024025", "68387024115", "68791010004", "68850000108", "75987002001", "75987002101", "75987002201", "00003029305", "00003029320", "00003029328", "00003049420", "00009000302", "00009000501", "00009001103", "00009001104", "00009001305", "00009001306", "00009001612", "00009001820", "00009003928", "00009003930", "00009003932", "00009003933", "00009004722", "00009004725", "00009004726", "00009004727", "00009019009", "00009027401", "00009028002", "00009028003", "00009028051", "00009028052", "00009030602", "00009030612", "00009069801", "00009075801", "00009079601", "00009082501", "00009307301", "00009307303", "00009307322", "00009307323", "00009347501", "00009347503", "00009347522", "00009347523", "00069017701", "00069017702", "00069017801", "00069017802", "00069017901", "00069017902", "00069019201", "00069019202", "00069454101", "00069454102", "00069454301", "00069454302", "00069454501", "00069454502", "00069454701", "00069454702", "00085056605", "00409485605", "00409568423", "00409568523", "00517072001", "00517490125", "00517490525", "00517493025", "00641036721", "00641036725", "00703003101", "00703003104", "00703004301", "00703004501", "00703005101", "00703005104", "00703006301", "00781308475", "00781308571", "00781308575", "00781313171", "00781313195", "00781313271", "00781313295", "00781313670", "00781313775", "21695036001", "21695036010", "21695058710", "21695084830", "21695084910", "21695085005", "21695095201", "21695095205", "21695095210", "35356008201", "35356008301", "35356008401", "35356017805", "35356048310", "35356048405", "49999041505", "49999043425", "54569302701", "54868020600", "54868023400", "54868023500", "54868023501", "54868023502", "54868059000", "54868087100", "54868087106", "54868118500", "54868118501", "54868334400", "54868389600", "54868389601", "54868389602", "54868609900", "54868619900", "55045324205", "55045350901", "55390020910", "55390021010", "55390025801", "55390025901", "58016485501", "58016489301", "58016919101", "58016979901", "58016993401", "63323016501", "63323016505", "63323016530", "63323025503", "63323025803", "63323026530", "63323050601", "63323051610", "66647714500", "66647715700", "68258889805", "68258890305", "76420052401" }

**Generic Name** is any of: { "CORTISONE ACETATE", "DEXAMETHASONE", "HYDROCORTISONE", "METHYLPREDNISOLONE", "PREDNISOLONE", "PREDNISONE", "TRIAMCINOLONE", "TRIAMCINOLONE DIACETATE", "BETAMETHASONE" }

**Brand Name** is any of: { "A-HYDROCORT", "A-METHAPRED", "ARISTOSPAN", "ARISTOSPAN PARENTERAL", "DEPO-MEDROL", "EZ USE JOINT-TUNNEL-TRIGGER", "PHYSICIANS EZ USE B-12", "PHYSICIANS EZ USE FLU 2012-13", "SOLU-CORTEF", "SOLU-MEDROL", "SOLU-MEDROL MIX-O-VIAL", "SOLU-MEDROL W/ADMIN SET", "SOLU-MEDROL W/DILUENT", "CORTEF", "MEDROL", "ARISTOCORT", "ARISTOCORT A", "ARISTOCORT FORTE", "ARISTOCORT HP", "ARISTOCORT LP", "ARISTOCORT R", "ASMALPRED", "ASMALPRED PLUS", "BAYCADRON", "DE-SONE", "DE-SONE LA", "DECADRON", "DECADRON PHOSPHATE", "DECADRON TURBINAIRE", "DECADRON-LA", "DELTASONE", "DEP-MEDALONE 40", "DEP-MEDALONE 80", "DEPOPRED-40", "DEPOPRED-80", "DEXACEN LA-8", "DEXACEN-4", "DEXACORT PHOSPHATE TURBINAIRE", "DEXACORTEN", "DEXACORTEN-LA", "DEXASONE", "DEXASONE 10", "DEXASONE 4", "DEXASONE LA", "DEXPAK", "DURALONE", "DURALONE-40", "DURALONE-80", "FLO-PRED", "METHYLPREDNISONE", "MILLIPRED", "MILLIPRED DP", "NEODECADRON", "ORAPRED", "ORAPRED ODT", "PEDIAPRED", "PREDNISONE", "PREDNISONE INTENSOL", "PREDNISONE MICRONIZED", "PREDNISONE U.S.P.", "PREDNISONE-5", "PRELONE", "RAYOS", "SK-PREDNISONE", "STERAPRED", "STERAPRED DS", "UCERIS", "ZEMA-PAK"}

- - **Procedure Code (Any Position), CPT and HCPC** is any of: { "J7506", "J7509", "J7510", "J8540", "J1020", "J1030", "J1040", "J1720", "J2920", "J2930", "J3303", "J0702", "J3301", "J3300", "J3302", "J1100", "J1094" }

**Study covariates:**

The baseline assessment period of the study covariates is from 365 days prior to 1 day prior to the cohort entry date.

**Stroke (ICD 9&10) At least 1 inpatient or outpatient code for**: { "430", "431", "433.0", "433.00", "433.01", "433.1", "433.10", "433.11", "433.2", "433.20", "433.21", "433.3", "433.30", "433.31", "433.8", "433.80", "433.81", "433.9", "433.90", "433.91", "434.0", "434.00", "434.01", "434.1", "434.10", "434.11", "434.9", "434.90", "434.91", "436", "I60.00", "I60.01", "I60.02", "I60.10", "I60.11", "I60.12", "I60.2", "I60.30", "I60.31", "I60.32", "I60.4", "I60.50", "I60.51", "I60.52", "I60.6", "I60.7", "I60.8", "I60.9", "I61.0", "I61.1", "I61.2", "I61.3", "I61.4", "I61.5", "I61.6", "I61.8", "I61.9", "I63.00", "I63.011", "I63.012", "I63.019", "I63.02", "I63.031", "I63.032", "I63.039", "I63.09", "I63.10", "I63.111", "I63.112", "I63.119", "I63.12", "I63.131", "I63.132", "I63.139", "I63.19", "I63.20", "I63.211", "I63.212", "I63.219", "I63.22", "I63.231", "I63.232", "I63.239", "I63.29", "I63.30", "I63.311", "I63.312", "I63.319", "I63.321", "I63.322", "I63.329", "I63.331", "I63.332", "I63.339", "I63.341", "I63.342", "I63.349", "I63.39", "I63.40", "I63.411", "I63.412", "I63.419", "I63.421", "I63.422", "I63.429", "I63.431", "I63.432", "I63.439", "I63.441", "I63.442", "I63.449", "I63.49", "I63.50", "I63.511", "I63.512", "I63.519", "I63.521", "I63.522", "I63.529", "I63.531", "I63.532", "I63.539", "I63.541", "I63.542", "I63.549", "I63.59", "I63.6", "I63.8", "I63.9", "I65.01", "I65.02", "I65.03", "I65.09", "I65.1", "I65.21", "I65.22", "I65.23", "I65.29", "I65.8", "I65.9", "I66.01", "I66.02", "I66.03", "I66.09", "I66.11", "I66.12", "I66.13", "I66.19", "I66.21", "I66.22", "I66.23", "I66.29", "I66.3", "I66.8", "I66.9" }

**Hypertension (ICD 9&10): At least 1 inpatient or outpatient code for**: { "401", "401.0", "401.1", "401.9", "402", "402.0", "402.00", "402.01", "402.1", "402.10", "402.11", "402.9", "402.90", "402.91", "403", "403.0", "403.00", "403.01", "403.1", "403.10", "403.11", "403.9", "403.90", "403.91", "404", "404.0", "404.00", "404.01", "404.02", "404.03", "404.1", "404.10", "404.11", "404.12", "404.13", "404.9", "404.90", "404.91", "404.92", "404.93", "405", "405.0", "405.01", "405.09", "405.1", "405.11", "405.19", "405.9", "405.91", "405.99", "I10", "I11.0", "I11.9", "I12.0", "I12.9", "I13.0", "I13.10", "I13.11", "I13.2", "I15.0", "I15.1", "I15.2", "I15.8", "I15.9", "N26.2" }

**Atrial fibrillation (ICD9&10): At least 1 inpatient or outpatient code for**: { "427.3", "427.31", "427.32", "I48.0", "I48.1", "I48.2", "I48.3", "I48.4", "I48.91", "I48.92" }

**Chronic renal insufficiency (ICD9&10): At least 1 inpatient or outpatient code for**: { "582", "582.0", "582.1", "582.2", "582.4", "582.8", "582.81", "582.89", "582.9", "583", "583.0", "583.1", "583.2", "583.4", "583.6", "583.7", "583.8", "583.81", "583.89", "583.9", "585", "585.1", "585.2", "585.3", "585.4", "585.5", "585.6", "585.9", "586", "587", "E092.1", "E092.2", "E092.9", "M32.14", "M32.15", "M35.04", "N03.0", "N03.1", "N03.2", "N03.3", "N03.4", "N03.5", "N03.6", "N03.7", "N03.8", "N03.9", "N05.0", "N05.1", "N05.2", "N05.3", "N05.4", "N05.5", "N05.6", "N05.7", "N05.8", "N05.9", "N06.0", "N06.1", "N06.2", "N06.3", "N06.4", "N06.5", "N06.6", "N06.7", "N06.8", "N06.9", "N07.0", "N07.1", "N07.2", "N07.3", "N07.4", "N07.5", "N07.6", "N07.7", "N07.8", "N07.9", "N08", "N14.0", "N14.1", "N14.2", "N14.3", "N14.4", "N15.0", "N15.8", "N15.9", "N16", "N17.1", "N17.2", "N18.1", "N18.2", "N18.3", "N18.4", "N18.5", "N18.6", "N18.9", "N19", "N26.1", "N26.9" }

**DM Type 2 MS (ICD9&10): At least 1 inpatient or outpatient code for:** { "250.00", "250.02", "250.10", "250.12", "250.20", "250.22", "250.30", "250.32", "250.40", "250.42", "250.50", "250.52", "250.60", "250.62", "250.70", "250.72", "250.80", "250.82", "250.90", "250.92", "E11", "E11.0", "E11.00", "E11.01", "E11.2", "E11.21", "E11.22", "E11.29", "E11.3", "E11.31", "E11.311", "E11.319", "E11.32", "E11.321", "E11.329", "E11.33", "E11.331", "E11.339", "E11.34", "E11.341", "E11.349", "E11.35", "E11.351", "E11.359", "E11.36", "E11.39", "E11.4", "E11.40", "E11.41", "E11.42", "E11.43", "E11.44", "E11.49", "E11.5", "E11.51", "E11.52", "E11.59", "E11.6", "E11.61", "E11.610", "E11.618", "E11.62", "E11.620", "E11.621", "E11.622", "E11.628", "E11.63", "E11.630", "E11.638", "E11.64", "E11.641", "E11.649", "E11.65", "E11.8", "E11.9" }

**DM Type 1 (ICD9&10): At least 1 inpatient or outpatient code for**: { "250.01", "250.03", "250.11", "250.13", "250.21", "250.23", "250.31", "250.33", "250.41", "250.43", "250.51", "250.53", "250.61", "250.63", "250.71", "250.73", "250.81", "250.83", "250.91", "250.93", "E10", "E101", "E101.0", "E101.1", "E102", "E102.1", "E102.2", "E102.9", "E103", "E103.1", "E103.11", "E103.19", "E103.2", "E103.21", "E103.29", "E103.3", "E103.31", "E103.39", "E103.4", "E103.41", "E103.49", "E103.5", "E103.51", "E103.59", "E103.6", "E103.9", "E104", "E104.0", "E104.1", "E104.2", "E104.3", "E104.4", "E104.9", "E105", "E105.1", "E105.2", "E105.9", "E106", "E106.1", "E106.10", "E106.18", "E106.2", "E106.20", "E106.21", "E106.22", "E106.28", "E106.3", "E106.30", "E106.38", "E106.4", "E106.41", "E106.49", "E106.5", "E106.9", "E108", "E109" }

**Dementia (ICD9&10): At least 1 inpatient or outpatient code for**: { “290.0”, “290.1”, “290.10”, “290.11”, “290.12”, “290.13”, “290.2”, “290.20”, “290.21”, “290.3”, “290.4”, “290.40”, “290.41”, “290.42”, “290.43”, “290.8”, “290.9”, “294.0”, “294.1”, “294.10”, “294.11”, “294.2”, “294.20”, “294.21”, “294.8”, “294.9”, “330.0”, “330.1”, “330.2”, “330.3”, “331.0”, “331.1”, “331.11”, “331.19”, “331.2”, “331.3”, “331.4”, “331.5”, “331.6”, “331.7”, “331.8”, “331.81”, “331.82”, “331.83”, “331.89”, “331.9”, “E750.0”, “E750.1”, “E750.2”, “E750.9”, “E751.0”, “E751.1”, “E751.9”, “E752.3”, “E752.5”, “E752.9”, “E754”, “F01.50”, “F01.51”, “F02.80”, “F02.81”, “F03.90”, “F03.91”, “F04”, “F05”, “F06.0”, “F06.1”, “F06.8”, “F84.2”, “G13.2”, “G13.8”, “G30.0”, “G30.1”, “G30.8”, “G30.9”, “G31.01”, “G31.09”, “G31.1”, “G31.2”, “G31.81”, “G31.82”, “G31.83”, “G31.84”, “G31.85”, “G31.89”, “G31.9”, “G91.0”, “G91.1”, “G91.2”, “G91.3”, “G91.4”, “G91.8”, “G91.9”, “G93.7”, “G93.89”, “G93.9”, “G94”}

**Venous thromboembolism, (ICD9&10): At least 1 inpatient or outpatient code for:** : { “415.1”, “451.11”, “451.2”, “451.81”, “451.9”, “453.1”, “453.2”, “453.8”, “453.9”, “453.82”, “453.83”, “453.84”, “453.85”, “453.86”, “453.87”, “453.89”, “I80.10”, “I80.219”, “I80.3”, “I80.9”, “I82.1”, “I82.220”, “I82.221”, “I82.91”, “I82.629”, “I82.609”, “I82.A19”, “I82.B19”, “I82.C19”, “I82.290”, “I82.890”}

**Falls (ICD9&10): At least 1 inpatient or outpatient code for**: { "E880", "E880.0", "E880.1", "E880.9", "E881", "E881.0", "E881.1", "E882", "E883", "E883.0", "E883.1", "E883.2", "E883.9", "E884", "E884.0", "E884.1", "E884.2", "E884.3", "E884.4", "E884.5", "E884.6", "E884.9", "E885", "E885.0", "E885.1", "E885.2", "E885.3", "E885.4", "E885.9", "E886", "E886.0", "E886.9", "E887", "E888", "E888.0", "E888.1", "E888.8", "E888.9", "V15.88", "V00.111A", "V00.111D", "V00.112A", "V00.112D", "V00.118A", "V00.118D", "V00.121A", "V00.121D", "V00.122A", "V00.122D", "V00.128A", "V00.128D", "V00.131A", "V00.131D", "V00.132A", "V00.132D", "V00.138A", "V00.138D", "V00.141A", "V00.141D", "V00.142A", "V00.142D", "V00.148A", "V00.148D", "V00.151A", "V00.151D", "V00.152A", "V00.152D", "V00.158A", "V00.158D", "V00.181A", "V00.181D", "V00.182A", "V00.182D", "V00.188A", "V00.188D", "V00.211A", "V00.211D", "V00.212A", "V00.212D", "V00.218A", "V00.218D", "V00.221A", "V00.221D", "V00.222A", "V00.222D", "V00.228A", "V00.228D", "V00.281A", "V00.281D", "V00.282A", "V00.282D", "V00.288A", "V00.288D", "V00.311A", "V00.311D", "V00.312A", "V00.312D", "V00.318A", "V00.318D", "V00.321A", "V00.321D", "V00.322A", "V00.322D", "V00.328A", "V00.328D", "V00.381A", "V00.381D", "V00.382A", "V00.382D", "V00.388A", "V00.388D", "V00.811A", "V00.811D", "V00.812A", "V00.812D", "V00.818A", "V00.818D", "V00.821A", "V00.821D", "V00.822A", "V00.822D", "V00.828A", "V00.828D", "V00.831A", "V00.831D", "V00.832A", "V00.832D", "V00.838A", "V00.838D", "V00.891A", "V00.891D", "V00.892A", "V00.892D", "V00.898A", "V00.898D", "W00.0XXA", "W00.0XXD", "W00.1XXA", "W00.1XXD", "W00.2XXA", "W00.2XXD", "W00.9XXA", "W00.9XXD", "W01.0XXA", "W01.0XXD", "W01.10XA", "W01.10XD", "W01.110A", "W01.110D", "W01.111A", "W01.111D", "W01.118A", "W01.118D", "W01.119A", "W01.119D", "W01.190A", "W01.190D", "W01.198A", "W01.198D", "W03.XXXA", "W03.XXXD", "W04.XXXA", "W04.XXXD", "W05.0XXA", "W05.0XXD", "W05.1XXA", "W05.1XXD", "W05.2XXA", "W05.2XXD", "W06.XXXA", "W06.XXXD", "W07.XXXA", "W07.XXXD", "W08.XXXA", "W08.XXXD", "W09.0XXA", "W09.0XXD", "W09.1XXA", "W09.1XXD", "W09.2XXA", "W09.2XXD", "W09.8XXA", "W09.8XXD", "W10.0XXA", "W10.0XXD", "W10.1XXA", "W10.1XXD", "W10.2XXA", "W10.2XXD", "W10.8XXA", "W10.8XXD", "W10.9XXA", "W10.9XXD", "W11.XXXA", "W11.XXXD", "W12.XXXA", "W12.XXXD", "W13.0XXA", "W13.0XXD", "W13.1XXA", "W13.1XXD", "W13.2XXA", "W13.2XXD", "W13.3XXA", "W13.3XXD", "W13.4XXA", "W13.4XXD", "W13.8XXA", "W13.8XXD", "W13.9XXA", "W13.9XXD", "W14.XXXA", "W14.XXXD", "W15.XXXA", "W15.XXXD", "W16.011A", "W16.011D", "W16.012A", "W16.012D", "W16.021A", "W16.021D", "W16.022A", "W16.022D", "W16.031A", "W16.031D", "W16.032A", "W16.032D", "W16.111A", "W16.111D", "W16.112A", "W16.112D", "W16.121A", "W16.121D", "W16.122A", "W16.122D", "W16.131A", "W16.131D", "W16.132A", "W16.132D", "W16.211A", "W16.211D", "W16.212A", "W16.212D", "W16.221A", "W16.221D", "W16.222A", "W16.222D", "W16.311A", "W16.311D", "W16.312A", "W16.312D", "W16.321A", "W16.321D", "W16.322A", "W16.322D", "W16.331A", "W16.331D", "W16.332A", "W16.332D", "W16.41XA", "W16.41XD", "W16.42XA", "W16.42XD", "W16.511A", "W16.511D", "W16.512A", "W16.512D", "W16.521A", "W16.521D", "W16.522A", "W16.522D", "W16.531A", "W16.531D", "W16.532A", "W16.532D", "W16.611A", "W16.611D", "W16.612A", "W16.612D", "W16.621A", "W16.621D", "W16.622A", "W16.622D", "W16.711A", "W16.711D", "W16.712A", "W16.712D", "W16.721A", "W16.721D", "W16.722A", "W16.722D", "W16.811A", "W16.811D", "W16.812A", "W16.812D", "W16.821A", "W16.821D", "W16.822A", "W16.822D", "W16.831A", "W16.831D", "W16.832A", "W16.832D", "W16.91XA", "W16.91XD", "W16.92XA", "W16.92XD", "W17.0XXA", "W17.0XXD", "W17.1XXA", "W17.1XXD", "W17.2XXA", "W17.2XXD", "W17.3XXA", "W17.3XXD", "W17.4XXA", "W17.4XXD", "W17.81XA", "W17.81XD", "W17.82XA", "W17.82XD", "W17.89XA", "W17.89XD", "W18.00XA", "W18.00XD", "W18.01XA", "W18.01XD", "W18.02XA", "W18.02XD", "W18.09XD", "W18.11XA", "W18.11XD", "W18.12XA", "W18.12XD", "W18.2XXA", "W18.2XXD", "W18.30XA", "W18.30XD", "W18.31XA", "W18.31XD", "W18.39XA", "W18.39XD", "W18.40XA", "W18.40XD", "W18.41XA", "W18.41XD", "W18.42XA", "W18.42XD", "W18.43XA", "W18.43XD", "W18.49XA", "W18.49XD", "W19.XXXA", "W19.XXXD", "Z91.81" }

**Heart failure (ICD9&10): At least 1 inpatient or outpatient code for**: { “398.91”, “402.01”, “402.11”, “402.91”, “404.01”, “404.03”, “404.11”, “404.13”, “404.93”, “428.0”, “428.1”, “428.2”, “428.20”, “428.21”, “428.22”, “428.23”, “428.3”, “428.30”, “428.31”, “428.32”, “428.33”, “428.4”, “428.40”, “428.41”, “428.42”, “428.43”, “428.9”, “428”, “I09.81”, “I11.0”, “I13.0”, “I13.2”, “I50.1”, “I50.20”, “I50.21”, “I50.22”, “I50.23”, “I50.30”, “I50.31”, “I50.32”, “I50.33”, “I50.40”, “I50.41”, “I50.42”, “I50.43”, “I50.9”}

**IBD (ICD 9&10): At least 1 inpatient or outpatient code for**: { "555", "555.0", "555.1", "555.2", "555.9", "556", "556.0", "556.1", "556.2", "556.3", "556.5", "556.6", "556.8", "556.9", "K50", "K50.0", "K50.00", "K50.01", "K50.011", "K50.012", "K50.013", "K50.014", "K50.018", "K50.019", "K50.1", "K50.10", "K50.11", "K50.111", "K50.112", "K50.113", "K50.114", "K50.118", "K50.119", "K50.8", "K50.80", "K50.81", "K50.811", "K50.812", "K50.813", "K50.814", "K50.818", "K50.819", "K50.9", "K50.90", "K50.91", "K50.911", "K50.912", "K50.913", "K50.914", "K50.918", "K50.919", "K51.0", "K51.00", "K51.01", "K51.011", "K51.012", "K51.013", "K51.014", "K51.018", "K51.019", "K51.2", "K51.20", "K51.21", "K51.211", "K51.212", "K51.213", "K51.214", "K51.218", "K51.219", "K51.3", "K51.30", "K51.31", "K51.311", "K51.312", "K51.313", "K51.314", "K51.318", "K51.319", "K51.8", "K51.80", "K51.81", "K51.811", "K51.812", "K51.813", "K51.814", "K51.818", "K51.819", "K51.9", "K51.90", "K51.91", "K51.911", "K51.912", "K51.913", "K51.914", "K51.918", "K51.919" }

**Ischemic heart disease (ICD9&10): At least 1 inpatient or outpatient code for**: { "410.0", "410.00", "410.01", "410.02", "410.1", "410.10", "410.11", "410.12", "410.2", "410.20", "410.21", "410.22", "410.3", "410.30", "410.31", "410.32", "410.4", "410.40", "410.41", "410.42", "410.5", "410.50", "410.51", "410.52", "410.6", "410.60", "410.61", "410.62", "410.7", "410.70", "410.71", "410.72", "410.8", "410.80", "410.81", "410.82", "410.9", "410.90", "410.91", "410.92", "411.0", "411.1", "411.8", "411.81", "411.89", "412", "413.0", "413.1", "413.9", "414.0", "414.00", "414.01", "414.02", "414.03", "414.04", "414.05", "414.06", "414.07", "414.1", "414.10", "414.11", "414.12", "414.19", "414.2", "414.3", "414.4", "414.8", "414.9", "I20.0", "I20.1", "I20.8", "I20.9", "I21.01", "I21.02", "I21.09", "I21.11", "I21.19", "I21.21", "I21.29", "I21.3", "I21.4", "I22.0", "I22.1", "I22.2", "I22.8", "I22.9", "I24.0", "I24.1", "I24.8", "I24.9", "I25.10", "I25.110", "I25.111", "I25.118", "I25.119", "I25.2", "I25.3", "I25.41", "I25.42", "I25.5", "I25.6", "I25.700", "I25.701", "I25.708", "I25.709", "I25.710", "I25.711", "I25.718", "I25.719", "I25.720", "I25.721", "I25.728", "I25.729", "I25.730", "I25.731", "I25.738", "I25.739", "I25.750", "I25.751", "I25.758", "I25.759", "I25.760", "I25.761", "I25.768", "I25.769", "I25.790", "I25.791", "I25.798", "I25.799", "I25.810", "I25.811", "I25.812", "I25.82", "I25.83", "I25.84", "I25.89", "I25.9" }

**Liver disease (ICD9&10): At least 1 inpatient or outpatient code for**: { "070.0", "070.1", "070.2", "070.20", "070.21", "070.22", "070.23", "070.3", "070.30", "070.31", "070.32", "070.33", "070.4", "070.41", "070.42", "070.43", "070.44", "070.49", "070.5", "070.51", "070.52", "070.53", "070.54", "070.59", "070.6", "070.7", "070.70", "070.71", "070.9", "456.0", "456.1", "456.2", "456.20", "456.21", "570", "571.0", "571.1", "571.2", "571.3", "571.4", "571.40", "571.41", "571.42", "571.49", "571.5", "571.6", "571.8", "571.9", "572.0", "572.1", "572.2", "572.3", "572.4", "572.8", "573.0", "573.1", "573.2", "573.3", "573.4", "573.5", "573.8", "573.9", "576.8", "782.4", "789.5", "789.51", "789.59", "B15.0", "B15.9", "B16.0", "B16.1", "B16.2", "B16.9", "B17.0", "B17.10", "B17.11", "B17.2", "B17.8", "B17.9", "B18.0", "B18.1", "B18.2", "B18.8", "B18.9", "B19.0", "B19.10", "B19.11", "B19.20", "B19.21", "B19.9", "B25.1", "I85.00", "I85.01", "I85.10", "I85.11", "K70.0", "K70.10", "K70.11", "K70.2", "K70.30", "K70.31", "K70.40", "K70.41", "K70.9", "K71.0", "K71.10", "K71.11", "K71.2", "K71.3", "K71.4", "K71.50", "K71.51", "K71.6", "K71.7", "K71.8", "K71.9", "K72.00", "K72.01", "K72.10", "K72.11", "K72.90", "K72.91", "K73.0", "K73.1", "K73.2", "K73.8", "K73.9", "K74.0", "K74.1", "K74.2", "K74.3", "K74.4", "K74.5", "K74.60", "K74.69", "K75.0", "K75.1", "K75.2", "K75.3", "K75.4", "K75.81", "K75.89", "K75.9", "K76.0", "K76.1", "K76.2", "K76.3", "K76.4", "K76.5", "K76.6", "K76.7", "K76.81", "K76.89", "K76.9", "K77", "K83.5", "K83.8", "K87", "R17", "R18.0", "R18.8" }

**Obesity (ICD9&10): At least 1 inpatient or outpatient code for**: { "278.0", "278.00", "278.01", "278.03", "539", "539.0", "539.01", "539.09", "539.8", "539.81", "539.89", "649.1", "649.10", "649.11", "649.12", "649.13", "649.14", "649.2", "649.20", "649.21", "649.22", "649.23", "649.24", "V85.30", "V85.31", "V85.32", "V85.33", "V85.34", "V85.35", "V85.36", "V85.37", "V85.38", "V85.39", "V85.41", "V85.42", "V85.43", "V85.44", "V85.45", "E660.9", "E661", "E668", "E669", "O99.210", "O99.211", "O99.212", "O99.213", "O99.214", "O99.215", "O99.840", "O99.841", "O99.842", "O99.843", "O99.844", "O99.845", "Z68.30", "Z68.31", "Z68.32", "Z68.33", "Z68.34", "Z68.35", "Z68.36", "Z68.37", "Z68.38", "Z68.39", "Z68.41", "Z68.42", "Z68.43", "Z68.44", "Z68.45" }

**HIV, AIDS, (ICD9&10): At least 1 inpatient or outpatient code for:** : { "042", "795.71", "V08" }

**Bronchiectasis (ICD9&10): At least 1 inpatient or outpatient code for:** : { “011.5”, “011.50”, “011.51”, “011.52”, “011.53”, “011.54”, “011.55”, “011.56”, “494”, “494.0”, “494.1”, “748.61” “J47.1”, “J47.9”, “Q33.4”}

**Connective tissue diseases (ICD9&10): At least 1 inpatient or outpatient code for:** : { “267”, “607.85”, “696.0”, “710”, “710.0”, “710.1”, “710.2”, “710.3”, “710.4”, “710.5”, “710.8”, “710.9”, “714”, “714.0”, “714.1”, “714.2”, “714.3”, “714.30”, “714.31”, “714.32”, “714.33”, “714.4”, “714.8”, “714.81”, “714.89”, “714.9”, “756.51”, “756.83”, “E78.71”, “E78.72”, “L40.54”, “L40.59”, “M05.00”, “M05.10”, “M05.30”, “M05.60”, “M06.1”, “M06.4”, “M06.9”, “M08.00”, “M08.3”, “M08.40”, “M12.00”, “M32.10”, “M33.20”, “M33.90”, “M34.0”, “M34.1”, “M34.9”, “M35.00”, “M35.01”, “M35.5”, “M35.8”, “M35.9”, “N48.6”, “Q78.0”, “Q79.6”, “Q81.9”}

**Fracture (ICD9&10): At least 1 inpatient or outpatient code for:** : { "800", "800.0", "800.00", "800.01", "800.02", "800.03", "800.04", "800.05", "800.06", "800.09", "800.1", "800.10", "800.11", "800.12", "800.13", "800.14", "800.15", "800.16", "800.19", "800.2", "800.20", "800.21", "800.22", "800.23", "800.24", "800.25", "800.26", "800.29", "800.3", "800.30", "800.31", "800.32", "800.33", "800.34", "800.35", "800.36", "800.39", "800.4", "800.40", "800.41", "800.42", "800.43", "800.44", "800.45", "800.46", "800.49", "800.5", "800.50", "800.51", "800.52", "800.53", "800.54", "800.55", "800.56", "800.59", "800.6", "800.60", "800.61", "800.62", "800.63", "800.64", "800.65", "800.66", "800.69", "800.7", "800.70", "800.71", "800.72", "800.73", "800.74", "800.75", "800.76", "800.79", "800.8", "800.80", "800.81", "800.82", "800.83", "800.84", "800.85", "800.86", "800.89", "800.9", "800.90", "800.91", "800.92", "800.93", "800.94", "800.95", "800.96", "800.99", "801", "801.0", "801.00", "801.01", "801.02", "801.03", "801.04", "801.05", "801.06", "801.09", "801.1", "801.10", "801.11", "801.12", "801.13", "801.14", "801.15", "801.16", "801.19", "801.2", "801.20", "801.21", "801.22", "801.23", "801.24", "801.25", "801.26", "801.29", "801.3", "801.30", "801.31", "801.32", "801.33", "801.34", "801.35", "801.36", "801.39", "801.4", "801.40", "801.41", "801.42", "801.43", "801.44", "801.45", "801.46", "801.49", "801.5", "801.50", "801.51", "801.52", "801.53", "801.54", "801.55", "801.56", "801.59", "801.6", "801.60", "801.61", "801.62", "801.63", "801.64", "801.65", "801.66", "801.69", "801.7", "801.70", "801.71", "801.72", "801.73", "801.74", "801.75", "801.76", "801.79", "801.8", "801.80", "801.81", "801.82", "801.83", "801.84", "801.85", "801.86", "801.89", "801.9", "801.90", "801.91", "801.92", "801.93", "801.94", "801.95", "801.96", "801.99", "802", "802.0", "802.1", "802.2", "802.20", "802.21", "802.22", "802.23", "802.24", "802.25", "802.26", "802.27", "802.28", "802.29", "802.3", "802.30", "802.31", "802.32", "802.33", "802.34", "802.35", "802.36", "802.37", "802.38", "802.39", "802.4", "802.5", "802.6", "802.7", "802.8", "802.9", "803", "803.0", "803.00", "803.01", "803.02", "803.03", "803.04", "803.05", "803.06", "803.09", "803.1", "803.10", "803.11", "803.12", "803.13", "803.14", "803.15", "803.16", "803.19", "803.2", "803.20", "803.21", "803.22", "803.23", "803.24", "803.25", "803.26", "803.29", "803.3", "803.30", "803.31", "803.32", "803.33", "803.34", "803.35", "803.36", "803.39", "803.4", "803.40", "803.41", "803.42", "803.43", "803.44", "803.45", "803.46", "803.49", "803.5", "803.50", "803.51", "803.52", "803.53", "803.54", "803.55", "803.56", "803.59", "803.6", "803.60", "803.61", "803.62", "803.63", "803.64", "803.65", "803.66", "803.69", "803.7", "803.70", "803.71", "803.72", "803.73", "803.74", "803.75", "803.76", "803.79", "803.8", "803.80", "803.81", "803.82", "803.83", "803.84", "803.85", "803.86", "803.89", "803.9", "803.90", "803.91", "803.92", "803.93", "803.94", "803.95", "803.96", "803.99", "804", "804.0", "804.00", "804.01", "804.02", "804.03", "804.04", "804.05", "804.06", "804.09", "804.1", "804.10", "804.11", "804.12", "804.13", "804.14", "804.15", "804.16", "804.19", "804.2", "804.20", "804.21", "804.22", "804.23", "804.24", "804.25", "804.26", "804.29", "804.3", "804.30", "804.31", "804.32", "804.33", "804.34", "804.35", "804.36", "804.39", "804.4", "804.40", "804.41", "804.42", "804.43", "804.44", "804.45", "804.46", "804.49", "804.5", "804.50", "804.51", "804.52", "804.53", "804.54", "804.55", "804.56", "804.59", "804.6", "804.60", "804.61", "804.62", "804.63", "804.64", "804.65", "804.66", "804.69", "804.7", "804.70", "804.71", "804.72", "804.73", "804.74", "804.75", "804.76", "804.79", "804.8", "804.80", "804.81", "804.82", "804.83", "804.84", "804.85", "804.86", "804.89", "804.9", "804.90", "804.91", "804.92", "804.93", "804.94", "804.95", "804.96", "804.99", "805", "805.0", "805.00", "805.01", "805.02", "805.03", "805.04", "805.05", "805.06", "805.07", "805.08", "805.1", "805.10", "805.11", "805.12", "805.13", "805.14", "805.15", "805.16", "805.17", "805.18", "805.2", "805.3", "805.4", "805.5", "805.6", "805.7", "805.8", "805.9", "806", "806.0", "806.00", "806.01", "806.02", "806.03", "806.04", "806.05", "806.06", "806.07", "806.08", "806.09", "806.1", "806.10", "806.11", "806.12", "806.13", "806.14", "806.15", "806.16", "806.17", "806.18", "806.19", "806.2", "806.20", "806.21", "806.22", "806.23", "806.24", "806.25", "806.26", "806.27", "806.28", "806.29", "806.3", "806.30", "806.31", "806.32", "806.33", "806.34", "806.35", "806.36", "806.37", "806.38", "806.39", "806.4", "806.5", "806.6", "806.60", "806.61", "806.62", "806.69", "806.7", "806.70", "806.71", "806.72", "806.79", "806.8", "806.9", "807", "807.0", "807.00", "807.01", "807.02", "807.03", "807.04", "807.05", "807.06", "807.07", "807.08", "807.09", "807.1", "807.10", "807.11", "807.12", "807.13", "807.14", "807.15", "807.16", "807.17", "807.18", "807.19", "807.2", "807.3", "807.4", "807.5", "807.6", "808", "808.0", "808.1", "808.2", "808.3", "808.4", "808.41", "808.42", "808.43", "808.44", "808.49", "808.5", "808.51", "808.52", "808.53", "808.54", "808.59", "808.8", "808.9", "809", "809.0", "809.1", "810", "810.0", "810.00", "810.01", "810.02", "810.03", "810.1", "810.10", "810.11", "810.12", "810.13", "811", "811.0", "811.00", "811.01", "811.02", "811.03", "811.09", "811.1", "811.10", "811.11", "811.12", "811.13", "811.19", "812", "812.0", "812.00", "812.01", "812.02", "812.03", "812.09", "812.1", "812.10", "812.11", "812.12", "812.13", "812.19", "812.2", "812.20", "812.21", "812.3", "812.30", "812.31", "812.4", "812.40", "812.41", "812.42", "812.43", "812.44", "812.49", "812.5", "812.50", "812.51", "812.52", "812.53", "812.54", "812.59", "813", "813.0", "813.00", "813.01", "813.02", "813.03", "813.04", "813.05", "813.06", "813.07", "813.08", "813.1", "813.10", "813.11", "813.12", "813.13", "813.14", "813.15", "813.16", "813.17", "813.18", "813.2", "813.20", "813.21", "813.22", "813.23", "813.3", "813.30", "813.31", "813.32", "813.33", "813.4", "813.40", "813.41", "813.42", "813.43", "813.44", "813.45", "813.46", "813.47", "813.5", "813.50", "813.51", "813.52", "813.53", "813.54", "813.8", "813.80", "813.81", "813.82", "813.83", "813.9", "813.90", "813.91", "813.92", "813.93", "814", "814.0", "814.00", "814.01", "814.02", "814.03", "814.04", "814.05", "814.06", "814.07", "814.08", "814.09", "814.1", "814.10", "814.11", "814.12", "814.13", "814.14", "814.15", "814.16", "814.17", "814.18", "814.19", "815", "815.0", "815.00", "815.01", "815.02", "815.03", "815.04", "815.09", "815.1", "815.10", "815.11", "815.12", "815.13", "815.14", "815.19", "816", "816.0", "816.00", "816.01", "816.02", "816.03", "816.1", "816.10", "816.11", "816.12", "816.13", "817", "817.0", "817.1", "818", "818.0", "818.1", "819", "819.0", "819.1", "820", "820.0", "820.00", "820.01", "820.02", "820.03", "820.09", "820.1", "820.10", "820.11", "820.12", "820.13", "820.19", "820.2", "820.20", "820.21", "820.22", "820.3", "820.30", "820.31", "820.32", "820.8", "820.9", "821", "821.0", "821.00", "821.01", "821.1", "821.10", "821.11", "821.2", "821.20", "821.21", "821.22", "821.23", "821.29", "821.3", "821.30", "821.31", "821.32", "821.33", "821.39", "822", "822.0", "822.1", "823", "823.0", "823.00", "823.01", "823.02", "823.1", "823.10", "823.11", "823.12", "823.2", "823.20", "823.21", "823.22", "823.3", "823.30", "823.31", "823.32", "823.4", "823.40", "823.41", "823.42", "823.8", "823.80", "823.81", "823.82", "823.9", "823.90", "823.91", "823.92", "824", "824.0", "824.1", "824.2", "824.3", "824.4", "824.5", "824.6", "824.7", "824.8", "824.9", "825", "825.0", "825.1", "825.2", "825.20", "825.21", "825.22", "825.23", "825.24", "825.25", "825.29", "825.3", "825.30", "825.31", "825.32", "825.33", "825.34", "825.35", "825.39", "826", "826.0", "826.1", "827", "827.0", "827.1", "828", "828.0", "828.1", "829", "829.0", "829.1" }

**Gastroesophageal reflux disease, GERD (ICD9&10): At least 1 inpatient or outpatient code for:** : { "530.81", “K21.9”} **Procedure Code (Any Position), CPT and HCPC** is any of: { "1071F", "1118F", "4185F", "43257" }

**Peptic ulcer disease (ICD9&10): At least 1 inpatient or outpatient code for:** : { "531", "531.0", "531.00", "531.01", "531.1", "531.10", "531.11", "531.2", "531.20", "531.21", "531.3", "531.30", "531.31", "531.4", "531.40", "531.41", "531.5", "531.50", "531.51", "531.6", "531.60", "531.61", "531.7", "531.70", "531.71", "531.9", "531.90", "531.91", "532", "532.0", "532.00", "532.01", "532.1", "532.10", "532.11", "532.2", "532.20", "532.21", "532.3", "532.30", "532.31", "532.4", "532.40", "532.41", "532.5", "532.50", "532.51", "532.6", "532.60", "532.61", "532.7", "532.70", "532.71", "532.9", "532.90", "532.91", "533", "533.0", "533.00", "533.01", "533.1", "533.10", "533.11", "533.2", "533.20", "533.21", "533.3", "533.30", "533.31", "533.4", "533.40", "533.41", "533.5", "533.50", "533.51", "533.6", "533.60", "533.61", "533.7", "533.70", "533.71", "533.9", "533.90", "533.91", "534", "534.0", "534.00", "534.01", "534.1", "534.10", "534.11", "534.2", "534.20", "534.21", "534.3", "534.30", "534.31", "534.4", "534.40", "534.41", "534.5", "534.50", "534.51", "534.6", "534.60", "534.61", "534.7", "534.70", "534.71", "534.9", "534.90", "534.91", “K25.0”, “K25.1”, “K25.2”, “K25.3”, “K25.4”, “K25.5”, “K25.6”, “K25.7”, “K25.9”, “K26.0”, “K26.1”, “K26.2”, “K26.3”, “K26.4”, “K26.5”, “K26.6”, “K26.7”, “K26.9”, “K27.0”, “K27.1”, “K27.2”, “K27.3”, “K27.4”, “K27.5”, “K27.6”, “K27.7”, “K27.9”, “K28.0”, “K28.1”, “K28.2”, “K28.3”, “K28.4”, “K28.5”, “K28.6”, “K28.7”, “K28.9” }

**Psoriasis (ICD9&10): At least 1 inpatient or outpatient code for:** : { "696.1", "696.2", "696.8",  “L40.0”, “L40.1”, “L40.2”, “L40.3”, “L40.4”, “L40.8”, “L41.0”, “L41.1”, “L41.8”, “L44.8” }

**Sarcoidosis (ICD9&10): At least 1 inpatient or outpatient code for:** : { "135" , "D86.9" }

**Serious bleeding events (ICD9&10): At least 1 inpatient or outpatient code for:** : { “360.43”, “362.81”, “363.61”, “363.62”, “376.32”, “379.23”, “430”, “431”, “568.81”, “423.0”, “432.0”, “432.1”, “432.9”, “719.10”, “719.11”, “719.12”, “719.13”, “719.14”, “719.15”, “719.16”, “719.17”, “719.18”, “719.19”, “852.0”, “852.00”, “852.01”, “852.02”, “852.03”, “852.04”, “852.05”, “852.06”, “852.09”, “852.1”, “852.10”, “852.11”, “852.12”, “852.13”, “852.14”, “852.15”, “852.16”, “852.19”, “852.2”, “852.20”, “852.21”, “852.22”, “852.23”, “852.24”, “852.25”, “852.26”, “852.29”, “852.3”, “852.30”, “852.31”, “852.32”, “852.33”, “852.34”, “852.35”, “852.36”, “852.39”, “852.4”, “852.40”, “852.41”, “852.42”, “852.43”, “852.44”, “852.45”, “852.46”, “852.49”, “852.5”, “852.50”, “852.51”, “852.52”, “852.53”, “852.54”, “852.55”, “852.56”, “852.59”, “853.0”, “853.00”, “853.01”, “853.02”, “853.03”, “853.04”, “853.05”, “853.06”, “853.09”, “853.1”, “853.10”, “853.11”, “853.12”, “853.13”, “853.14”, “853.15”, “853.16”, “853.19”, “H05.239”, “H31.309”, “H31.319”, “H35.60”, “H43.13”, “H44.819”, “I60.9”, “I61.9”, “I62.00”, “I62.1”, “I62.9”, “K66.1”, “M25.00”, “M25.019”, “M25.029”, “M25.039”, “M25.049”, “M25.059”, “M25.069”, “M25.073”, “M25.076”, “M25.08”, “S06.360A”, “S06.361A”, “S06.362A”, “S06.363A”, “S06.364A”, “S06.365A”, “S06.366A”, “S06.367A”, “S06.368A”, “S06.369A”, “S06.4X0A”, “S06.4X1A”, “S06.4X2A”, “S06.4X3A”, “S06.4X4A”, “S06.4X5A”, “S06.4X6A”, “S06.4X7A”, “S06.4X8A”, “S06.4X9A”, “S06.5X0A”, “S06.5X1A”, “S06.5X2A”, “S06.5X3A”, “S06.5X4A”, “S06.5X5A”, “S06.5X6A”, “S06.5X7A”, “S06.5X8A”, “S06.5X9A”, “S06.6X0A”, “S06.6X1A”, “S06.6X2A”, “S06.6X3A”, “S06.6X4A”, “S06.6X5A”, “S06.6X6A”, “S06.6X7A”, “S06.6X8A”, “S06.6X9A” }

**Urinary tract infections (ICD9&10): At least 1 inpatient or outpatient code for**: { “590.0”, “590.01”, “590.1”, “590.10”, “595.0”, “595.1”, “595.2”, “595.3”, “595.4”, “595.8”, “595.81”, “595.82”, “595.89”, “595.9”, “599.0”, “A56.01”, “N10”, “N11.0”, “N11.1”, “N11.8”, “N30.00”, “N30.01”, “N30.20”, “N30.21”, “N30.30”, “N30.31”, “N30.80”, “N30.81”, “N30.90”, “N30.91”, “N39.0”}

**Use of H2RA: Generic Name** is any of: { "CIMETIDINE", "CIMETIDINE HCL", "CIMETIDINE HCL IN 0.9 % SODIUM CHLORIDE", "FAMOTIDINE", "FAMOTIDINE IN 0.9 % SODIUM CHLORIDE", "FAMOTIDINE IN SODIUM CHLORIDE, ISO-OSMOTIC/PF", "FAMOTIDINE/CALCIUM CARBONATE/MAGNESIUM HYDROXIDE", "FAMOTIDINE/PF", "IBUPROFEN/FAMOTIDINE", "NIZATIDINE", "RANITIDINE BISMUTH CITRATE", "RANITIDINE HCL", "RANITIDINE HCL IN 0.45 % SODIUM CHLORIDE", "RANITIDINE HCL/DIETARY SUPPLEMENT NO.17", "RANITIDINE HCL/DIETARY SUPPLEMENT NO.8" }

**Use of PPI: Generic Name** is any of: { "DEXLANSOPRAZOLE", "LANSOPRAZOLE", "OMEPRAZOLE", "PANTOPRAZOLE SODIUM", "RABEPRAZOLE SODIUM", "OMEPRAZOLE MAGNESIUM", "OMEPRAZOLE/CLARITHROMYCIN/AMOXICILLIN TRIHYDRATE", "OMEPRAZOLE/SODIUM BICARBONATE", "ASPIRIN/OMEPRAZOLE", "ESOMEPRAZOLE MAGNESIUM", "ESOMEPRAZOLE SODIUM", "ESOMEPRAZOLE STRONTIUM", "NAPROXEN/ESOMEPRAZOLE MAGNESIUM", "LANSOPRAZOLE/AMOXICILLIN TRIHYDRATE/CLARITHROMYCIN", "LANSOPRAZOLE/NAPROXEN" }

**Use of antibiotics: Generic Name** is any of: { "DAPTOMYCIN", "FIDAXOMICIN", "AMIKACIN", "AMOXICILLIN", "AZTREONAM", "BACITRACIN", "CEFACLOR", "CEFADROXIL", "CEFDINIR", "CEFIXIME", "CEFPROZIL", "CEFTAZIDIME", "CEFTIBUTEN", "CEPHALEXIN", "CEPHRADINE", "CHLORAMPHENICOL", "CIPROFLOXACIN", "CLARITHROMYCIN", "DIRITHROMYCIN", "DORIPENEM", "ENOXACIN", "GATIFLOXACIN", "LEVOFLOXACIN", "LINEZOLID", "MEROPENEM", "METRONIDAZOLE", "NALIDIXIC ACID", "NITROFURANTOIN", "NORFLOXACIN", "OFLOXACIN", "OXYTETRACYCLINE", "RIFABUTIN", "RIFAMPIN", "RIFAPENTINE", "SPARFLOXACIN", "SULFAMETHOXAZOLE", "SULFISOXAZOLE", "TELITHROMYCIN", "TETRACYCLINE", "TIGECYCLINE", "TINIDAZOLE", "TOBRAMYCIN", "AMPICILLIN ANHYDROUS", "AMPICILLIN SODIUM", "AMPICILLIN SODIUM/SULBACTAM SODIUM", "AMPICILLIN TRIHYDRATE", "BACAMPICILLIN HCL", "PENICILLIN G BENZATHINE", "PENICILLIN G BENZATHINE/PENICILLIN G PROCAINE", "PENICILLIN G POTASSIUM", "PENICILLIN G POTASSIUM IN 0.9 % SODIUM CHLORIDE", "PENICILLIN G POTASSIUM/DEXTROSE 5 % IN WATER", "PENICILLIN G POTASSIUM/DEXTROSE-WATER", "PENICILLIN G PROCAINE", "PENICILLIN G SODIUM", "PENICILLIN V POTASSIUM", "CEPHALEXIN HCL", "CEPHALOTHIN SODIUM", "CEPHALOTHIN SODIUM/DEXTROSE 5 % IN WATER", "CEPHAPIRIN SODIUM", "PENICILLAMINE", "GEMIFLOXACIN MESYLATE", "TELAVANCIN HCL", "CEFTAROLINE FOSAMIL ACETATE", "CARBENICILLIN INDANYL SODIUM", "OXACILLIN SODIUM", "OXACILLIN SODIUM IN ISO-OSMOTIC DEXTROSE", "CLOXACILLIN SODIUM", "DICLOXACILLIN SODIUM", "MEZLOCILLIN SODIUM", "NAFCILLIN IN DEXTROSE, ISO-OSMOTIC", "NAFCILLIN SODIUM", "NAFCILLIN SODIUM/DEXTROSE 5 % IN WATER", "PIPERACILLIN AND TAZOBACTAM IN DEXTROSE, ISO-OSMOTIC", "PIPERACILLIN SODIUM", "PIPERACILLIN SODIUM/DEXTROSE 5 % IN WATER", "PIPERACILLIN SODIUM/TAZOBACTAM SODIUM", "TICARCILLIN DISODIUM", "TICARCILLIN DISODIUM/DEXTROSE 5 % IN WATER", "TICARCILLIN DISODIUM/POTASSIUM CLAVULANATE", "CEFAMANDOLE NAFATE", "CEFAMANDOLE NAFATE/DEXTROSE 5 % IN WATER", "CEFAZOLIN SODIUM", "CEFAZOLIN SODIUM IN 0.9 % SODIUM CHLORIDE", "CEFAZOLIN SODIUM/DEXTROSE 5 % IN WATER", "CEFAZOLIN SODIUM/DEXTROSE, ISO-OSMOTIC", "CEFAZOLIN SODIUM/WATER FOR INJECTION,STERILE", "CEFUROXIME AXETIL", "CEFUROXIME SODIUM", "CEFUROXIME SODIUM IN 0.9 % SODIUM CHLORIDE/PF", "CEFUROXIME SODIUM/DEXTROSE 5 % IN WATER", "CEFUROXIME SODIUM/DEXTROSE, ISO-OSMOTIC", "CEFUROXIME SODIUM/WATER FOR INJECTION,STERILE", "CEFTAZIDIME IN DEXTROSE 5% AND WATER", "CEFTAZIDIME IN DEXTROSE, ISO-OSMOTIC", "CEFTAZIDIME SODIUM", "CEFTAZIDIME SODIUM IN 0.9 % SODIUM CHLORIDE", "CEFTAZIDIME SODIUM IN ISO-OSMOTIC DEXTROSE", "CEFTAZIDIME/ARGININE", "CEFTAZIDIME/AVIBACTAM SODIUM", "CEFTIZOXIME SODIUM", "CEFTIZOXIME SODIUM/DEXTROSE, ISO-OSMOTIC", "CEFTOLOZANE SULFATE/TAZOBACTAM SODIUM", "CEFTRIAXONE SODIUM", "CEFTRIAXONE SODIUM IN ISO-OSMOTIC DEXTROSE", "CEFTRIAXONE SODIUM/LIDOCAINE HCL", "CEFONICID SODIUM", "CEFOPERAZONE SODIUM", "CEFOPERAZONE SODIUM/DEXTROSE 2.4 %-WATER", "CEFOTAXIME SODIUM", "CEFOTAXIME SODIUM/DEXTROSE 5 % IN WATER", "CEFOTAXIME SODIUM/DEXTROSE, ISO-OSMOTIC", "CEFOTETAN DISODIUM", "CEFOTETAN DISODIUM IN ISO-OSMOTIC DEXTROSE", "CEFOTETAN IN DEXTROSE", "CEFOXITIN SODIUM", "CEFOXITIN SODIUM/DEXTROSE 5 % IN WATER", "CEFOXITIN SODIUM/DEXTROSE, ISO-OSMOTIC", "CEFPODOXIME PROXETIL", "CEFDITOREN PIVOXIL", "CEFEPIME HCL", "CEFEPIME HCL IN DEXTROSE 5 % IN WATER", "CEFEPIME HCL IN ISO-OSMOTIC DEXTROSE", "ERYTHROMYCIN BASE", "ERYTHROMYCIN BASE/BENZOYL PEROXIDE", "ERYTHROMYCIN BASE/ETHYL ALCOHOL", "ERYTHROMYCIN ESTOLATE", "ERYTHROMYCIN ETHYLSUCCINATE", "ERYTHROMYCIN ETHYLSUCCINATE/SULFISOXAZOLE ACETYL", "ERYTHROMYCIN GLUCEPTATE", "ERYTHROMYCIN LACTOBIONATE", "ERYTHROMYCIN STEARATE", "CLINDAMYCIN HCL", "CLINDAMYCIN PALMITATE HCL", "CLINDAMYCIN PHOSPHATE", "CLINDAMYCIN PHOSPHATE IN 0.9 % SODIUM CHLORIDE", "CLINDAMYCIN PHOSPHATE/BENZOYL PEROXIDE", "CLINDAMYCIN PHOSPHATE/BENZOYL PEROXIDE/EMOLLIENT COMB NO.94", "CLINDAMYCIN PHOSPHATE/BENZOYL PEROXIDE/HYALURONATE SODIUM", "CLINDAMYCIN PHOSPHATE/BENZOYL PEROXIDE/SKIN CLEANSER NO.5", "CLINDAMYCIN PHOSPHATE/DEXTROSE 5 % IN WATER", "CLINDAMYCIN PHOSPHATE/SKIN CLEANSER COMB NO.19", "CLINDAMYCIN PHOSPHATE/TRETINOIN", "QUINUPRISTIN/DALFOPRISTIN", "GENTAMICIN SULFATE", "GENTAMICIN SULFATE IN SODIUM CHLORIDE, ISO-OSMOTIC", "GENTAMICIN SULFATE/PF", "GENTAMICIN SULFATE/PREDNISOLONE ACETATE", "GENTAMICIN SULFATE/SODIUM CHLORIDE", "GENTAMICIN SULFATE/SODIUM CITRATE", "NEOMYCIN SULF/BACITRACIN ZINC/POLYMYXIN B SULF/PRAMOXINE HCL", "NEOMYCIN SULF/COLISTIN SUL/HYDROCORTISONE AC/THONZONIUM BROM", "NEOMYCIN SULFATE", "NEOMYCIN SULFATE/BACITRACIN ZINC/POLYMYXIN B", "NEOMYCIN SULFATE/BACITRACIN ZINC/POLYMYXIN B SULFATE", "NEOMYCIN SULFATE/BACITRACIN ZINC/POLYMYXIN B/HYDROCORTISONE", "NEOMYCIN SULFATE/BACITRACIN ZINC/POLYMYXIN B/LIDOCAINE HCL", "NEOMYCIN SULFATE/BACITRACIN/COLISTIMETHATE SODIUM", "NEOMYCIN SULFATE/BACITRACIN/POLYMYXIN B", "NEOMYCIN SULFATE/BACITRACIN/POLYMYXIN B/DIPERODON", "NEOMYCIN SULFATE/BACITRACIN/POLYMYXIN B/LIDOCAINE", "NEOMYCIN SULFATE/BACITRACIN/POLYMYXIN B/PRAMOXINE", "NEOMYCIN SULFATE/COLISTIN SULFATE/HYDROCORTISONE", "NEOMYCIN SULFATE/DEXAMETHASONE SOD PHOSPHATE", "NEOMYCIN SULFATE/FLUOCINOLONE ACETONIDE", "NEOMYCIN SULFATE/FLUOCINOLONE ACETONIDE/EMOLLIENT COMB NO.65", "NEOMYCIN SULFATE/HYDROCORTISONE", "NEOMYCIN SULFATE/HYDROCORTISONE ACETATE", "NEOMYCIN SULFATE/POLYMYXIN B SULFATE", "NEOMYCIN SULFATE/POLYMYXIN B SULFATE/BUFFERS/HYDROCORTISONE", "NEOMYCIN SULFATE/POLYMYXIN B SULFATE/GRAMICIDIN D", "NEOMYCIN SULFATE/POLYMYXIN B SULFATE/HYDROCORTISONE", "NEOMYCIN SULFATE/POLYMYXIN B SULFATE/LIDOCAINE", "NEOMYCIN SULFATE/POLYMYXIN B SULFATE/PRAMOXINE", "NEOMYCIN SULFATE/POLYMYXIN B SULFATE/PREDNISOLONE", "NEOMYCIN/BACITRACIN/POLYMYXIN B/HYDROCORTISONE", "NEOMYCIN/POLYMYXIN B SULFATE/DEXAMETHASONE", "STREPTOMYCIN SULFATE", "VANCOMYCIN HCL", "VANCOMYCIN HCL/BALANCED SALT SOLUTION NO.2/PF", "VANCOMYCIN IN 0.9 % SODIUM CHLORIDE", "VANCOMYCIN IN 5 % DEXTROSE IN WATER", "CYCLOSERINE", "POLYMYXIN B SULFATE", "POLYMYXIN B SULFATE,MICRONIZED", "POLYMYXIN B SULFATE/HYDROCORTISONE", "POLYMYXIN B SULFATE/TRIMETHOPRIM", "MOXIFLOXACIN HCL", "MOXIFLOXACIN HCL IN BALANCE SALT IRRIGATION SOLUTION NO.2/PF", "MOXIFLOXACIN HCL IN SODIUM ACETATE AND SULFATE,WATER,ISO-OSM", "MOXIFLOXACIN HCL/SODIUM CHLORIDE, ISO-OSMOTIC", "ALATROFLOXACIN MESYLATE", "BESIFLOXACIN HCL", "CIPROFLOXACIN HCL", "CIPROFLOXACIN HCL/DEXAMETHASONE", "CIPROFLOXACIN HCL/FLUOCINOLONE ACETONIDE", "CIPROFLOXACIN HCL/HYDROCORTISONE", "CIPROFLOXACIN LACTATE", "CIPROFLOXACIN LACTATE/DEXTROSE 5 % IN WATER", "CIPROFLOXACIN/CIPROFLOXACIN HCL", "DELAFLOXACIN MEGLUMINE", "GATIFLOXACIN/DEXTROSE 5 % IN WATER", "GATIFLOXACIN/PREDNISOLONE ACETATE", "GATIFLOXACIN/PREDNISOLONE ACETATE/NEPAFENAC", "GREPAFLOXACIN HCL", "LEVOFLOXACIN/DEXTROSE 5 % IN WATER", "LOMEFLOXACIN HCL", "OFLOXACIN/DEXTROSE 5 % IN WATER", "TROVAFLOXACIN MESYLATE", "OXYTETRACYCLINE HCL/SULFAMETHIZOLE/PHENAZOPYRIDINE", "TRIMETHOPRIM", "TRIMETHOPRIM, MICRONIZED", "SULFAMETHOXAZOLE/TRIMETHOPRIM", "DOXYCYCLINE CALCIUM", "DOXYCYCLINE HYCLATE", "DOXYCYCLINE HYCLATE/EYELID CLEANSER 3/EYELID EMOLLIENT NO.1", "DOXYCYCLINE HYCLATE/EYELID CLEANSER NO2/EYELID CLEANSER NO3", "DOXYCYCLINE HYCLATE/SKIN CLEANSER COMBINATION NO.19", "DOXYCYCLINE MONOHYDRATE", "DOXYCYCLINE MONOHYDRATE/BENZOYL PEROXIDE", "DOXYCYCLINE MONOHYDRATE/OMEGA-3 COMBINATION NO.1/EYE MASK", "DOXYCYCLINE MONOHYDRATE/SALICYLIC ACID/OCTINOXATE/ZINC OXIDE", "DOXYCYCLINE MONOHYDRATE/SKIN CLEANSER COMBINATION NO.9", "MINOCYCLINE HCL", "MINOCYCLINE HCL MICROSPHERES", "MINOCYCLINE HCL/EMOL COMB NO.16/SKIN CLNSR L4/TOP AGENT NO.3", "MINOCYCLINE HCL/EYELID CLEANSER COMBINATION NO. 1", "MINOCYCLINE HCL/WIPES WITH SKIN CLEANSER NO.4", "DEMECLOCYCLINE HCL", "AMIKACIN SULFATE", "AMIKACIN SULFATE IN 0.9 % SODIUM CHLORIDE", "AMIKACIN SULFATE/PF", "AMOXICILLIN/POTASSIUM CLAVULANATE", "LANSOPRAZOLE/AMOXICILLIN TRIHYDRATE/CLARITHROMYCIN", "OMEPRAZOLE/CLARITHROMYCIN/AMOXICILLIN TRIHYDRATE", "ERTAPENEM SODIUM", "AZTREONAM LYSINE", "AZTREONAM/DEXTROSE-WATER", "IMIPENEM/CILASTATIN SODIUM", "MEROPENEM IN 0.9 % SODIUM CHLORIDE", "MEROPENEM/VABORBACTAM", "LINCOMYCIN HCL", "KANAMYCIN SULFATE", "NETILMICIN SULFATE", "PAROMOMYCIN SULFATE", "TOBRAMYCIN IN 0.225 % SODIUM CHLORIDE", "TOBRAMYCIN SULFATE", "TOBRAMYCIN SULFATE/DEXTROSE 5 % IN WATER", "TOBRAMYCIN SULFATE/SODIUM CHLORIDE", "TOBRAMYCIN/DEXAMETHASONE", "TOBRAMYCIN/LOTEPREDNOL ETABONATE", "TOBRAMYCIN/NEBULIZER", "LINEZOLID IN 0.9 % SODIUM CHLORIDE", "RIFAMPIN/ISONIAZID", "RIFAMPIN/ISONIAZID/PYRAZINAMIDE", "CAPREOMYCIN SULFATE", "SULFAMETHOXAZOLE/PHENAZOPYRIDINE HCL", "SULFISOXAZOLE ACETYL", "SULFISOXAZOLE/PHENAZOPYRIDINE HCL", "OXYTETRACYCLINE HCL", "OXYTETRACYCLINE HCL/HYDROCORTISONE ACETATE", "OXYTETRACYCLINE HCL/POLYMYXIN B SULFATE", "OXYTETRACYCLINE/LIDOCAINE", "TETRACYCLINE HCL", "BISMUTH SUBSALICYLATE/METRONIDAZOLE/TETRACYCLINE HCL", "CHLORTETRACYCLINE HCL", "COLLOIDAL BISMUTH SUBCITRATE/METRONIDAZOLE/TETRACYCLINE HCL", "CHLORAMPHENICOL PALMITATE", "CHLORAMPHENICOL SOD SUCCINATE", "CHLORAMPHENICOL/FIBRINOLYSIN/DESOXYRIBONUCLEASE", "METRONIDAZOLE BENZOATE", "METRONIDAZOLE HCL", "METRONIDAZOLE IN SODIUM CHLORIDE", "METRONIDAZOLE/SKIN CLEANSER", "METRONIDAZOLE/SKIN CLEANSER COMBINATION NO.23", "NITROFURANTOIN MACROCRYSTAL", "NITROFURANTOIN MONOHYDRATE/MACROCRYSTALS" }

**Use of antiplatelets**: **Generic Name** is any of: { "ASPIRIN", "ASPIRIN (CALCIUM CARB & MAGNESIUM BUFFERS)/PRAVASTATIN", "ASPIRIN/ACETAMINOPHEN", "ASPIRIN/ACETAMINOPHEN/CAFFEINE", "ASPIRIN/ACETAMINOPHEN/CAFFEINE/CALCIUM", "ASPIRIN/ACETAMINOPHEN/CAFFEINE/POTASSIUM", "ASPIRIN/ACETAMINOPHEN/CALCIUM CARBONATE", "ASPIRIN/ACETAMINOPHEN/MAGNESIUM/ALUMINUM HYDROXIDE/CAFFEINE", "ASPIRIN/CAFFEINE", "ASPIRIN/CALCIUM CARBONATE", "ASPIRIN/CALCIUM CARBONATE/MAGNESIUM", "ASPIRIN/CALCIUM CARBONATE/MAGNESIUM/ALUMINUM HYDROXIDE", "ASPIRIN/CODEINE PHOSPHATE", "ASPIRIN/DIPHENHYDRAMINE CITRATE", "ASPIRIN/DIPHENHYDRAMINE HCL", "ASPIRIN/DIPHENHYDRAMINE/SODIUM BICARBONATE/CITRIC ACID", "ASPIRIN/DIPYRIDAMOLE", "ASPIRIN/MAGNESIUM CARBONATE/DIHYDROXYALUMINUM AMINOACETATE", "ASPIRIN/MAGNESIUM HYDROXIDE/ALUMINUM HYDROXIDE", "ASPIRIN/MAGNESIUM HYDROXIDE/ALUMINUM HYDROXIDE/CAFFEINE", "ASPIRIN/MEPROBAMATE", "ASPIRIN/SALICYLAMIDE/ACETAMINOPHEN/CAFFEINE", "ASPIRIN/SALICYLAMIDE/CAFFEINE", "ASPIRIN/SODIUM BICARBONATE/CITRIC ACID", "CILOSTAZOL", "CLOPIDOGREL BISULFATE", "DIPYRIDAMOLE", "PRASUGREL HCL", "TICAGRELOR", "VORAPAXAR SULFATE", "ABCIXIMAB", "BUTALBITAL/ASPIRIN/CAFFEINE", "CARISOPRODOL/ASPIRIN", "CHLORPHENIRAMINE MAL/PHENYLEPHRINE/D-METHORPHAN HB/ASPIRIN", "CHLORPHENIRAMINE MALEATE/PHENYLEPHRINE BITARTRATE/ASPIRIN", "CINNAMEDRINE HCL/ASPIRIN/CAFFEINE", "CODEINE PHOSPHATE/BUTALBITAL/ASPIRIN/CAFFEINE", "CODEINE PHOSPHATE/CARISOPRODOL/ASPIRIN", "CODEINE/ASPIRIN/SALICYLAMIDE/ACETAMINOPHEN/CAFFEINE", "DIHYDROCODEINE BITARTRATE/ASPIRIN/CAFFEINE", "DIHYDROCODEINE/ASPIRIN/CAFFEINE", "EPHEDRINE/ASPIRIN/ACETANILIDE/CAFFEINE", "HYDROCODONE BITARTRATE/ASPIRIN", "METHOCARBAMOL/ASPIRIN", "ORPHENADRINE CITRATE/ASPIRIN/CAFFEINE", "OXYCODONE HCL/ASPIRIN", "OXYCODONE HCL/OXYCODONE TEREPHTHALATE/ASPIRIN", "OXYCODONE/ASPIRIN", "PENTAZOCINE HCL/ASPIRIN", "PHENYLEPHRINE HCL/ASPIRIN", "PHENYLPROPANOLAMINE BITARTRATE/ASPIRIN", "PHENYLPROPANOLAMINE BITARTRATE/ASPIRIN/CHLORPHENIRAMINE", "PHENYLPROPANOLAMINE HCL/ASPIRIN", "PHENYLPROPANOLAMINE HCL/ASPIRIN/CHLORPHENIRAMINE", "PHENYLPROPANOLAMINE HCL/ASPIRIN/CHLORPHENIRAMINE/CAFFEINE", "PHENYLPROPANOLAMINE HCL/ASPIRIN/DIPHENHYDRAMINE", "PROPOXYPHENE HCL/ASPIRIN/CAFFEINE", "PSEUDOEPHEDRINE HCL/ASPIRIN/CHLORPHENIRAMINE" }

**Use of non steroidal anti inflammatory drug, Generic Name** is any of: { "CELECOXIB", "CHLORPHENIRAMINE MALEATE/PHENYLEPHRINE HCL/IBUPROFEN", "CHLORPHENIRAMINE MALEATE/PSEUDOEPHEDRINE HCL/IBUPROFEN", "DICLOFENAC EPOLAMINE", "DICLOFENAC POTASSIUM", "DICLOFENAC SODIUM", "DICLOFENAC SODIUM/MISOPROSTOL", "DICLOFENAC SUBMICRONIZED", "ETODOLAC", "FENOPROFEN CALCIUM", "FLURBIPROFEN", "FLURBIPROFEN SODIUM", "GOLD SODIUM THIOMALATE", "HYDROCODONE/IBUPROFEN", "IBUPROFEN", "IBUPROFEN LYSINE/PF", "IBUPROFEN/CAFFEINE/VITAMINS B1, B2, B6, & B12", "IBUPROFEN/DIETARY SUPPLEMENT,MISC. CB.11", "IBUPROFEN/DIPHENHYDRAMINE CITRATE", "IBUPROFEN/DIPHENHYDRAMINE HCL", "IBUPROFEN/FAMOTIDINE", "IBUPROFEN/IRRITANTS COUNTER-IRRITANTS COMBINATION #2", "IBUPROFEN/OXYCODONE HCL", "IBUPROFEN/PHENYLEPHRINE HCL", "IBUPROFEN/PSEUDOEPHEDRINE HCL", "INDOMETHACIN", "INDOMETHACIN SODIUM", "KETOPROFEN", "KETOPROFEN, MICRONIZED", "KETOROLAC TROMETHAMINE", "KETOROLAC TROMETHAMINE/PF", "LANSOPRAZOLE/NAPROXEN", "MECLOFENAMATE SODIUM", "MEFENAMIC ACID", "MELOXICAM", "MELOXICAM/IRRITANTS COUNTER-IRRITANTS COMBINATION NO.2", "NABUMETONE", "NABUMETONE, MICRONIZED", "NAPROXEN", "NAPROXEN SODIUM", "NAPROXEN SODIUM/PSEUDOEPHEDRINE HCL", "NAPROXEN/DIETARY SUPPLEMENT,MISC. CB.11", "NAPROXEN/ESOMEPRAZOLE MAGNESIUM", "NAPROXEN/IRRITANTS COUNTER-IRRITANTS COMBINATION #2", "OXAPROZIN", "PIROXICAM", "PIROXICAM/DIETARY SUPPLEMENT,MISC. CB.11", "SULINDAC", "SUMATRIPTAN SUCCINATE/NAPROXEN SODIUM" } **Procedure Code (Any Position), CPT and HCPC** is any of: { "C9279", "C9447", "J1600", "J1741", "J1885" }

**Trends Over Calendar Time**

Trends in population characteristics and values were evaluated over calendar time. Values and baseline characteristics were assessed over 1-year intervals beginning on Jan 01, 2007 and ending at the Dec 31, 2016.

**Analysis plan**

The study outcome will be measured as proportion per 100 patients. Follow-up will begin on the cohort entry date and continue until first of: occurrence of outcome event, disenrollment from the insurance or drug coverage plan, death, hospitalization or nursing home admission (IBM MarketScan only has outpatient dispending records that do not capture medication use in the hospital or skilled nursing facility), or 7 days after cohort entry date. The association between patient characteristics and the outcome will be assessed by univariate and multivariate logistic regression. We will use multivariate logistic regression to adjust for the following covariates: age, sex, ARTI indications, geographical region, provider type (nurse practitioner, physician assistants, general medicine physicians, medical specialists, ENT doctors, vs. emergency department [ED] physicians), care location (regular office, urgent care, walk-in retail clinic, vs. ED), employment status, insurance plan, related prescription drug use (nonsteroidal anti-inflammatory drugs, proton-pump inhibitors, histamine-2-receptor antagonists, antibiotics, antiplatelets, anticoagulants), and multiple co-morbidities, including diabetes mellites, hypertension, stroke, kidney dysfunction, dementia, obesity, heart failure, ischemic heart disease, atrial fibrillation, venous thromboembolism, urinary tract infections, human immunodeficiency virus infection/acquired immune deficiency syndrome (HIV/AIDS), fractures, prior falls, gastroesophageal reflux disease (GERD), peptic ulcer disease, major bleeding events, bronchiectasis, connective tissue diseases, and remote history of non-septic arthritis or spondyloarthropathy, the combined co-morbidity score proposed by Gagne et al and calendar year. The following secondary analyses will be conducted to test robustness of our findings: 1) Restricting to use of systemic steroids within 3 days of cohort entry. 2) Excluded patients with nasal polyps because systemic steroids are commonly prescribed to treat sinusitis by ENT physician for sinusitis with polyposis. All analyses will be conducted using the Aetion platform and R, version 3.1.2.5 (R Foundation for Statistical Computing).

**References**

1. Stergachis AS. Record linkage studies for postmarketing drug surveillance: Data quality and validity considerations. *Drug Intell Clin Pharm*. 1988;22(2):157–161. Available at: <http://www.ncbi.nlm.nih.gov/pubmed/3349931>.

2. Levy AR, O’Brien BJ, Sellors C, Grootendorst P, Willison D. Coding accuracy of administrative drug claims in the ontario drug benefit database. *Can J Clin Pharmacol*. 2003;10(2):67–71. Available at: <http://www.ncbi.nlm.nih.gov/pubmed/12879144>.

3. West SL, Savitz DA, Koch G, Strom BL, Guess HA, Hartzema A. Recall accuracy for prescription medications: Self-report compared with database information. *Am J Epidemiol*. 1995;142(10):1103–1112. Available at: <http://www.ncbi.nlm.nih.gov/pubmed/7485055>.

4. West SL, Strom BL, Freundlich B, Normand E, Koch G, Savitz DA. Completeness of prescription recording in outpatient medical records from a health maintenance organization. *J Clin Epidemiol*. 1994;47(2):165–171. Available at: <http://www.ncbi.nlm.nih.gov/pubmed/8113825>.

5. Fischer MA, Stedman MR, Lii J, et al. Primary medication non-adherence: Analysis of 195,930 electronic prescriptions. *J Gen Intern Med*. 2010;25(4):284–290. Available at: <http://www.ncbi.nlm.nih.gov/pubmed/20131023>.
