# Supplementary material for: Prescribing systemic steroids for acute respiratory tract infections in United States outpatient settings: A nationwide population-based cohort study
Source: PLoS Med. 2020 Mar 31;17(3):e1003058. doi: 10.1371/journal.pmed.1003058 (PMC7108689; doi:10.1371/journal.pmed.1003058)
Supplement: S1 Table — (DOCX) [file pmed.1003058.s003.docx]

**S1 Table. Patient characteristics and association with use of systemic corticosteroids within 3 days of an outpatient diagnosis of acute respiratory tract infections**

| Characteristic | Total population, N= 9,763,710, N | Receiving steroids*, N= 1,092,626, N (%) | Univariate OR | Multivariate adjusted OR** |
| --- | --- | --- | --- | --- |
| Age categories |  |  |  |  |
| 18 to <25 | 1,752,290 | 173,692 (15.9%) | Ref | Ref |
| 25 to <35 | 1,975,722 | 211,435 (19.4%) | 1.09 (1.08, 1.10) | 1.08 (1.07, 1.09) |
| 35 to <45 | 2,231,358 | 253,798 (23.2%) | 1.17 (1.16, 1.17) | 1.18 (1.17, 1.19) |
| 45 to <55 | 2,167,879 | 261,055 (23.9%) | 1.24 (1.24, 1.25) | 1.26 (1.25, 1.27) |
| >= 55 | 1,636,461 | 192,646 (17.6%) | 1.21 (1.20, 1.22) | 1.22 (1.21, 1.23) |
| Sex |  |  |  |  |
| Male | 4,297,795 | 514,466 (47.1%) | Ref | Ref |
| Female | 5,465,915 | 578,160 (52.9%) | 0.87 (0.87, 0.87) | 0.86 (0.86, 0.87) |
| ARTI indication |  |  |  |  |
| Unspecified upper respiratory infections | 2,354,975 | 210,746 (19.3%) | 0.73 (0.72, 0.73) | 1.25 (1.24, 1.26) |
| Otitis media | 248,978 | 27,774 (2.5%) | 1.00 (0.98, 1.01) | 1.40 (1.38, 1.42) |
| Sinusitis | 2,398,502 | 346,460 (31.7%) | 1.50 (1.49, 1.50) | 2.10 (2.09, 2.12) |
| Acute pharyngitis | 2,272,142 | 227,550 (20.8%) | 0.85 (0.85, 0.86) | 1.42 (1.41, 1.43) |
| Allergic rhinitis | 1,498,960 | 190,802 (17.5%) | 1.19 (1.18, 1.20) | 1.81 (1.80, 1.83) |
| Acute bronchitis | 1,442,379 | 248,219 (22.7%) | 1.84 (1.83, 1.85) | 2.75 (2.74, 2.77) |
| Pneumonia | 235,440 | 25,344 (2.3%) | 0.96 (0.94, 0.97) | 1.73 (1.71, 1.76) |
| Influenza | 332,623 | 18,284 (1.7%) | 0.45 (0.45, 0.46) | 0.62 (0.61, 0.63) |
| Region |  |  |  |  |
| Northeast | 1,519,033 | 81,200 (7.4%) | Ref | Ref |
| North Central | 2,231,576 | 163,926 (15.0%) | 1.40 (1.39, 1.42) | 1.45 (1.44, 1.46) |
| South | 4,120,036 | 738,970 (67.6%) | 3.87 (3.84, 3.90) | 4.03 (4.00, 4.06) |
| West | 1,740,782 | 95,158 (8.7%) | 1.02 (1.01, 1.03) | 1.11 (1.10, 1.12) |
| Unknown | 152,283 | 13,372 (1.2%) | 1.70 (1.67, 1.74) | 1.74 (1.71, 1.77) |
| Provider type |  |  |  |  |
| General medicine | 8,232,102 | 895,606 (82.0%) | Ref | Ref |
| Medical specialist | 214,225 | 15,826 (1.4%) | 0.65 (0.64, 0.66) | 0.67 (0.65, 0.68) |
| ED physician | 559,897 | 76,688 (7.0%) | 1.30 (1.29, 1.31) | 1.16 (1.15, 1.17) |
| ENT physician | 218,938 | 35,654 (3.3%) | 1.59 (1.58, 1.61) | 1.48 (1.47, 1.50) |
| Nurse practitioner | 372,791 | 50,598 (4.6%) | 1.29 (1.27, 1.30) | 1.09 (1.08, 1.10) |
| Physician assistant | 164,030 | 18,169 (1.7%) | 1.02 (1.00, 1.04) | 1.10 (1.08, 1.11) |
| Care location |  |  |  |  |
| Regular office visit | 9,034,253 | 994,381 (91.0%) | Ref | Ref |
| Urgent care | 328,244 | 45,609 (4.2%) | 1.30 (1.29, 1.32) | 1.26 (1.25, 1.28) |
| Walk-in retail clinic | 7,467 | 636 (0.1%) | 0.75 (0.69, 0.82) | 0.62 (0.57, 0.68) |
| Emergency room | 393,746 | 52,000 (4.8%) | 1.23 (1.22, 1.24) | 1.15 (1.14, 1.17) |
| DM | 560,839 | 51,134 (4.7%) | 0.79 (0.78, 0.79) | 0.68 (0.67, 0.68) |
| HTN | 1,440,025 | 178,361 (16.3%) | 1.15 (1.14, 1.15) | 1.13 (1.12, 1.14) |
| Stroke | 48,340 | 5,298 (0.5%) | 0.98 (0.95, 1.01) | 0.97 (0.94, 1.00) |
| Characteristic |  |  |  |  |
| Kidney dysfunction | 40,425 | 3,788 (0.3%) | 0.82 (0.79, 0.85) | 0.92 (0.89, 0.96) |
| Liver disease | 129,804 | 13,345 (1.2%) | 0.91 (0.89, 0.92) | 0.96 (0.94, 0.97) |
| Dementia | 11,307 | 1,085 (0.1%) | 0.84 (0.79, 0.90) | 0.86 (0.80, 0.91) |
| Obesity | 312,085 | 36,872 (3.4%) | 1.07 (1.05, 1.08) | 0.99 (0.98, 1.00) |
| Heart failure | 30,396 | 3,056 (0.3%) | 0.89 (0.85, 0.92) | 1.00 (0.96, 1.05) |
| Ischemic heart disease | 179,551 | 21,813 (2.0%) | 1.10 (1.08, 1.12) | 1.02 (1.00, 1.04) |
| Atrial fibrillation | 46,100 | 4,645 (0.4%) | 0.89 (0.86, 0.92) | 0.98 (0.94, 1.01) |
| VTE | 16,022 | 1,489 (0.1%) | 0.81 (0.77, 0.86) | 0.94 (0.88, 0.99) |
| Urinary tract infections | 571,447 | 59,998 (5.5%) | 0.93 (0.92, 0.94) | 0.94 (0.93, 0.95) |
| HIV/AIDS | 16,785 | 1,268 (0.1%) | 0.65 (0.61, 0.69) | 0.62 (0.59, 0.66) |
| Fractures | 118,412 | 12,962 (1.2%) | 0.98 (0.96, 0.99) | 1.02 (1.00, 1.04) |
| Falls | 29,154 | 3,422 (0.3%) | 1.06 (1.02, 1.09) | 1.02 (0.99, 1.06) |
| GERD | 353,109 | 41,903 (3.8%) | 1.07 (1.06, 1.08) | 0.93 (0.92, 0.94) |
| Peptic ulcer disease | 19,689 | 2,308 (0.2%) | 1.05 (1.01, 1.10) | 1.00 (0.95, 1.05) |
| Major bleeding events | 12,886 | 1,142 (0.1%) | 0.77 (0.73, 0.82) | 0.84 (0.79, 0.90) |
| Bronchiectasis | 1,382 | 99 (0.0%) | 0.61 (0.50, 0.75) | 0.69 (0.56, 0.85) |
| Connective tissue diseases | 6,245 | 703 (0.1%) | 1.01 (0.93, 1.09) | 0.93 (0.86, 1.01) |
| Use of NSAIDs | 1,177,851 | 142,425 (13.0%) | 1.11 (1.10, 1.11) | 1.08 (1.08, 1.09) |
| Use of PPI | 672,651 | 84,357 (7.7%) | 1.15 (1.14, 1.16) | 1.13 (1.12, 1.14) |
| Use of H2RA | 94,435 | 9,776 (0.9%) | 0.92 (0.90, 0.94) | 0.98 (0.95, 1.00) |
| Use of antibiotics | 2,930,232 | 339,311 (31.1%) | 1.06 (1.05, 1.06) | 1.09 (1.08, 1.09) |
| Use of antiplatelets | 90,369 | 11,438 (1.0%) | 1.15 (1.13, 1.17) | 1.07 (1.05, 1.10) |
| Use of anticoagulants | 52,308 | 4,991 (0.5%) | 0.84 (0.81, 0.86) | 0.88 (0.85, 0.91) |
| Combined co-morbidity score category |  |  |  |  |
| < 1 | 7,625,943 | 849,145 (77.7%) | Ref | Ref |
| 1 - 2 | 1,707,347 | 198,050 (18.1%) | 1.05 (1.04, 1.05) | 0.90 (0.89, 0.91) |
| 2 - 4 | 380,088 | 40,930 (3.7%) | 0.96 (0.95, 0.97) | 0.84 (0.83, 0.85) |
| >= 4 | 50,332 | 4,501 (0.4%) | 0.78 (0.76, 0.81) | 0.70 (0.67, 0.73) |
| Employment status |  |  |  |  |
| Active full time | 5,508,470 | 616,119 (56.4% | Ref | Ref |
| retiree | 503,273 | 55,027 (5.0%) | 0.97 (0.97, 0.98) | 0.98 (0.97, 1.00) |
| active part-time | 104,025 | 9,636 (0.9%) | 0.81 (0.79, 0.83) | 0.90 (0.89, 0.92) |
| Unknown/other | 3,647,942 | 411,844 (37.7%) | 1.01 (1.01, 1.01) | 1.00 (0.99, 1.00) |
| Insurance plan type |  |  |  |  |
| PPO | 5,960,060 | 709,710 (65.0%) | Ref | Ref |
| Comprehensive | 179,535 | 19,031 (1.7%) | 0.88 (0.86, 0.89) | 1.07 (1.05, 1.09) |
| EPO | 129,760 | 10,706 (1.0%) | 0.67 (0.65, 0.68) | 0.84 (0.83, 0.86) |
| HMO | 1,431,532 | 108,275 (9.9%) | 0.61 (0.60, 0.61) | 0.71 (0.71, 0.72) |
| POS | 803,251 | 105,622 (9.7%) | 1.12 (1.11, 1.13) | 1.15 (1.14, 1.16) |
| CDHP | 550,856 | 69,286 (6.3%) | 1.06 (1.06, 1.07) | 0.93 (0.93, 0.94) |
| HDHP | 341,572 | 35,013 (3.2%) | 0.84 (0.84, 0.85) | 0.81 (0.81, 0.82) |
| Others/missing | 367,144 | 34,983 (3.2%) | 0.78 (0.77, 0.79) | 1.00 (0.99, 1.01) |
| Year of Cohort Entry Date | | |  |  |
| 2007 | 1,368,068 | 135,472 (12.4%) | Ref | Ref |
| 2008 | 1,194,950 | 118,976 (10.9%) | 1.01 (1.00, 1.01) | 1.10 (1.09, 1.11) |
| 2009 | 1,292,822 | 117,366 (10.7%) | 0.91 (0.90, 0.92) | 1.04 (1.03, 1.05) |
| 2010 | 991,226 | 104,920 (9.6%) | 1.08 (1.07, 1.09) | 1.25 (1.24, 1.26) |
| 2011 | 1,040,685 | 104,446 (9.6%) | 1.01 (1.01, 1.02) | 1.21 (1.20, 1.22) |
| 2012 | 1,104,266 | 137,346 (12.6%) | 1.29 (1.28, 1.30) | 1.45 (1.44, 1.47) |
| 2013 | 837,644 | 98,226 (9.0%) | 1.21 (1.20, 1.22) | 1.50 (1.48, 1.51) |
| 2014 | 758,101 | 95,574 (8.7%) | 1.31 (1.30, 1.32) | 1.59 (1.57, 1.60) |
| 2015 | 576,617 | 85,494 (7.8%) | 1.58 (1.57, 1.60) | 1.78 (1.77, 1.80) |
| 2016 | 599,331 | 94,806 | 1.71 (1.69, 1.73) | 1.96 (1.94, 1.98) |
| *Within 3 days of an ARTI **Adjusted for all the variables listed in Table 2. ARTI= acute respiratory tract infections, ENT= otolaryngology, DM= diabetes mellites, VTE= venous thromboembolism, HIV/AIDS=acquired immune deficiency syndrome, GERD=gastroesophageal reflux disease, NSAIDs=nonsteroidal anti-inflammatory drugs, PPIs=proton-pump inhibitors, H2RA=histamine 2 receptor antagonists, COBRA=Consolidated Omnibus Budget Reconciliation Act, PPO=preferred provider organization , HMO=health maintenance organization, EPO= exclusive provider organization, POS=point of service, CDHP= consumer-driven health plan, HDHP= high-deductible health plan | | | | |
