# Supplementary material for: Prescribing systemic steroids for acute respiratory tract infections in United States outpatient settings: A nationwide population-based cohort study
Source: PLoS Med. 2020 Mar 31;17(3):e1003058. doi: 10.1371/journal.pmed.1003058 (PMC7108689; doi:10.1371/journal.pmed.1003058)
Supplement: S2 Table — (DOCX) [file pmed.1003058.s004.docx]

**S2 Table. Associations between geographical region and use of systemic steroids within 3 days of an acute respiratory tract infection diagnosis**

| **Variable** | **Adjusted OR (95% CI)** |
| --- | --- |
| **Any steroids** |  |
| North Central vs. Northeast | 1.45 (1.44, 1.46) |
| South vs. Northeast | 4.03 (4.00, 4.06) |
| West vs. Northeast | 1.11 (1.10, 1.12) |
| Unknown vs. Northeast | 1.74 (1.71, 1.77) |
| **Parenteral steroids (IV or IM)** |  |
| North Central vs. Northeast | 2.73 (2.67, 2.78) |
| South vs. Northeast | 15.54 (15.27, 15.82) |
| West vs. Northeast | 2.50 (2.44, 2.55) |
| Unknown vs. Northeast | 3.94 (3.82, 4.08) |
| **Oral steroids** |  |
| North Central vs. Northeast | 1.24 (1.23, 1.25) |
| South vs. Northeast | 1.73 (1.71, 1.75) |
| West vs. Northeast | 0.85 (0.84, 0.86) |
| Unknown vs. Northeast | 1.37 (1.34, 1.41) |
| Adjusted for all the variables listed in Table2, OR=odds ratio, CI=confidence interval | |
