# Supplementary material for: Prescribing systemic steroids for acute respiratory tract infections in United States outpatient settings: A nationwide population-based cohort study
Source: PLoS Med. 2020 Mar 31;17(3):e1003058. doi: 10.1371/journal.pmed.1003058 (PMC7108689; doi:10.1371/journal.pmed.1003058)
Supplement: S3 Table — (DOCX) [file pmed.1003058.s005.docx]

**S3 Table. Patient characteristics by geographical region**

| **Characteristic** | **South, N (%)** | **Northeast, N (%)** | **North Central, N (%)** | **West, N (%)** | **Unknown, N (%)** | **Adjusted OR* (95% CI)** |
| --- | --- | --- | --- | --- | --- | --- |
| Number of patients | 4,120,036 | 1,519,033 | 2,231,576 | 1,740,782 | 152,283 |  |
| Age, mean (SD) | 39.51 (13.22) | 39.77 (13.40) | 39.54 (13.62) | 39.56 (13.34) | 39.15 (13.26) |  |
| Age categories |  |  |  |  |  |  |
| 18 to <25 | 711,655 (17.3%) | 276,020 (18.2%) | 422,238 (18.9%) | 314,496 (18.1%) | 27,881 (18.3%) | Ref |
| 25 to <35 | 864,980 (21.0%) | 290,847 (19.1%) | 440,409 (19.7%) | 347,271 (19.9%) | 32,215 (21.2%) | 1.08 (1.07, 1.08) |
| 35 to <45 | 958,759 (23.3%) | 348,644 (23.0%) | 488,151 (21.9%) | 400,977 (23.0%) | 34,827 (22.9%) | 1.01 (1.00, 1.01) |
| 45 to <55 | 912,465 (22.1%) | 344,098 (22.7%) | 489,506 (21.9%) | 388,339 (22.3%) | 33,471 (22.0%) | 0.93 (0.93, 0.94) |
| >= 55 | 672,177 (16.3%) | 259,424 (17.1%) | 391,272 (17.5%) | 289,699 (16.6%) | 23,889 (15.7%) | 0.89 (0.88, 0.89) |
| Sex |  |  |  |  |  |  |
| Male | 1,794,785 (43.6%) | 680,428 (44.8%) | 988,807 (44.3%) | 767,422 (44.1%) | 66,353 (43.6%) | Ref |
| Female | 2,325,251 (56.4%) | 838,605 (55.2%) | 1,242,769 (55.7%) | 973,360 (55.9%) | 85,930 (56.4%) | 1.03 (1.03, 1.04) |
| ARTI indications |  |  |  |  |  |  |
| acute upper respiratory infections | 1,010,439 (24.5%) | 384,043 (25.3%) | 482,181 (21.6%) | 441,386 (25.4%) | 37,415 (24.6%) | 1.48 (1.48, 1.49) |
| otitis media | 103,540 (2.5%) | 41,025 (2.7%) | 59,507 (2.7%) | 41,236 (2.4%) | 3,698 (2.4%) | 1.29 (1.28, 1.30) |
| sinusitis | 1,065,358 (25.9%) | 344,219 (22.7%) | 592,033 (26.5%) | 358,706 (20.6%) | 38,436 (25.2%) | 1.54 (1.54, 1.55) |
| acute pharyngitis | 900,305 (21.9%) | 372,922 (24.5%) | 552,062 (24.7%) | 412,583 (23.7%) | 34,699 (22.8%) | 1.24 (1.24, 1.25) |
| allergic rhinitis | 703,411 (17.1%) | 206,093 (13.6%) | 269,430 (12.1%) | 299,666 (17.2%) | 20,558 (13.5%) | 1.77 (1.76, 1.78) |
| acute bronchitis | 605,147 (14.7%) | 224,051 (14.7%) | 349,097 (15.6%) | 239,463 (13.8%) | 24,943 (16.4%) | 1.38 (1.37, 1.38) |
| pneumonia | 80,517 (2.0%) | 39,946 (2.6%) | 60,321 (2.7%) | 51,136 (2.9%) | 3,596 (2.4%) | 1.01 (1.00, 1.02) |
| influenza | 170,368 (4.1%) | 39,235 (2.6%) | 64,498 (2.9%) | 51,508 (3.0%) | 7,074 (4.6%) | 2.11 (2.09, 2.13) |
| Provider type |  |  |  |  |  |  |
| General MD | 3,461,138 (84.0%) | 1,250,897 (82.3%) | 1,865,918 (83.6%) | 1,515,120 (87.0%) | 138,150 (90.7%) | Ref |
| Medical specialist | 84,040 (2.0%) | 50,265 (3.3%) | 45,022 (2.0%) | 35,416 (2.0%) | 2,088 (1.4%) | 0.79 (0.79, 0.80) |
| ED MD | 252,282 (6.1%) | 123,695 (8.1%) | 100,504 (4.5%) | 75,820 (4.4%) | 7,596 (5.0%) | 1.25 (1.24, 1.26) |
| ENT MD | 107,140 (2.6%) | 41,650 (2.7%) | 36,379 (1.6%) | 31,520 (1.8%) | 2,249 (1.5%) | 1.22 (1.21, 1.23) |
| NP | 172,326 (4.2%) | 40,571 (2.7%) | 117,307 (5.3%) | 41,413 (2.4%) | 1,174 (0.8%) | 1.20 (1.20, 1.21) |
| PA | 43,110 (1.0%) | 11,955 (0.8%) | 66,446 (3.0%) | 41,493 (2.4%) | 1,026 (0.7%) | 0.50 (0.50, 0.51) |
| Care location |  |  |  |  |  |  |
| Regular office visit | 3,827,664 (92.9%) | 1,420,264 (93.5%) | 2,061,019 (92.4%) | 1,585,412 (91.1%) | 139,894 (91.9%) | Ref |
| Urgent care | 114,642 (2.8%) | 38,694 (2.5%) | 74,257 (3.3%) | 95,035 (5.5%) | 5,616 (3.7%) | 0.78 (0.78, 0.79) |
| Walk-in retail clinic | 2,578 (0.1%) | 491 (0.0%) | 3,734 (0.2%) | 662 (0.0%) | 2 (0.0%) | 0.73 (0.69, 0.76) |
| ED | 175,152 (4.3%) | 59,584 (3.9%) | 92,566 (4.1%) | 59,673 (3.4%) | 6,771 (4.4%) | 1.03 (1.02, 1.04) |
| DM | 258,441 (6.3%) | 86,625 (5.7%) | 121,890 (5.5%) | 84,457 (4.9%) | 9,426 (6.2%) | 1.13 (1.13, 1.14) |
| HTN | 692,315 (16.8%) | 222,259 (14.6%) | 307,445 (13.8%) | 192,669 (11.1%) | 25,337 (16.6%) | 1.60 (1.59, 1.61) |
| Stroke | 20,831 (0.5%) | 10,484 (0.7%) | 10,423 (0.5%) | 5,934 (0.3%) | 668 (0.4%) | 0.98 (0.96, 0.99) |
| Kidney dysfunction | 17,771 (0.4%) | 6,039 (0.4%) | 8,309 (0.4%) | 7,651 (0.4%) | 655 (0.4%) | 1.09 (1.07, 1.12) |
| Liver disease | 52,717 (1.3%) | 24,735 (1.6%) | 25,394 (1.1%) | 24,900 (1.4%) | 2,058 (1.4%) | 0.95 (0.94, 0.96) |
| Dementia | 4,710 (0.1%) | 2,059 (0.1%) | 2,560 (0.1%) | 1,905 (0.1%) | 159 (0.1%) | 1.03 (0.99, 1.07) |
| Obesity | 135,499 (3.3%) | 49,714 (3.3%) | 66,377 (3.0%) | 55,482 (3.2%) | 5,013 (3.3%) | 0.97 (0.96, 0.98) |
| Heart failure | 13,816 (0.3%) | 5,332 (0.4%) | 6,561 (0.3%) | 4,255 (0.2%) | 467 (0.3%) | 1.18 (1.15, 1.21) |
| Ischemic heart disease | 81,914 (2.0%) | 32,277 (2.1%) | 42,121 (1.9%) | 20,554 (1.2%) | 2,685 (1.8%) | 1.07 (1.06, 1.08) |
| Atrial fibrillation | 18,339 (0.4%) | 8,722 (0.6%) | 11,133 (0.5%) | 7,183 (0.4%) | 723 (0.5%) | 0.97 (0.95, 1.00) |
| VTE | 6,185 (0.2%) | 3,050 (0.2%) | 4,271 (0.2%) | 2,488 (0.1%) | 254 (0.2%) | 0.83 (0.80, 0.86) |
| Urinary tract infections | 256,354 (6.2%) | 87,428 (5.8%) | 119,139 (5.3%) | 99,073 (5.7%) | 9,455 (6.2%) | 1.05 (1.04, 1.06) |
| HIV/AIDS | 8,182 (0.2%) | 2,841 (0.2%) | 2,273 (0.1%) | 3,293 (0.2%) | 196 (0.1%) | 1.54 (1.49, 1.58) |
| Fractures | 47,717 (1.2%) | 19,481 (1.3%) | 27,539 (1.2%) | 21,877 (1.3%) | 1,798 (1.2%) | 0.92 (0.91, 0.93) |
| Falls | 10,456 (0.3%) | 5,550 (0.4%) | 6,450 (0.3%) | 6,144 (0.4%) | 554 (0.4%) | 0.77 (0.75, 0.79) |
| GERD | 157,906 (3.8%) | 59,786 (3.9%) | 75,583 (3.4%) | 53,426 (3.1%) | 6,408 (4.2%) | 1.08 (1.07, 1.09) |
| Peptic ulcer disease | 8,633 (0.2%) | 3,468 (0.2%) | 3,919 (0.2%) | 3,353 (0.2%) | 316 (0.2%) | 1.02 (0.99, 1.05) |
| Major bleeding events | 14,633 (0.4%) | 6,800 (0.4%) | 8,388 (0.4%) | 6,723 (0.4%) | 596 (0.4%) | 0.90 (0.86, 0.93) |
| Bronchiectasis | 585 (0.0%) | 267 (0.0%) | 268 (0.0%) | 263 (0.0%) | 22 (0.0%) | 1.21 (1.09, 1.35) |
| Connective tissue diseases | 10,838 (0.3%) | 4,741 (0.3%) | 6,132 (0.3%) | 4,717 (0.3%) | 388 (0.3%) | 0.98 (0.93, 1.03) |
| Use of NSAIDs | 530,328 (12.9%) | 158,137 (10.4%) | 256,708 (11.5%) | 215,977 (12.4%) | 16,701 (11.0%) | 1.10 (1.10, 1.11) |
| Use of PPI | 296,516 (7.2%) | 103,960 (6.8%) | 156,185 (7.0%) | 105,947 (6.1%) | 10,043 (6.6%) | 1.01 (1.01, 1.02) |
| Use of H2RA | 37,156 (0.9%) | 13,532 (0.9%) | 19,127 (0.9%) | 23,244 (1.3%) | 1,376 (0.9%) | 0.84 (0.83, 0.85) |
| Use of antibiotics | 1,297,308 (31.5%) | 437,638 (28.8%) | 657,603 (29.5%) | 497,547 (28.6%) | 40,136 (26.4%) | 1.09 (1.09, 1.09) |
| Use of antiplatelets | 41,773 (1.0%) | 13,285 (0.9%) | 21,948 (1.0%) | 12,057 (0.7%) | 1,306 (0.9%) | 1.09 (1.07, 1.10) |
| Use of anticoagulants | 21,023 (0.5%) | 8,493 (0.6%) | 13,564 (0.6%) | 8,500 (0.5%) | 728 (0.5%) | 0.98 (0.96, 1.00) |
| Combined co-morbidity score | |  |  |  |  |  |
| < 1 | 3,164,427 (76.8%) | 1,168,690 (76.9%) | 1,764,636 (79.1%) | 1,411,799 (81.1%) | 116,391 (76.4%) | Ref |
| 1 - 2 | 766,452 (18.6%) | 273,372 (18.0%) | 375,416 (16.8%) | 263,539 (15.1%) | 28,568 (18.8%) | 0.86 (0.85, 0.86) |
| 2 - 4 | 167,079 (4.1%) | 68,104 (4.5%) | 80,537 (3.6%) | 57,885 (3.3%) | 6,483 (4.3%) | 0.78 (0.78, 0.79) |
| >= 4 | 22,078 (0.5%) | 8,867 (0.6%) | 10,987 (0.5%) | 7,559 (0.4%) | 841 (0.6%) | 0.72 (0.70, 0.74) |
| Employment status |  |  |  |  |  |  |
| active full time; n (%) | 2,311,118 (56.1%) | 834,709 (55.0%) | 1,210,332 (54.2%) | 1,133,576 (65.1%) | 18,735 (12.3%) | Ref |
| others/unknown; n (%) | 1,578,684 (38.3%) | 598,195 (39.4%) | 844,709 (37.9%) | 494,155 (28.4%) | 132,199 (86.8%) | 1.12 (1.11, 1.12) |
| retiree; n (%) | 194,076 (4.7%) | 68,708 (4.5%) | 149,978 (6.7%) | 89,449 (5.1%) | 1,062 (0.7%) | 0.91 (0.91, 0.92) |
| active part-time; n (%) | 36,158 (0.9%) | 17,421 (1.1%) | 26,557 (1.2%) | 23,602 (1.4%) | 287 (0.2%) | 0.73 (0.72, 0.74) |
| Plan indicator |  |  |  |  |  |  |
| PPO | 2,621,590 (63.6%) | 787,885 (51.9%) | 1,495,831 (67.0%) | 942,672 (54.2%) | 112,082 (73.6%) | Ref |
| Comprehensive | 47,472 (1.2%) | 32,302 (2.1%) | 83,968 (3.8%) | 14,941 (0.9%) | 852 (0.6%) | 0.47 (0.46, 0.47) |
| EPO | 40,159 (1.0%) | 42,537 (2.8%) | 14,682 (0.7%) | 32,156 (1.8%) | 226 (0.1%) | 0.59 (0.59, 0.60) |
| HMO | 542,357 (13.2%) | 160,829 (10.6%) | 225,821 (10.1%) | 481,424 (27.7%) | 21,101 (13.9%) | 0.76 (0.76, 0.77) |
| POS | 392,057 (9.5%) | 182,652 (12.0%) | 127,038 (5.7%) | 87,860 (5.0%) | 13,644 (9.0%) | 1.22 (1.22, 1.23) |
| CDHP | 258,333 (6.3%) | 60,324 (4.0%) | 155,479 (7.0%) | 75,710 (4.3%) | 1,010 (0.7%) | 1.21 (1.20, 1.21) |
| HDHP | 114,016 (2.8%) | 54,282 (3.6%) | 99,537 (4.5%) | 70,938 (4.1%) | 2,799 (1.8%) | 0.70 (0.70, 0.71) |
| Others/missing | 104,052 (2.5%) | 198,222 (13.0%) | 29,220 (1.3%) | 35,081 (2.0%) | 569 (0.4%) | 0.51 (0.50, 0.51) |
| Year of Cohort Entry Date | |  |  |  |  |  |
| 2007 | 705,588 (17.1%) | 149,440 (9.8%) | 292,566 (13.1%) | 215,306 (12.4%) | 5,168 (3.4%) | Ref |
| 2008 | 540,409 (13.1%) | 113,862 (7.5%) | 346,657 (15.5%) | 189,668 (10.9%) | 4,354 (2.9%) | 0.77 (0.76, 0.77) |
| 2009 | 528,528 (12.8%) | 170,322 (11.2%) | 338,049 (15.1%) | 223,020 (12.8%) | 32,903 (21.6%) | 0.62 (0.62, 0.62) |
| 2010 | 376,920 (9.1%) | 205,352 (13.5%) | 225,980 (10.1%) | 180,088 (10.3%) | 2,886 (1.9%) | 0.59 (0.59, 0.59) |
| 2011 | 385,504 (9.4%) | 197,615 (13.0%) | 219,321 (9.8%) | 199,531 (11.5%) | 38,714 (25.4%) | 0.56 (0.55, 0.56) |
| 2012 | 453,957 (11.0%) | 212,994 (14.0%) | 231,361 (10.4%) | 185,654 (10.7%) | 20,300 (13.3%) | 0.65 (0.64, 0.65) |
| 2013 | 306,101 (7.4%) | 140,908 (9.3%) | 175,741 (7.9%) | 196,665 (11.3%) | 18,229 (12.0%) | 0.53 (0.52, 0.53) |
| 2014 | 293,004 (7.1%) | 131,951 (8.7%) | 163,751 (7.3%) | 141,496 (8.1%) | 27,899 (18.3%) | 0.57 (0.56, 0.57) |
| 2015 | 260,198 (6.3%) | 98,841 (6.5%) | 115,014 (5.2%) | 101,486 (5.8%) | 1,078 (0.7%) | 0.76 (0.76, 0.76) |
| 2016 | 269,827 (6.5%) | 97,748 (6.4%) | 123,136 (5.5%) | 107,868 (6.2%) | 752 (0.5%) | 0.77 (0.76, 0.77) |
| *Adjusted for all the variables listed in this table. Odds ratio [OR] comparing south region to all other regions. ENT= otolaryngology, DM= diabetes mellites, VTE= venous thromboembolism, HIV/AIDS=acquired immune deficiency syndrome, GERD=gastroesophageal reflux disease,  NSAIDs=nonsteroidal anti-inflammatory drugs, PPIs=proton-pump inhibitors, H2RA=histamine 2 receptor antagonists, COBRA=Consolidated Omnibus Budget Reconciliation Act, PPO=preferred provider organization , HMO=health maintenance organization, EPO= exclusive provider organization, POS=point of service, CDHP= consumer-driven health plan, HDHP= high-deductible health plan | | | | | | |
