# Supplementary material for: Prescribing systemic steroids for acute respiratory tract infections in United States outpatient settings: A nationwide population-based cohort study
Source: PLoS Med. 2020 Mar 31;17(3):e1003058. doi: 10.1371/journal.pmed.1003058 (PMC7108689; doi:10.1371/journal.pmed.1003058)
Supplement: Attachment — Submitted filename: Response to reviewers_22Nov2019_final.docx [file pmed.1003058.s006.docx]

**Response to the Editors and Reviewers (page numbers referred to were in the marked-up version)**

**Requests from the editors:**

**1- Did your study have a prospective protocol or analysis plan? Please state this (either way) early in the Methods section.**

**Response:** We do have a study protocol which described the analysis plan prior to analyzing the data. We have included it in Appendix 1 and referenced it in the manuscript (1^st^ paragraph on page 6).

**2- Please ensure that the study is reported according to the RECORD guideline, and include the completed RECORD checklist as Supporting Information. Please add the following statement, or similar, to the Methods: "This study is reported as per the REporting of studies Conducted using Observational Routinely-collected health Data (RECORD) guideline (S1 Checklist)."**

**Response:** We have completed RECORD checklist and referenced it in the text (1^st^ paragraph on page 6).

**3- Please add this statement to the manuscript's Competing Interests: "ASK is an Academic Editor on PLOS Medicine's editorial board."**

**Response:** This is now added.

**4- Please edit your data statement to provide details on how readers may contact IBM MarketScan for the data used in this study, noting any restrictions that may apply.**

**Response:** This is now done accordingly.

**5- Please place the study design in the subtitle (ie, after a colon).**

**Response:** Study design is now one of the sub-headings on 3^rd^ paragraph on page 5. Note that because the information on study design is very much related to data sources, their contents are combined.

**6- Please combine the Methods and Findings sections of the Abstract into one section, “Methods and findings”.**

**Response:** It was done accordingly.

**7- In the last sentence of the Abstract Methods and Findings section, please describe the main limitation(s) of the study's methodology.**

**Response:** It was done accordingly.

**8- Please remove the ® trademark symbol throughout the manuscript.**

**Response:** It was done accordingly.

**9- In the Abstract Methods and findings, please include some brief statistics on the included cohort (e.g., age, sex)**

**Response:** Revised accordingly.

**10- In the Abstract and going forward, please include p values alongside the 95% CIs.**

**Response:** Revised accordingly.

**11- At this stage, we ask that you include a short,** **non-technical Author Summary of your research to make findings accessible to a wide audience that includes both scientists and non-scientists. The Author Summary should immediately follow the Abstract in your revised manuscript. This text is subject to editorial change and should be distinct from the scientific abstract.**

**Response:** We have added the non-technical Author Summary.

**12- In the Introduction, please cite a specific guideline or guidelines re prescribing, rather than "an observational study”.**

**Response:** We have cited Adult Treatment Recommendations from CDC (<https://www.cdc.gov/antibiotic-use/community/for-hcp/outpatient-hcp/adult-treatment-rec.html>) and two published guidelines (Am Fam Physician. 2010;82(11):1345-50 & Am Fam Physician. 2012;86(2):153-9) in the 2^nd^ and 3^rd^ paragraph on page 4.

**13- Please include information on ethical approval informed consent in the Methods.**

**Response:** It is included in 1^st^ paragraph on page 6.

**14- Table 2 provides information on gender. Is this accurate or did you mean sex?**

**Response:** We have now replaced “gender” with “sex”.

**15- Though perhaps not the intent, the language of "non evidence based" can be read in rather a sanctimonious way, considering that some of the prescribing appears to be for symptomatic relief in the ED. In the discussion, it could be worth mentioning the alternatives available to doctors for provision of symptomatic relief.**

**Response:** We have toned down our discussion and abstract by using the language of “a practice without clear net benefit”, “lacking clear scientific justification” “very little evidence supporting…” rather than “non-evidence based”. We think mentioning available alternatives falls out of the scope of this paper as there could be a whole host of alternatives -- saline irrigation for sinusitis, antibiotics for sinusitis, benzonatate for cough, inhalers for bronchoconstriction, nonsteroidal anti-inflammatory drugs for pain, etc).

**Comments from the reviewers:**

**Reviewer #1: Alex McConnachie, Statistical Review**

**The article by Lin et al presents an analysis of routine data from approximately 10 million people, to investigate the use of systemic steroids for acute respiratory tract infections over a 10-year period in the US. This review is primarily focused on the use of statistics in the paper.**

**The description of the study population is good, giving a clear description of those included and excluded, and why. There is a long list of patient and encounter characteristics that were used to adjust the analyses, all of which appear sensible things to look at. The study outcome is clearly defined. Even though the length of follow-up is not the same for every study participant, given the size of the dataset, and the average length of follow-up, the use of logistic regression is justified. The sensitivity analyses also seem reasonable.**

**When looking at the associations between ARTI conditions and systemic steroid prescribing, care must be taken, since every patient had at least one condition. So, to say that having influenza was associated with a reduced likelihood of a prescription may not be accurate, since those without a diagnosis of influenza must have had one of the other diagnoses.**

**Response :** We have revised the result section to read “*Among patients with an ARTI diagnosis in an outpatient setting, those diagnosed with acute bronchitis was associated with the highest odds of receiving systemic steroid (adjusted odds ratio [aOR]: 2.70, 95% confidence interval [CI]: 2.68-2.72, p <0.01), followed by acute sinusitis (aOR 2.03, 95% CI: 2.02-2.04, p <0.01), pneumonia (aOR 1.80, 95% CI: 1.77-1.82, p <0.01), allergic rhinitis (aOR 1.74, 95% CI: 1.72-1.75, p <0.01), otitis media (aOR 1.43, 95% CI: 1.42-1.45, p <0.01), pharyngitis (aOR 1.42, 95% CI: 1.41-1.43, p <0.01), and acute upper respiratory infections (aOR 1.23, 95% CI: 1.22-1.24, p <0.01). Among those with an ARTI diagnosis, having a diagnosis of influenza was associated with lower odds of receiving systemic steroids (aOR 0.65, 95% CI: 0.64-0.66, p <0.01; Table 2).*” (1^st^ paragraph on page 9)

**Data are presented for parenteral and oral steroids, but the methods section describes extracting the route of administration as oral, intravenous, or intramuscular. Is there a reason for this?**

**Response:** The parenteral routes include intravenous and intramuscular forms. However, majority of these uses were “non-specific injections” without further details to distinguish intravenous vs. intramuscular forms. We have now revised the method text to read “*The primary outcome was defined as having a dispensing record or a procedure code indicating use of systemic steroids orally and parenterally (including intravenous, intramuscular, and non-specific injectable forms) within 7 days of cohort entry date.*” (3^rd^ paragraph on page 7)

**The sensitivity analysis results are reportedly similar to the main analysis, but these results are not shown. Would it be possible to include these results in the supplement, just for completeness?**

**Response:** The results of the sensitivity analysis have been added to Appendix 2 and cited as such (1^st^ paragraph on page 11).

**Given that one of the most striking findings of this analysis is the** **geographical variation in prescribing rates, I wonder whether it would be of value to carry out stratified analyses, or even to formally test whether the predictors of prescribing vary between geographical regions. For example, the patterns of prescribing in relation to age and gender could be very different in different regions. It may be too much for this paper, but perhaps worth considering.**

**Response:** Following the reviewer’s suggestion, we have added some quantitative data to support the claim that the association between region and steroid prescribing cannot be explained by the modest differences overserved in patient characteristics across regions. We have added the supporting data in Appendix 3 and the following text on the 3^rd^ paragraph on page 11: “*We did not find any meaningful differences in patient demographics, ARTI indications, care settings, provider types, and patient co-morbidities that can explain the remarkable geographical variability in steroid use for ARTI (Appendix 3). To put geographical variations of patient characteristics into perspective, the observed risk ratio of 3.78 associated with the southern region could only be fully explained by an unmeasured confounder that was associated with both the southern region and steroid prescribing by a risk ratio of 7.02-fold each[24], above and beyond the adjusted factors. By the same formula, only an unmeasured confounder associated with both the southern region and steroid prescribing by a risk ratio of 28.45-fold each could account for the 14-fold increased odds of receiving parenteral steroids in the South[24]. The adjusted odds ratios comparing the southern region to all other regions were far smaller than these required thresholds (Appendix 3).”*

**Table 2 is fine, but I think it could be improved. What matters are the numbers of patients in each row category, and the number and percentage who received a steroid prescription. E.g. for the age group "18 - <25", I would show in the first column, that there were 1,752,290 people in this category, and in the second column, show that 184,968 (10.6%) received a steroid. Since the outcome is receiving a steroid prescription, then as a reader I want to see how the percentage with the outcome varies according to patient and encounter characteristics.**

**Response:** Table 2 has been revised accordingly.

**Reviewer #2: This paper uses a large medications database to explore the use of oral and systemic steroids for ARTI in the US. It is a clear and well written paper. I have a number of minor 'style comments' detailed below. For the non US reader a slightly fuller explanation of the claims database might be helpful - what proportion of consultations in the US are captured by this. What is missed in such a dataset (non-insured/other settings?)**

**Response:** We have added some additional description about IBM MarketScan database in the 3^rd^ paragraph on page 5.

**The analysis adjusted for many co-variates although these were not clearly justified in the manuscript, a sentence to justify these would be helpful.**

**Response:** All the analysis plans and definitions were specified prior to study implementation. This has been stated in the 1^st^ paragraph on page 6. The study protocol is now available as Appendix 1 in the supplemental information online.

**The final paragraph on p 11 (potential explanations) might be better placed later in the manuscript.**

**Regarding the first paragraph on page 12 I think it is sufficient to say that the issue should be addressed, and appropriate interventions developed using the models shown to be effective in antibiotic stewardship rather than selecting academic detailing for specific mention.**

**Response:** The explanation was mainly for increasing prescribing rates over time, thus immediately following the findings about the time trend. With regard to the comments on academic detailing, we have replaced it with a more generic term “effective medical education program” (2^nd^ paragraph on page 14).

**Style Comments**

**Background: I suggest leaving the reader to draw conclusions about whether steroid prescribing is questionable and that the first sentence could simply read: “In the outpatient settings is not recommended in clinical guidelines”**

**Response:** Revised accordingly.

**Para 3- why not say Despite the lack of evidence one recent review….**

**Response:** Revised accordingly.

**Some minor wording comments, I don’t think 'drastic' is needed as a descriptor and when discussing steroids in sinusitis- the treatment effect could simply be explained rather than 'explained away'**

**Response:** We have replaced “drastic geographical variability” with “remarkable geographical variability” and used “explained” rather than “explained away” as suggested.

**Reviewer #3:**

1.	**Novelty: Previous studies have demonstrated high levels of systemic corticosteroid use among patients with ARTI with rates comparable to those demonstrated in this study, albeit using different methods. Could the authors highlight in the discussion where this study truly adds novel findings?**

**Response:** This is the first study to demonstrate the nation-wide remarkable geographical variability in steroid prescribing for ARTI and time trend in the past decade. These findings have been emphasized in the 2^nd^ paragraph of discussion (page 11). With regard to the estimates of prescribing rate for ARTI, although it was reported before, the prior report was based on survey data, which may be subject to recall inaccuracy, particularly regarding details of medication use, such as dose and duration of the prescription. Therefore the current study provides more robust estimates with substantially more details, including administrative routes, dose, and other covariates (3^rd^ paragraph on page 4).

**2.	Potential for misclassification: The use of systemic corticosteroids for the treatment of ARTI was the primary outcome. The potential for misclassification may be problematic at the level of the ARTI diagnosis itself and the at the level of systemic corticosteroid indication. The diagnosis of ARTI included acute bronchitis, sinusitis, pharyngitis, otitis media, allergic rhinitis, influenza, pneumonia, and unspecified acute upper respiratory infections. Have the codes for identifying these conditions been validated? If so, please provide references. The assessment indication of systemic corticosteroid involved numerous inclusion / exclusion criteria since systemic corticosteroids are widely used for a variety of indications. Has the approach to assessing systemic corticosteroid use for the purpose of treating ARTIs been validated? If so, please provide a reference. The authors do acknowledge potential misclassification as a limitation.**

**Response:** Our study outcome was prescribing of systemic steroids. It was assessed using the comprehensive library of National Drug Code (NDC), generic, and trade names of all systemic steroids (the details of the definition are available in the Appendix 1) based on dispensing records and procedure code for steroid injections. Electronic outpatient pharmacy dispensing records are considered accurate because pharmacists fill prescriptions with little room for interpretation, and are reimbursed by insurers on the basis of detailed, complete, and accurate claims submitted electronically [Can J Clin Pharmacol. 2003;10(2):67-71]. Pharmacy dispensing information is usually seen as the gold standard of drug exposure information compared to self-reported information [Am J Epidemiol. 1995;142(10):1103-12] or prescribing records in outpatient medical records [J Clin Epidemiol. 1994;47(2):165-71]. Having a procedure code indicates the treatment was done on-site and it would be considered a fraud if it was not implemented. As claims data were generated for reimbursement purposes, such codes were considered highly reliable. These references were listed in the method sections (3^rd^ paragraph on page 5 and 1^st^ paragraph on page 6). With regard to the inclusion and exclusion criteria, we used definitions in the published study whenever available [JAMA Intern Med. 2018;178(6):852-4 (an honorable mention article in JAMA Internal Medicine 2019 Update on Medical Overuse review), Circulation. 2019 Jun 18;139(25):2822-2830, [Pharmacoepidemiol Drug Saf.](https://www.ncbi.nlm.nih.gov/pubmed/?term=Pharmacoepidemiology+and+drug+safety+2012%3B+21(S1)%3A+154+162) 2012 Jan;21 Suppl 1:154-62, BMC Musculoskelet Disord. 2015 Sep 3;16:237]. These references were also added (1^st^ paragraph on page 6). As none of these algorithms is perfect, we acknowledged that misclassification of our study variables is possible. However, given that we excluded additionally those who received systemic steroids in the year prior to the cohort entry, our estimated prescribing rates for ARTI is probably conservative (1^st^ paragraph on page 15).

**3.	Generalizability: Approximately 9.7 million patients of over 41 million patients met the inclusion / exclusion criteria (i.e. 24%), which may limit generalizability. Have the authors investigated the generalizability of their findings? They have noted this as a limitation in the discussion section.**

**Response:** We believe it is important to exclude patients with steroid-indicated conditions so that we will not attribute legitimate steroid prescribing to ARTI indications. Therefore, our findings are not intended to be generalized to patients with these conditions and we have made it clear as a limitation in the 2^nd^ paragraph on page 14.

**4.	A priori definition of outcomes and exposure assessment: Geography was a key exposure that was being assessed yet it is poorly described in the methods section. How was geography defined? It's assumed that this was done at the state level. Did all states have comparable database coverage (i.e. was sampling similar from state to state)? Further, were there any pre-specified hypotheses? Tables 2 and 3 report a very large number of statistical tests. If not, I worry this may simply be a 'fishing expedition' that would be subject to false associations related to multiple hypothesis testing. Inclusion of a formal analysis plan in the methods section is highly advised.**

**Response:** Geography was defined at the state level. In IBM MarketScan database, there are more than 300 contributing employers and 25 contributing health plans nationwide. No sampling was done by geographical region and all states are well-represented. Regarding pre-specified hypothesis, we state in the introduction that we aimed to assessed time trend and geographical region are significant predictors of steroid prescribing for ARTI based on prior data (3^rd^ paragraph on page 4). Also, we had planned to assess other potential predictors for such prescribing and all the covariates were pre-specified in our protocol before the implementation of the analysis (protocol available in Appendix 1).
