# Supplementary material for: Prescribing systemic steroids for acute respiratory tract infections in United States outpatient settings: A nationwide population-based cohort study
Source: PLoS Med. 2020 Mar 31;17(3):e1003058. doi: 10.1371/journal.pmed.1003058 (PMC7108689; doi:10.1371/journal.pmed.1003058)
Supplement: Attachment — Submitted filename: Response to the editors and reviewers_R2.docx [file pmed.1003058.s007.docx]

**Response to the Editors and Reviewers (page numbers referred to were in the marked-up version)**

**Requests from Editors:**

**Please revise your title according to PLOS Medicine's style. Your title must be nondeclarative and not a question. It should begin with main concept if possible. "Effect of" should be used only if causality can be inferred, i.e., for an RCT. Please place the study design ("A randomized controlled trial," "A retrospective study," "A modelling study," etc.) in the subtitle (ie, after a colon).**

**Please add a study descriptor to the title after a colon, e.g., "... settings: a cohort study"**

Response: We have changed our title to “Prescribing Systemic Steroids for Acute Respiratory Tract Infections in US Outpatient Settings: A Nationwide Population-based Cohort Study”.

**In your abstract, please remove the word "remarkable" or substitute "high", say. Please add some summary demographic details and the final sentence of the ‘Methods and Findings’ section should include a sentence on the limitations of the study. Please provide p values where 95% Cis are given.**

Response: This was done accordingly.

**Please begin the "conclusions" subsection of your abstract with "In this study, we found that systemic steroid use ... was common ..." or similar.**

Response: Revised according.

**Author summary needs reformatting to bullet points, per house style**

**Page 4 – please add subheading of Introduction**

Response: Revised according.

**As the statistician has queried the analysis plan, please add a sentence in the methods to say when it was prepared.**

Response: We have specified in the 1^st^ paragraph on page 6 “*All analysis plans and definitions were specified prior to study implementation*.”

***p*<0.01 to be replaced with p<0.001 or exact values, please**

Response: Revised according.

**Square brackets to be relocated to before punctuation, throughout.**

Response: Done accordingly

**Comments from Reviewers:**

**Reviewer #1: Alex McConnachie, Statistical Review**

**The authors have addressed all of my original comments. In Table 2, I was perhaps not clear enough. I was looking for the row percentages, so that, for example, for males, the percentage who received steroids was 12.6% (540236/4297795), and for women, 11.2%. I believe this makes it clearer that men were more likely to receive steroids than women.**

Response: Done accordingly.

**In response to one of the editor's points, the authors have provided a protocol. The analysis section of this document is very brief compared to most protocols, and, oddly, is written in the past tense.**

Response: We have provided more details in the analysis plan in the protocol and change the tense as suggeseted.
